# Specific suppression of insulin sensitivity in *growth hormone receptor* gene-disrupted (GHR-KO) mice attenuates phenotypic features of slow aging

**DOI:** 10.1111/acel.12262

**Published:** 2014-09-20

**Authors:** Oge Arum, Ravneet K Boparai, Jamal K Saleh, Feiya Wang, Angela L Dirks, Jeremy G Turner, John J Kopchick, Jun-Li Liu, Romesh K Khardori, Andrzej Bartke

**Affiliations:** 1 Department of Internal Medicine, Southern Illinois University-School of MedicineSpringfield, IL, 62794, USA; 2 Division of ENT-Otolaryngology, Department of Surgery, Southern Illinois University-School of MedicineSpringfield, IL, 62794, USA; 3 Edison Biotechnology Institute and Department of Biomedical Sciences, Heritage College of Osteopathic Medicine, Ohio UniversityAthens, OH, 45701, USA; 4 Fraser Laboratories for Diabetes Research, Department of Medicine, McGill University Health Centre687 Pine Avenue West, Montreal, QC, H3A 1A1, Canada; 5 Division of Endocrinology & Metabolism, Department of Internal Medicine, Eastern Virginia Medical School700 West Olney Road, Norfolk, VA, 23507, USA

**Keywords:** endocrinology and metabolism, growth hormone hormonal signaling, insulin sensitivity, longevity regulation, (neuro)endocrinology of senescence

## Abstract

In addition to their extended lifespans, slow-aging *growth hormone receptor/binding protein* gene-disrupted (knockout) (GHR-KO) mice are hypoinsulinemic and highly sensitive to the action of insulin. It has been proposed that this insulin sensitivity is important for their longevity and increased healthspan. We tested whether this insulin sensitivity of the GHR-KO mouse is necessary for its retarded aging by abrogating that sensitivity with a transgenic alteration that improves development and secretory function of pancreatic β-cells by expressing *Igf-1* under the rat *insulin* promoter 1 (RIP::IGF-1). The RIP::IGF-1 transgene increased circulating insulin content in GHR-KO mice, and thusly fully normalized their insulin sensitivity, without affecting the proliferation of any non-β-cell cell types. Multiple (nonsurvivorship) longevity-associated physiological and endocrinological characteristics of these mice (namely beneficial blood glucose regulatory control, altered metabolism, and preservation of memory capabilities) were partially or completely normalized, thus supporting the causal role of insulin sensitivity for the decelerated senescence of GHR-KO mice. We conclude that a delayed onset and/or decreased pace of aging can be hormonally regulated.

## Introduction

The unprecedented demographic shift caused by the aging of the Baby Boomer generation is resulting in a tidal increase in the absolute number and relative proportion of older and elderly persons. Accompanying increased age are increased frailty, disability, inability to perform activities of daily living and age-related disease, leading to increased cost of health care (Knickman & Snell, 2002; Cangelosi, 2011; Ricketts, 2011). This onus is public and private, financial and psychological, affecting all age groups of taxpayers. Yet this trend is not inevitable, as there are mammalian models of the type of ameliorated senescence that would help to allay these concerns (Chen *et al*., 2010; Selman & Withers, 2011). Studies that address the development of pharmacological or lifestyle interventions for the retention of health and functional independence in the aging are therefore timely and important.

Insulin sensitivity, defined as the efficacy and kinetics of glucose clearance from the blood, is highly positively correlated to modifications of longevity, whether induced by genetic or dietary interventions (Barbieri *et al*., 2001, 2003, 2009; DiStefano *et al*., 2007; Bartke, 2008; Barzilai & Bartke, 2009; Redman & Ravussin, 2009; Avogaro *et al*., 2010), and many studies of long-lived mutants have investigated their insulin sensitivity and related it to their enhanced survivorship (Brown-Borg *et al*., 1996; Dominici *et al*., 2002; Bonkowski *et al.,* 2006; Conover & Bale, 2007; Conover *et al*., 2008; Selman *et al*., 2008; Arum *et al*., 2009). Although there is a wealth of data showing a clear association between the two, the proffered mechanisms for how insulin sensitivity might engender longevity are few (Masoro, 2005; Bartke, 2008; Arum *et al*., 2009), and those that have been proposed remain untested. Endeavoring to address multiple aging-associated maladies by study of the basic biology of longevity, as outlined in the concept of the Longevity Dividend (Olshansky *et al*., 2007; Miller, 2009; Warner & Sierra, 2009; Kenyon, 2010), we investigated the positive association between insulin sensitivity and retained healthspan.

The mouse homozygous for a targeted disruption (knockout, KO) of the *growth hormone* (GH) *receptor* (GHR)*/binding protein* gene (the GHR-KO mouse) is GH-resistant and, thus, has decreased GH signaling. GHR-KO mice were generated by insertional mutagenesis that disrupted the *growth hormone receptor/binding protein (Ghr/bp)* gene. This results in decreased hepatic production of insulin-like growth factor 1 (IGF-1), which leads to markedly reduced levels of circulating IGF-1, reduced growth rate, an approximately 20% reduction in adulthood length, and an approximately 40% reduction in adult body weight (Zhou *et al*., 1997).

In addition, the GHR-KO mouse lives approximately 40% longer than its littermate control, exhibits enhanced retention of learning and memory capabilities into middle and old age, shows a lower incidence of tumors upon death, and displays other marks of enhanced healthspan (Berryman *et al*., 2008; Bartke, 2011), establishing it as legitimately slow senescing. Its longevity has been characterized on three different genetic backgrounds (Coschigano *et al*., 2000, 2003; Bonkowski *et al*., 2006), on 30% caloric restriction (Bonkowski *et al*., 2006), on intermittent fasting (Arum *et al*., 2009), and on diets of differing composition (Bartke *et al*., 2004), making it the sole genetic model of longevity that has been validated under a variety of conditions. What is more, multiple mutants characterized by deficient GH signaling have been documented to exhibit extended longevity [*prophet of Pit1, paired-like homeodomain transcription factor* (*PROP paired-like homeobox 1*) hypomorphic point mutation (*Prop1*^*df*^) (Brown-Borg *et al*., 1996), *pituitary-specific positive transcription factor 1* (*POU domain, class 1, transcription factor 1* (*Pou1f1*)) hypomorphic point mutation (*Pit-1*^*dw*^) (Flurkey *et al*., 2001), *growth hormone-releasing hormone receptor* hypomorphic point mutation (*Ghrhr*^*lit*^) (Flurkey *et al*., 2001), and *growth hormone-releasing hormone* deletion (*Ghrh*^*−/−*^) (O. Arum, R. Salvatori & A. Bartke, unpublished observations)].

Produced in the β-cells of the pancreatic islet of Langerhans, insulin regulates metabolism and energy homeostasis; in part by engendering the uptake of glucose from the blood by target tissues. The GHR-KO mouse exhibits markedly decreased plasma insulin levels (Liu *et al*., 2004). As blood insulin concentration inversely mediates whole-body insulin sensitivity, insulin sensitivity is greater in the GHR-KO mouse than in its littermate control (Liu *et al*., 2004), as is the case with multiple other long-lived mice (Brown-Borg *et al*., 1996; Dominici *et al*., 2002; Conover & Bale, 2007; Conover *et al*., 2008; Selman *et al*., 2008).

The GHR-KO mouse has multiple, gerontologically intriguing characteristics, including increased circulating GH concentration, conversely decreased GH hormonal signaling, decreased circulating IGF-1 concentration, decreased body size, obesity, and altered endocrine function. In order to exclusively test whether the insulin sensitivity due to decreased insulin production/secretion in the GHR-KO mouse is necessary for the delayed and decreased pace of senescence of this mouse, we have used a GHR-KO mouse that carries a transgene driving expression of rat *Igf-1* under the potent, β-cell-expression-enriching rat *insulin* promoter 1 (RIP) (the GHR-KO;RIP::IGF-1 double mutant). Liu and colleagues have shown that this transgene partially corrects the reduction in pancreatic islet cell mass and size present in the GHR-KO mouse (Guo *et al*., 2005), potentially increasing blood insulin levels and thus decreasing insulin sensitivity. If decreased β-cell production and/or secretion of insulin *is* necessary for the full longevity of the GHR-KO mouse, then a GHR-KO mouse with partially normalized β-cell production of insulin should age sooner/faster than a standard GHR-KO mouse.

With these predictions in mind, we have developed mice for dissociating the effects of (i) GH resistance/hypersomatotrophinemia and (ii) circulating IGF-1 deficiency from the effects of (iii) insulin sensitivity. All three are features of the long-lived, slow-aging GHR-KO mouse, and overlap features of other slow-senescing mutants, including dietarily restricted mice. The molecular or physiological mechanism(s) for how any of the three could independently result in a retention of health have been proposed (Bartke & Brown-Borg, 2004; Longo & Finch, 2003; Bartke, 2005b; Berryman *et al*., 2008; Bartke, 2008; Barzilai & Bartke, 2009; Brown-Borg, 2009). In order to come to firmer conclusions regarding the necessity or sufficiency of any one of those features for decelerated senescence, that feature would have to be specifically ‘rescued’ (normalized) in a slow-aging mouse without effect on the other two.

Our objective is to determine whether the GHR-KO;RIP::IGF-1 mouse has (nonsurvivorship) phenotypes suggestive of normalized aging, as opposed to the slowed aging of the GHR-KO mouse. Specifically, we wish to test the hypothesis that insulin sensitivity is necessary for the slow-aging-associated features of the GHR-KO mouse. In order to investigate whether the GHR-KO;RIP::IGF-1 mouse displays the above-predicted characteristics, we have assessed the effect of the RIP::IGF-1 transgene on a variety of anatomical, physiological, endocrinological, metabolic, behavioral/neuromusculoskeletal, neurobiological, and macromolecular parameters in young–adult and/or middle-aged mice.

## Results

### Evidence of lack of nonspecific effect of RIP::IGF-1 transgene

This study is inherently predicated on the specificity of effect of the RIP::IGF-1 transgene employed. For generation of the RIP::IGF-1 transgenic construct, Liu *et al*. chose the promoter based on its high level, and β-cell-enriched, expression in driving *insulin-like growth factor 2* (*Igf-2*) and *glucose transporter 2* (*Glut-2*) antisense gene expression (Valera *et al*., 1994; Devedjian *et al*., 2000; Guo *et al*., 2005). Moreover, prior work on GHR-KO mice bearing a RIP::IGF-1 transgene detailed the ability of the transgenic manipulation to induce normalization of islet cell growth and proliferation in the inherently islet cell-deficient GHR-KO mouse (Guo *et al*., 2005). Yet, as our subjects differ in genetic background, animal husbandry, heterozygosity of control subjects for the *Ghr/bp* disruption, gender (in some experimental instances), and potentially other important respects, we extensively investigated the specificity of the anatomical and physiological effects of the putatively β-cell-expression-enriched transgene.

**Figure 1:**
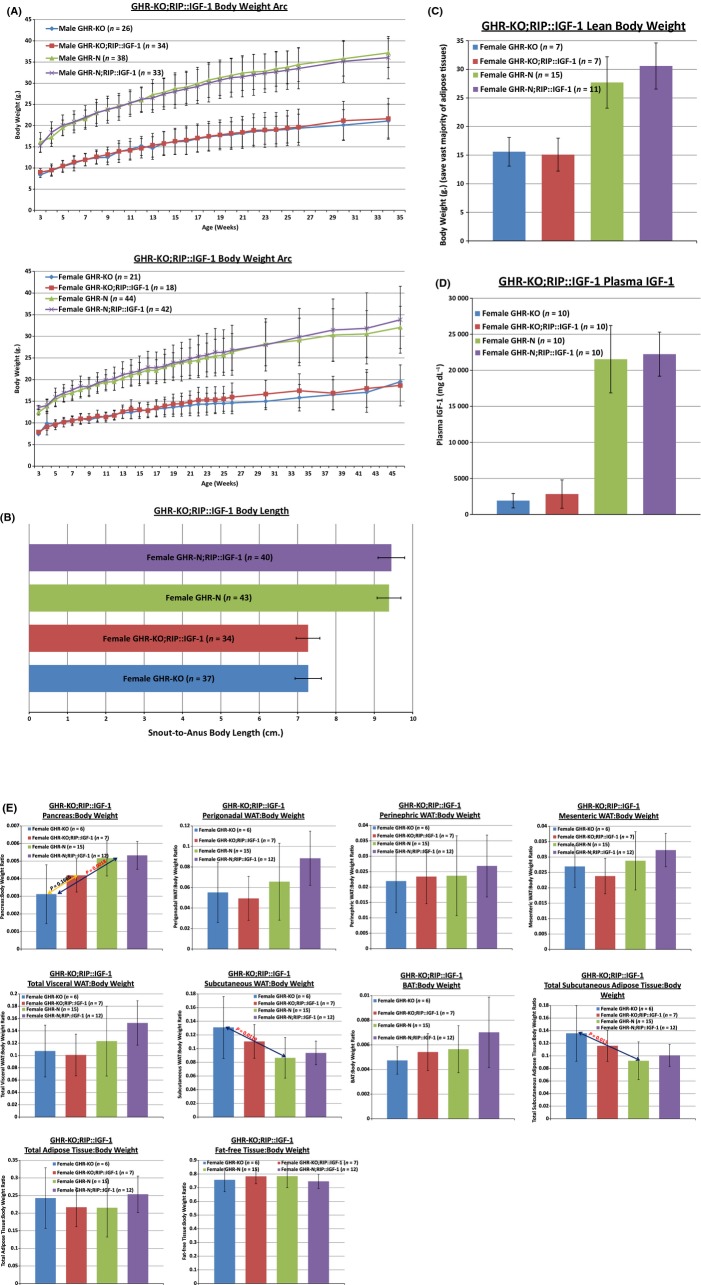
The RIP::IGF-1 Transgene Does Not Affect any of Multiple Measures of Proliferation or Growth within the GHR-KO Mouse. (A) Body weight measurements showed no effects of the RIP::IGF-1 transgene for either male (upper graph) or female (lower graph) mice of either *Ghr/bp* genotype. (B) Body length measurements revealed that the RIP::IGF-1 transgene had no effect on the length of female GHR-KO or GHR-N mice. (C) Measurement of body weight after extensive removal of adipose tissue documented that the RIP::IGF-1 transgene did not affect the combined weight of nonadipose tissues. (D) Biochemical analysis of plasma IGF-1 concentration confirmed that the RIP::IGF-1 transgene did not increase the circulating levels of IGF-1 in GHR-KO mutants or GHR-N littermates. (E) Adipose depot weight analysis showed no effect of the RIP::IGF-1 transgene on the weights of the following depots (relative to whole body weight): perigonadal, perinephric, retroperitoneal, mesenteric, total visceral adipose tissue (VAT), subcutaneous white adipose tissue (SWAT), brown adipose tissue (BAT), total subcutaneous adiposity, or total adiposity. All measures of central tendency are arithmetic means, and all depictions of variation (error bars) represent standard deviations (SD). (See also Figs S1–S2).

Related to the specificity of the local expression of the transgene, we observed that there was no effect of the RIP::IGF-1 transgene on the following parameters of proliferative potential: body weight at any age (up to 17 months) (Fig.1A), body weight gain (up to 17 months) (Fig.1A), body length at approximately 12 months (Fig.1B), lean body weight (Fig.1C), and plasma IGF-1 concentration (Fig.1D). Also, there was no effect of the RIP::IGF-1 transgene on the absolute or relative weights of the following organs: brain, liver, lungs, heart, kidneys, spleen, thymus, gastrocnemii, ovaries, testes, or (importantly) hypothalamus (Gannon *et al*., 2000; Gerozissis, 2003; Choudhury *et al*., 2005; Nguyen *et al*., 2006; Choi *et al*., 2008) (data not shown); nor on adipose depot weights [perigonadal, perinephric, retroperitoneal, mesenteric, total visceral adipose tissue (VAT), subcutaneous white adipose tissue (SWAT), brown adipose tissue (BAT), total subcutaneous adiposity, or total adiposity] (Fig.1E). [We did, however, observe an age/aging-dependent increase in pancreatic weight attributable to the RIP::IGF-1 transgene in middle-aged (approximately 25-month-old) female GHR-KO and GHR-N mice (Fig. S1)]. There was also no difference in 20 blood cell population (erythrocytes, leukocytes, and platelets) parameters, as assessed by complete blood cell count analysis (data not shown) [although there were some provocative differences due exclusively to the *Ghr/bp* gene disruption (Fig. S2)]. Moreover, an assessment of pathology of 13-month-old female mice showed no transgene-induced increase in tumor burden or inflammation (data not shown). The significance of this absence of differential systemic IGF-1 levels or actions (i.e. inducing cellular or histological proliferation, or increasing tissue size), among mice with the RIP::IGF-1 transgene, is that it strongly suggests that the expression and actions of the transgene are either absent or biologically not meaningful outside of the insulin-producing β-cells of the pancreatic islets. Yet, the potential for pleiotrophic effects of changes in insulin levels or ectopic effects of *Igf-1* expression beyond our analyses, such as heightened *Igf-1* mis-expression in the central nervous system that affects peripheral insulin sensitivity, cannot be ruled out based on the data we collected.

### Evidence of insulin-mediated normalization of insulin sensitivity in GHR-KO;RIP::IGF-1 mice

**Figure 2:**
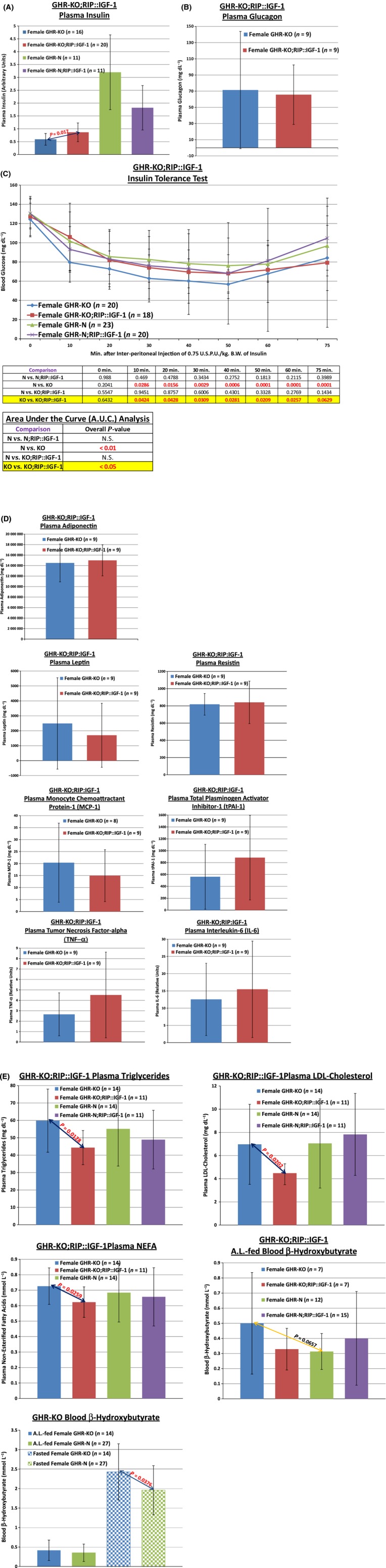
The RIP::IGF-1 Transgene Normalizes the Slow-Aging-Associated Insulin Sensitivity of the GHR-KO Mouse Specifically by Increasing Blood Insulin Concentration. (A) Plasma insulin concentration determination exhibited RIP::IGF-1 transgene-mediated increased insulin content in GHR-KO females. (B) Plasma glucagon concentration measurement showed no effect of the RIP::IGF-1 transgene on glucagon content. (C) Insulin tolerance testing (with statistical analysis table) revealed that female GHR-KO mice are more insulin-sensitive than their GHR-N littermate controls, but that GHR-KO;RIP::IGF-1 mice have the same degree of sensitivity to insulin as GHR-N mice. (D) Analysis of the plasma levels of the following proteins revealed no effect of the RIP::IGF-1 transgene in GHR-KO mice: adiponectin, leptin, resistin, monocyte chemo-attractant protein 1 (MCP-1), plasminogen activator inhibitor 1 (PAI-1), tumor necrosis factor-alpha (TNF-α), and interleukin-6 (IL-6). (E) Assessment of the concentrations of the following lipid-associated energetic constituents documented the following RIP::IGF-1-mediated reductions in female GHR-KO mice: plasma triglycerides, plasma low-density lipoprotein (LDL)-cholesterol, and plasma nonesterified fatty acids (NEFAs); although there was no statistically significant outcome of the transgene on AL-fed or fasted blood β-hydroxybutyrate, fasted blood β-hydroxybutyrate concentration was greater in GHR-KO mice than in GHR-N controls (analysis grouped all GHR-KO mice together and all GHR-N mice together, regardless of the presence of the RIP::IGF-1 transgene, as the central tendency ± dispersion values within *Ghr/bp* genotype were very similar). All measures of central tendency are arithmetic means, and all depictions of variation (error bars) represent standard deviations (SD). (See also Fig. S3.).

In concurrence with previously published results on GHR-KO;RIP::IGF-1 mice that showed that β-cell overexpression of *Igf-1* in GHR-KO mice restored islet cell mass through cell hyperplasia and hypertrophy (Guo *et al*., 2005), the transgene increased the concentration of insulin in the blood of female GHR-KO;RIP::IGF-1 mice (*P* = 0.017) (Fig.2A, data pooled from two independent trials). Importantly, it did not alter the levels of glucagon in the blood (Fig.2B); as glucagon is produced within the α-cells of the pancreatic islets, this further corroborates the acute specificity of the exogenous *Igf-1*'s β-cell-expression enrichment. Consistent with previously published data on heightened glucose-stimulated insulin secretion (GSIS) in GHR-KO;RIP::IGF-1 mice (Guo *et al*., 2005), and with the increased levels of circulating insulin in GHR-KO mice carrying the RIP::IGF-1 transgene (Fig.2A), comparisons of HOMA-IR and HOMA-β scores suggested increased insulin resistance without β-cell dysfunction, respectively, in female GHR-KO;RIP::IGF-1 mice relative to control GHR-KO mice (data not shown). As it is possible to be misled by such calculations (Boparai *et al*., 2010), we tested this *sine qua non* of our study more rigorously by insulin tolerance testing these mice. The RIP::IGF-1 transgene attenuates the insulin sensitivity of the female GHR-KO mouse to a degree that makes it indistinguishable from littermate controls (*P* = 0.1434) (Fig.2C, combined data from three independent trials). [No transgene-associated differences in body weight manifested for body weights measured immediately preceding these experiments (data not shown)]. Further, the RIP::IGF-1 transgene did not affect the circulating concentration of any of the following insulin sensitivity-related adipocytokines: adiponectin, leptin, resistin, monocyte chemo-attractant protein 1 (MCP-1), plasminogen activator inhibitor 1 (PAI-1), tumor necrosis factor-alpha (TNF-α), or interleukin-6 (IL-6) (Fig.2D). As triacylglycerides and other lipid constituents negatively affect insulin sensitivity, we investigated whether the insulin-desensitizing effect of the RIP::IGF-1 transgene could be due to an induction in those macromolecules. In actuality, we documented RIP::IGF-1-mediated *reduction* of plasma triglycerides (*P* = 0.0128), plasma low-density lipoprotein (LDL)-cholesterol (*P* = 0.0202), and plasma nonesterified fatty acids (NEFAs) (*P* = 0.0259) (Fig.2E). [The transgene did not affect plasma high-density lipoprotein cholesterol (HDL-cholesterol) or plasma total cholesterol levels (data not shown); blood β-hydroxybutyrate concentration, which exhibits a greater degree of induction in female GHR-KO mice than in their littermate controls (*P* = 0.0376) (Fig.2E), was not affected by the RIP::IGF-1 transgene to a statistically significant extent (Fig.2E)]. Counter-intuitively, effects on lipid energetics suggestive of poorer health prognostics appeared to be mediated solely by the *Ghr/bp* gene disruption in males (higher triglycerides and NEFAs, as well as lower HDL-cholesterol) (Fig. S3).

Thusly, we established that the RIP::IGF-1 transgene specifically reduces insulin sensitivity by increasing circulating insulin levels.

### Endocrinological outcomes of RIP::IGF-1 transgene

Regulation of blood glucose concentration is a vital part of the homeostatic control of neuroendocrinological and neurobiological function that declines with aging (Dominguez *et al*., 2010). The consequences of failures in this regulatory capacity are evident in the epidemic rates of type II diabetes mellitus (Centers for Disease Control & Prevention, 2011, 2012), especially in older/elderly populations (Greene, 1986; Dominguez *et al*., 2010; Viljoen & Sinclair, 2011; Gong & Muzumdar, 2012), and the microvascular damage attributed to chronic hyperglycemia (Centers for Disease Control & Prevention, 2011, 2012).

We examined the effect of the RIP::IGF-1 transgene on varied endocrinological measures that are correlated with gerontological phenotypes. Prior investigation had shown that expression of RIP::IGF-1 specifically ameliorated the diminished islet cell growth (insofar as cell size and population) in GHR-KO mice, thus showing that IGF-1 signaling (independent of GH signaling) can mediate islet cell growth, inhibition of apoptosis, and regulation of insulin biosynthesis and secretion (Liu *et al*., 2004; Guo *et al*., 2005). Furthermore, these studies showed normalization of glucose tolerance and first-phase glucose-stimulated insulin secretion (GSIS) in GHR-KO mice by introduction of the RIP::IGF-1 transgene, as well as enhanced second-phase GSIS in the control mice (Guo *et al*., 2005). All experiments henceforth were performed in females for two reasons: (i) the normalizing effects of the RIP::IGF-1 transgene on insulin sensitivity were more robust in female GHR-KO mice, and (ii) GHR-KO stock male littermate controls are insensitive to a common dosage of insulin (0.75 USPU per kg BW) during insulin tolerance testing (Bonkowski *et al*., 2006; Arum *et al*., 2009, data not shown), thusly confounding any assessments of the effects of the RIP::IGF-1 transgene on them (as we do not desire to investigate the slow-aging-associated effects of inducing insulin resistance, merely those consequent to normalizing insulin sensitivity).

Once again, the RIP::IGF-1 transgene had no effect on body weight trajectories (Fig. S4), and the same was true for absolute and relative organ weights of multiple organs listed previously (data not shown). Moreover, more discriminating analyses corroborated this lack of growth effect by showing no effect of the RIP::IGF-1 transgene on body weight change (in grams or in percentage) (Fig. S5).

Supporting our hypothesis, we observed robust normalizing effects of the RIP::IGF-1 transgene on multiple parameters of blood glucose homeostatic control in GHR-KO mice. Basal and fasted blood glucose concentration, *ad libitum*-fed (basal) and fasted glucose tolerance (as measures of GSIS-mediated insulin action), insulin sensitivity over a 20-fold dose range of insulin boluses, and pyruvate conversion performance (for testing gluconeogenic potential) were assessed.

#### Glucose bolus assimilation

**Figure 3:**
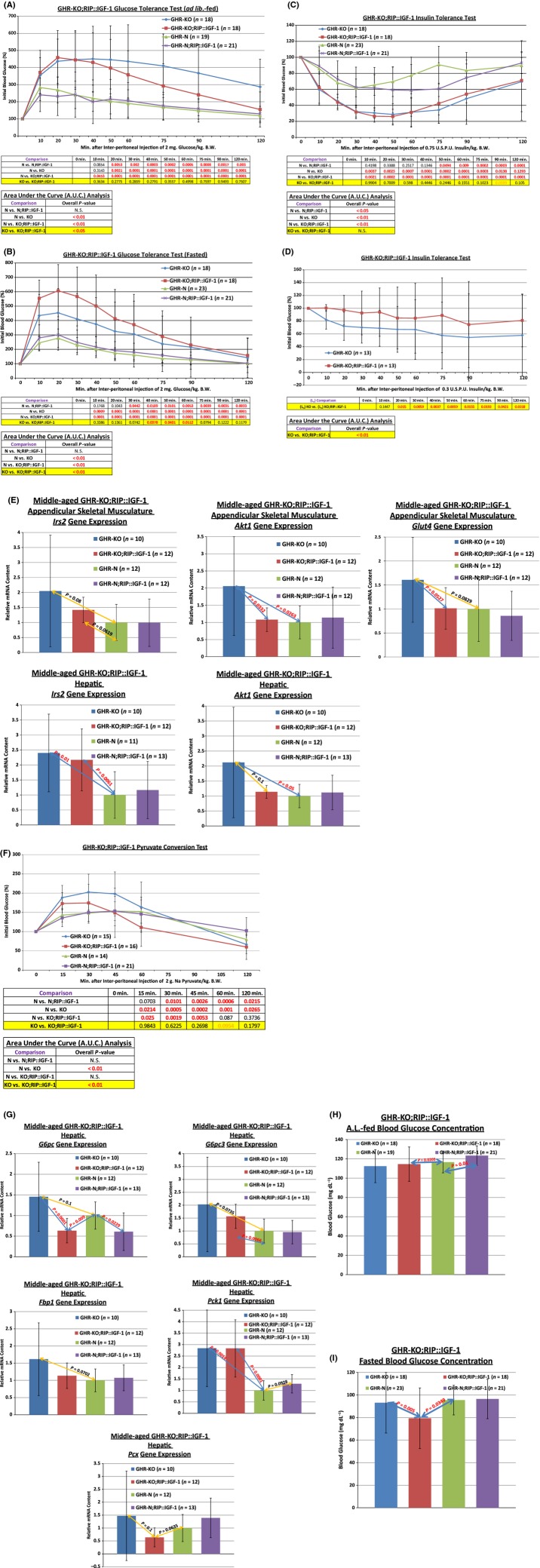
The RIP::IGF-1 Transgene Normalizes the Slow-Aging-Associated Endocrinological Phenotypes of the Female GHR-KO Mouse. (A) AL-fed glucose tolerance testing (normalized values, with statistical analysis table). (B) Fasted glucose tolerance testing (normalized values, with statistical analysis table). (C) 0.75 USPU insulin tolerance testing (normalized values, with statistical analysis table). (D) 0.3 USPU insulin tolerance testing (normalized values, with statistical analysis table). (E) Gene expression analysis of insulin sensitivity-mediating genes in appendicular skeletal musculature and hepatic tissues. (F) Pyruvate conversion testing (normalized values, with statistical analysis table). (G) Gene expression analysis of hepatic gluconeogenesis-modulating genes in hepatic tissues. (H) AL-fed blood glucose concentration testing (with statistical analysis table). (I) Fasted blood glucose concentration testing (with statistical analysis table). All measures of central tendency are arithmetic means, and all depictions of variation (error bars) represent standard deviations (SD). (See also Figs S4–S15).

AL-fed glucose tolerance testing (GTT) using a 2 g glucose kg^−1^ body weight (BW) concentration confirmed the previously documented poorer glucose tolerance of GHR-KO mice relative to their normal littermates [*P* = 0.0021, (Fig.3A); Coschigano *et al*., 1999; Guo *et al*., 2005], which is consistent with decreased glucose-stimulated insulin secretory ability of the β-cells of GHR-KO mice. The RIP::IGF-1 transgene improved the glucose tolerance of the female GHR-KO mouse (*P* < 0.05, Fig.3A, with absolute blood glucose value-based plots in Fig. S6). There was no effect of the RIP::IGF-1 transgene on BW as measured immediately preceding the testing (Fig. S7).

Fasted GTT (utilizing a 2 g glucose kg^−1^ BW concentration after a fast of approximately 16 h) confirmed the previously documented inferior glucose tolerance of GHR-KO mice relative to their GHR-N littermates (*P* = 0.0009, Fig.3B), consistent with decreased GSIS activity of the β-cell-deficient GHR-KO mice. During those fasting conditions, not only did the transgene not improve blood glucose incorporation in female GHR-KO mice, it *worsened* it (*P* = 0.0512, Fig.3B, with absolute blood glucose value-based plots in Fig. S8). Note that here also there was no effect of the transgene on BW as measured immediately before the testing (Fig. S9).

#### Insulin sensitivity

Suppression of glucose levels in ITT using 0.75 United States Pharmacopeia Units (USPU) per kg BW did not differ between GHR-KO and GHR-KO;RIP::IGF-1 females (Fig.3C, with absolute blood glucose value-based plots in Fig. S10). Results similar to those obtained with the 0.75 USPU per kg BW dosage were obtained with a 0.6 USPU per kg BW dose (data not shown). Suspecting a ‘floor effect’, we employed lower doses of insulin for subsequent ITT experiments.

0.3 USPU kg^−1^ BW ITT'ing revealed the normalization of insulin sensitivity by the RIP::IGF-1 transgene in female GHR-KO mice (*P* = 0.0506, Fig.3D, with absolute blood glucose value-based plots in Fig. S12) documented in preliminary analyses (Fig.2C). Similar results were achieved with a 0.0375 USPU kg^−1^ BW insulin dosage (data not shown).

There was no effect of the transgene on BW of subjects immediately prior to any of the ITT'ing (Figs S11 and 13).

In order to elucidate this normalization of the insulin sensitivity of the GHR-KO mouse by the RIP::IGF-1 transgene, we investigated some of the macromolecules active in insulin-mediated glucose uptake [*insulin receptor substrate 2* (*Irs2*), *thymoma viral proto-oncogene 1* (*Akt1/PKB*), and *solute carrier family 2 (facilitated glucose transporter), member 4* (*Slc2a4/Glut4*)] in insulin-responsive tissues [the liver and the appendicular skeletal muscle (specifically, the gastrocnemius)]. These gene expression analyses yielded that *Irs2* (*P* = 0.08), *Akt1* (*P* = 0.0263), and *Glut4* (*P* = 0.0829) were increased in GHR-KO appendicular skeletal muscle and that *Irs2* (*P* = 0.01) and *Akt1* (*P* = 0.05) were increased in GHR-KO liver (Fig.3E). For muscular *Akt1*, the RIP::IGF-1 transgene in GHR-KO mice reduced the transcript levels (*P* = 0.0332) to those indistinguishable from GHR-N controls (*P* = 0.6479) (Fig.3E). For muscular *Irs2*, the RIP::IGF-1 transgene in GHR-KO mice reduced the transcript levels (*P* = 0.0527) to those indistinguishable from GHR-N controls (*P* = 0.9556) (Fig.3E). Additionally, for hepatic *Akt1*, the RIP::IGF-1 transgene in GHR-KO mice reduced the transcript levels (*P* = 0.1) to those indistinguishable from GHR-N controls (*P* = 0.284) (Fig.3E).

#### Gluconeogenesis

Pyruvate conversion testing showed that GHR-KO females produced more glucose than their normal littermate controls (*P* = 0.0265, Fig.3F), consistent with hypoinsulinemia of GHR-KO mice, and that the RIP::IGF-1 transgene partially normalized this heightened gluconeogenic potential (when glucose production after 60 or 120 min is considered) (Fig.3F, with absolute blood glucose value-based plots in Fig. S14), consistent with the partial normalization of blood insulin content in GHR-KO;RIP::IGF-1 relative to GHR-KO mice (Fig.2A). As with the other blood glucose regulatory dynamics tests, there was no effect of the transgene on body weight as measured immediately preceding the testing (Fig. S15).

Endeavoring to connect the results from the pyruvate conversion test more tenably to *de novo* hepatic glucose production (HGP), we investigated the hepatic mRNA content of several genes involved in gluconeogenesis: *glucose-6-phosphatase, catalytic* (*G6pc*), *glucose 6 phosphatase, catalytic, 3* (*G6pc3*), *fructose bisphosphatase 1* (*Fbp1*), *phosphoenolpyruvate carboxykinase 1, cytosolic* (*Pck1/PEPCK*), and *pyruvate carboxylase* (*Pcx/Pc*). These analyses showed that *G6pc* (*P* = 0.1), *G6pc3* (*P* = 0.0735), *Fbp1* (*P* = 0.0702), and *Pck1* (*P* = 0.0014) transcript concentrations were increased in the livers of GHR-KO mice relative to their GHR-N littermates (Fig.3G). For *G6pc*, the RIP::IGF-1 transgene in GHR-KO mice reduced transcript content (*P* = 0.0001) even *below* the level of the littermate control mice (*P* = 0.009) (Fig.3G); the same effect was observed for *Pcx*, for which the RIP::IGF-1 transgene in GHR-KO mice reduced the transcript content (*P* = 0.1) to a level below that of the controls (*P* = 0.0635) (Fig.3G).

#### Unstimulated blood glucose

Measurements of blood glucose concentrations revealed that the RIP::IGF-1 transgene failed to reduce the blood glucose concentration of female GHR-KO mice during AL-fed (Fig.3H), although it did during fasted (Fig.3I), conditions.

In sum, expression of the RIP::IGF-1 transgene partially corrected glucose intolerance, rescued insulin sensitivity, partially ameliorated the aberrantly heightened gluconeogenic production, and reduced fasted blood glucose content in female GHR-KO mice.

### Partial normalization of various metabolic phenotypes of GHR-KO;RIP::IGF-1 mice concurs with effects on blood glucose homeostatic control

Neither metabolic efficiency (food consumed per weight gained) nor feed efficiency (weight gained per food consumed) was altered by the RIP::IGF-1 transgene in AL-fed GHR-KO females (Figs S16 and S17).

#### Indirect calorimetry

Previous indirect calorimetric measurements of metabolism increased (Westbrook *et al*., 2009) as well as decreased (Mookerjee *et al*., 2010; Carrillo & Flouris, 2011) metabolism in animals with extended longevity.

In primary analysis, we generally observed that the oxygen consumption (VO_2_), respiratory quotient (RQ)/respiratory exchange ratio (RER), heat production (Cal. per hour), and energy expenditure (EE) in AL-fed and fasted female GHR-KO;RIP::IGF-1 mice were partially or completely normalized from the relatively divergent levels at which they have previously been shown at for GHR-KO mice (Westbrook *et al*., 2009) (Figs S18–S22).

No genotype- or diet-based differences were detected for food consumption, changes in body weight induced by either acclimation or fasting, or thermogenesis (as crudely measured with an ambient thermometer in each of the chambers), while the subjects were in the indirect calorimetry chambers from the acclimation day to the AL-fed day to the fasted day (data not shown).

Analysis of the results from our repetition of the primary experiments of AL-fed indirect calorimetric data over the entire course of a day revealed the partial or full normalization of oxygen consumption (*P* = 0.0001), carbon dioxide output (*P* = 0.0008), respiratory quotient (*P* = 0.0001), heat production (*P* = 0.0001), and energy expenditure (*P* = 0.0001) by the presence of the RIP::IGF-1 transgene within female GHR-KO mice (Table1).

**Table 1:** Indirect calorimetry-based assessment of total *ad libitum*-fed metabolism

Arithmetic mean	VO_2_	VCO_2_	R.Q. (R.E.R.)	Heat Prod.	Energy Exp.
N on A.L. (*n* = 12)	*46.0284*	*39.6722*	*0.8636*	*385.5301*	*948.1727*
KO on A.L. (*n* = 15)	*91.6681*	*56.0253*	*0.6533*	*412.9019*	*1772.5304*
N on C.R. (*n* = 7)	*54.6331*	*51.8225*	*0.8973*	*434.147*	*1149.1911*
KO on C.R. (*n* = 17)	*94.9652*	*59.5818*	*0.7117*	*375.2352*	*1843.8404*
Comparison	VO_2_	VCO_2_	R.Q. (R.E.R.)	Heat Prod.	Energy Exp.
N on A.L. versus KO on A.L.	**0.0001**	**0.0001**	**0.0001**	0.194	**0.0001**
N on A.L. versus N on C.R.	0.0262	**0.0001**	0.0601	0.0966	**0.0027**
N on A.L. versus KO on C.R.	**0.0001**	**0.0001**	**0.0001**	0.6373	**0.0001**
KO on A.L. versus KO on C.R.	0.4652	0.0139	**0.0026**	0.0248	0.3517

Analyses of indirect calorimetry data obtained during fasting conditions revealed the same effects as those for AL-fed animals, with the effects being even more pronounced (Table S3c).

Although the calculations of mean values differed slightly, the results of analyses for the phase of day during which the subjects were more active (the dark phase from the 19:00 hour to the 07:00 hour) did not differ from those of the whole-day analysis (Table S3a and d). The same applied to the results obtained during the less active section of the subjects’ day (the lighted phase from the 08:00 hour to the 18:00 hour) (Table S3b and e).

#### Body core's temperature

Decreased body temperature has been associated with increased survival in humans (Roth *et al*., 2002), documented in short- and long-term calorically restricted *Macaca mulatta* (Lane *et al*., 1996), and experimentally shown to induce longevity in mice (Conti *et al*., 2006).

**Figure 4:**
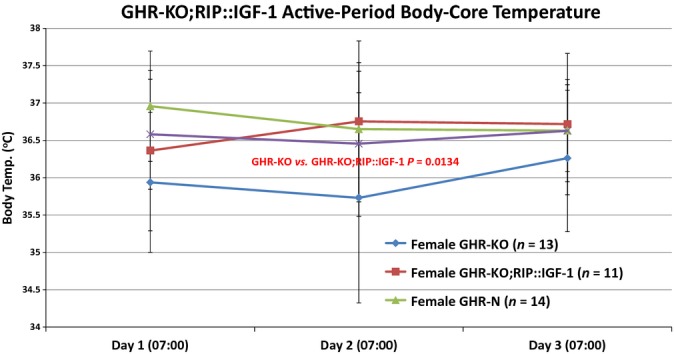
The RIP::IGF-1 Transgene Normalizes the Slow-Aging-Associated Body-Core Temperature of the Female GHR-KO Mouse. Body-core temperature measured at the end of the more active period (darkened) half of the subjects’ day confirmed that female GHR-KO mice consistently have a lower body-core temperature than their littermate controls and established that female GHR-KO;RIP::IGF-1 mice are indistinguishable from female GHR-N mice in this respect. All measures of central tendency are arithmetic means, and all depictions of variation (error bars) represent standard deviations (SD). (See also Figs S16–S22, and Table S4a–e).

Importantly, we observed that the RIP::IGF-1 transgene partially normalized the body core's innately low temperature in female GHR-KO mice during the more active (darkened) period of their day (*P* = 0.0134, Fig.4); similar, albeit not statistically significant, trends were detected during the less active (lighted) period of the day (data not shown).

### RIP::IGF-1 transgene partially normalizes behavioral and cognitive phenotypes

We noted no effect of the RIP::IGF-1 transgene on behavioral metrics assessed in the employed five-point functional observation battery (Table S1) (data not shown).

#### Spontaneous locomotion

As we did not detect any effect of the *Ghr/bp* gene disruption on the examined spontaneous locomotor activity variables, we could not scrutinize a normalizing effect of the RIP::IGF-1 transgene (Table2). Instead, we observed a tentative attenuating effect on total distance traveled by female GHR-KO mice as induced by the RIP::IGF-1 transgene (*P* = 0.0176); this distance traveled was so greatly reduced by the transgene that female GHR-KO;RIP::IGF-1 mice differed robustly from GHR-N littermates (*P* = 0.0007) (Table2).

**Table 2:** Total *ad libitum*-fed voluntary locomotor activity

Arithmetic mean	Total distance	Hor. activity Ct.	Amb. activity Ct.	Rest time	Rest episode Ct.	Mvmt. time	Mvmt. episode Ct.	Stereo. time	Stereo. episode Ct.	Stereo. activity Ct.	Vert. episode Ct.	Vert. activity Ct.	Vert. activity time	Loco. clock. revs.	Loco. counter. revs.
N on A.L. (*n* = 12)	*56.9752*	*204.6205*	*171.0504*	*554.4677*	*41.1762*	*45.4566*	*40.4714*	*9.177*	*13.7315*	*33.5702*	*8.971*	*25.8674*	*11.2301*	*0.2286*	*0.2367*
KO on A.L. (*n* = 15)	*56.3851*	*192.6097*	*163.1155*	*556.6244*	*38.1217*	*43.3094*	*37.3903*	*7.9026*	*12.5841*	*29.4942*	*3.8304*	*6.8155*	*2.5093*	*0.2681*	*0.229*
N on C.R. (*n* = 7)	*68.7457*	*231.3768*	*191.912*	*550.7739*	*42.2298*	*49.1525*	*41.528*	*10.6875*	*15.7495*	*39.4648*	*9.2143*	*25.2443*	*9.9293*	*0.2557*	*0.3302*
KO on C.R. (*n* = 17)	*64.2217*	*216.6341*	*182.1955*	*553.7476*	*41.5477*	*46.1707*	*40.8561*	*8.9517*	*14.3607*	*34.4386*	*7.1078*	*6.7298*	*15.9952*	*0.3376*	*0.2122*
Comparison	Total distance	Hor. activity Ct.	Amb. activity Ct.	Rest time	Rest episode Ct.	Mvmt. time	Mvmt. episode Ct.	Stereo. time	Stereo. episode Ct.	Stereo. activity Ct.	Vert. episode Ct.	Vert. activity Ct.	Vert. activity time	Loco. clock. revs.	Loco. counter. revs.
N on A.L. versus KO on A.L.	0.9371	0.5322	0.6443	0.5747	0.1661	0.576	0.1659	0.0299	0.2379	0.0867				0.4116	0.8377
N on A.L. versus N on C.R.	0.1801	0.2782	0.3389	0.4354	0.724	0.4345	0.7255	0.0603	0.1187	0.0688	0.8449	0.8843	0.4361	0.5075	0.094
N on A.L. versus KO on C.R.	0.3753	0.5288	0.5084	0.837	0.8632	0.8381	0.8595	0.7026	0.5453	0.7347				0.1661	0.3991
KO on A.L. versus KO on C.R.	0.3584	0.211	0.2654	0.4124	0.0766	0.4144	0.0756	0.0496	0.0743	0.0396	**0.0002**	0.923	**0.0001**	0.3678	0.6131

As was the case for the results of indirect calorimetry-based metabolic assessment, the spontaneous locomotor activity during the phase of day in which the subjects were more active (the dark phase from the 19:00 hour to the 07:00 hour) (Table S4a) or during the less active section of the subjects’ day (the lighted phase from the 08:00 hour to the 18:00 hour) (Table S4b) did not differ from those of the complete analysis.

Additionally, the conclusions drawn from analyses of AL-fed day measures largely concurred with those of fasted day measures (Table2 and S4c–e). Unexpectedly, when transitioning from AL-fed to fasting conditions, a decrease in voluntary movement was observed in female GHR-KO mice, yet not in their littermate controls (Tables2 and S4c).

#### Anxiety

**Figure 5:**
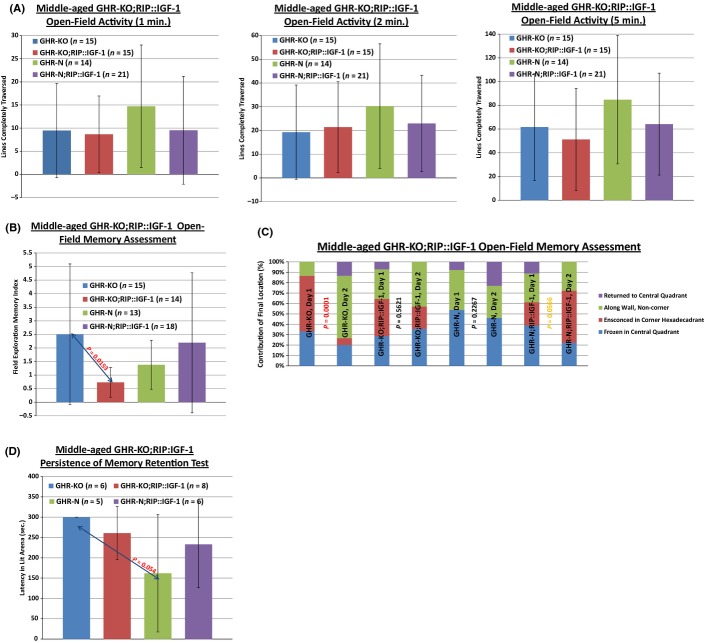
The RIP::IGF-1 Transgene Normalizes the Slow-Aging-Associated Long-Term Memory Retention Capability of the Middle-aged Female GHR-KO Mouse. (A) Open-field assessment of anxiety detailed no effect of the RIP::IGF-1 transgene on anxiety, whether anxiety is defined as exploratory locomotion measured over 1, 2, or 5 min. (B) Open-field testing paradigm-based assessment of proximal (24-h delay) long-term memory performance documented greater memory capability in the middle-aged female GHR-KO mice relative to their GHR-N littermate controls, yet an invariant degree of capability between GHR-KO;RIP::IGF-1 mice and GHR-N controls. (C) Open-field testing paradigm-based assessment of proximal (24-h delay) long-term memory performance exhibited a great alteration of the proportional distribution of final location in middle-aged female GHR-KO mice, which was not documented in GHR-N littermate controls or GHR-KO;RIP::IGF-1 mice. (D) Passive avoidance chamber paradigm-based assessment of distal [(50 + 1)-day delay] long-term memory performance displayed that female GHR-KO;RIP::IGF-1 mice exhibit greater persistence of memory than GHR-KO mice, as considered by retention testing latency. All measures of central tendency are arithmetic means, and all depictions of variation (error bars) represent standard deviations (SD). (See also Figs S23–S24, and Table S5a–e.).

Anxiety examination revealed no effect of the RIP::IGF-1 transgene for this behavioral measure (Fig.5A). Additionally, we did not note any basal anxiety during home-cage functional observation assessment (data not shown). These results suggest that the possible expression of RIP::IGF-1 in the hypothalamus (Gannon *et al*., 2000; Gerozissis, 2003; Choudhury *et al*., 2005; Nguyen *et al*., 2006; Choi *et al*., 2008) did not influence the hypothalamic–pituitary–adrenal (H→P→A) axis activity. Combined with the previously mentioned lack of effect on weight or size of the hypothalamus, and the lack of an effect of the transgene on stereotypy time, stereotypy episode count, or stereotypy activity count during behavioral analyses of spontaneous locomotion (Tables2 and S4a–e), we conclude that any possible ectopic *Igf-1* expression induced by the RIP::IGF-1 transgene within the hypothalamus is physiologically inconsequential for this study.

#### Memory

The retention of cognitive capability (in particular, memory function) into advanced middle age in the GHR-KO mice indicates that the effects of the *Ghr/bp* disruption extend beyond increasing survivorship, to include ameliorating senescence and the resultant functional decrements (Kinney *et al*., 2001a,b2001b; Kinney-Forshee *et al*., 2004; Bartke, 2005a). To test our hypothesis, we investigated the effect of the RIP::IGF-1 transgene on the memory function of female middle-aged mice.

Within a proximal long-term memory testing structure, in which memory for an open-field context was tested 24 h after initial exposure, we found that the RIP::IGF-1 transgene normalizes memory performance of the middle-aged female GHR-KO mouse back to the level of the GHR-N littermate control. A comparison of the memory index [retention testing performance (number of square lines fully crossed on day 2)/acquisition testing performance (number of square lines fully crossed on day 1)] corroborated that GHR-KO mice have a higher memory index than their GHR-N littermate controls, while revealing that GHR-KO;RIP::IGF-1 mice have a memory index indistinguishable from that of those controls (Fig.5B).

As an indicator maximal performance, we also considered how well the subjects were performing (i.e. remembering previous experience within the context and thus being more inclined to explore) at the end of the 60-s testing period. Our further examination of the location that a subject ultimately reached after 60 s in the chamber (percent contribution of final location) corroborated the memory index results by showing that GHR-KO mice improve exploratory performance from day 1 to day 2 (*P* = 0.0001), indicative of enhanced memory of presence in the context 24 h prior, but that their littermates do not and that GHR-KO;RIP::IGF-1 mice also do not improve in this respect (Fig.5C).

Although there was no difference in distal long-term memory testing (per passive avoidance testing with a 50-day delay between acquisition and retention dates) (data not shown), we assessed the ability of the mice to retain the memory of that aversive stimulus (the 0.5 mA per 0.5 s foot-shock) 24 h after the retention test; this is analogous to the probe trials conducted in conjunction with Morris water maze tasking. This analysis of ‘persistence of memory retention’, based on mean raw values, revealed that GHR-KO mice outperformed their littermates (*P* = 0.054), but that GHR-KO;RIP::IGF-1 mice did not (Fig.5D).

### Putative connection between hypoinsulinemia/insulin sensitivity, FoxO3a transactivation, (chaperone-mediated) autophagy, and slowed senescence

At the interface of endocrinology and gerontology, the insulin receptor signaling pathway splits to ultimately participate in the regulation of multiple cellular effects. One major branch of this pathway extends through the Forkhead box class O 3A (FoxO3A) transcription factor. In multiple studies of unrelated human populations, *Foxo3a* variants have been associated with increased survivorship more extensively than polymorphisms for any other gene or locus (Willcox *et al*., 2008; Anselmi *et al*., 2009; Flachsbart *et al*., 2009; Li *et al*., 2009; Pawlikowska *et al*., 2009; Soerensen *et al*., 2010; Ziv & Hu, 2011; Däumer *et al*., 2014). (The predominance of these single nucleotide polymorphisms in noncoding regions is reviewed in Donlon *et al*., 2012; Flachsbart *et al*., 2013.) Insulin signaling regulates autophagy, the cellular/extra-cellular proteolytic process by which damaged proteins may be degraded and amino acids produced for *de novo* proteogenesis (Singh & Cuervo, 2011), and this has been suggested as a mechanism by which increased insulin sensitivity may be beneficial for increased survivorship and the abatement of senescence (Arum *et al*., 2009). Therefore, we conducted *in silico* analyses of the potential for FoxO3a transactivation of the two genes whose expression regulates chaperone-mediated autophagy, the *lysosome-associated membrane glycoprotein 2a* (*Lamp2a*) (Kaushik & Cuervo, 2012a), and the *heat-shock cognate* protein of approximately *73* kiloDaltons (*Hsc-73*) (Kaushik & Cuervo, 2012b). We discovered four canonical binding sequences for FoxO3a (5′-[AG]TAAA[TC]A-3′) within 10 kilobase pairs (kbps) upstream of *Lamp2a* (probability of chance occurrence = 3.55 × 10^−11^), and six such sequences upstream of *Hsc-73* (probability of chance occurrence = 2.12 × 10^−18^) (Fig. S25). While not as conclusive as chromatin immunoprecipitation or electrophorectic (gel) mobility shift assays, this information provides some insight into the mechanistic underpinnings of the association between hypoinsulinemia/insulin sensitivity, FoxO3a transactivation, autophagy-mediated protein and cellular turnover, and decelerated senescence.

## Discussion

Increased insulin sensitivity and efficient homeostatic control of blood glucose have been associated with extended survival and retention of good health and functionality in exceptionally long-lived mice and humans (centenarians and long-lived families) (Barbieri *et al*., 2001, 2003, 2009; DiStefano *et al*., 2007; Bartke, 2008; Barzilai & Bartke, 2009; Redman & Ravussin, 2009; Wijsman *et al*., 2011). Over the past fifteen years, the concept of an endocrinological component to the regulation of longevity has been substantiated by a considerable number of studies integrating endocrinology and gerontology. We have conducted many *associative* studies of this type with long-lived, somatotrophic signaling-defective mutant mice; we now progress to the first steps in testing the *necessity* for enhanced insulin sensitivity for the delayed senescence of these mice or the *sufficiency* of improved blood glucose homeostatic control for delayed aging in their normal counterparts.

In the present study, we found that ectopic pancreatic islet β-cell expression of IGF-1 in a GHR-KO mouse results in no differences in body characteristics related to growth or proliferative potential. Notably, the GHR-KO;RIP::IGF-1 double mutant has partially normalized plasma insulin concentration, improved blood glucose assimilation, normalized insulin sensitivity, suppressed gluconeogenic potential, and reduced fasted blood glucose concentration; yet no effect was detected in *ad libitum*-fed blood glucose levels, or plasma levels of glucagon, adiponectin, leptin, resistin, or several other macromolecules documented to affect insulin sensitivity. Furthermore, active period T_co_ was normalized, and indirect calorimetric measures of metabolism (such as respiratory quotient) were also normalized in double mutants. Further, constituents of lipid bio-energetic metabolism (triglycerides, NEFAs, and LDL-cholesterol) are normalized in GHR-KO;RIP::IGF-1 mice. We uncovered that spontaneous locomotion was decreased in GHR-KO mice by the RIP::IGF-1 transgene. Finally, multiple incidences of clear normalization in the GHR-KO;RIP::IGF-1 double mutant of the cognitive retention phenotype of the middle-aged GHR-KO mouse, for both proximal and distal long-term memories, were discovered.

These results lead to the conclusion that the insulin sensitivity-suppressed GHR-KO;RIP::IGF-1 double mutant differs from the GHR-KO mouse in slow-aging-related parameters but in few, if any other, characteristics. This supports the hypothesis that enhanced insulin sensitivity is necessary for the retardation of senescence in the GHR-KO mouse.

The objective of this study was to test the hypothesis that the insulin sensitivity of the GHR-KO mouse is causal in the decreased rate of aging of this long-lived animal. Employing a twenty-fold range of insulin concentrations, we showed that the RIP::IGF-1 transgene normalizes the widely studied insulin sensitivity of slow-aging GHR-KO mice. Although there were other blood glucose regulation-related phenotypes engendered by the transgene (such as increased blood glucose assimilation after a glucose bolus and decreased glucose production after a pyruvate dosing), it is this normalization effect on insulin responsiveness that provided the basis for testing the potential effect of the transgene on other slow-aging-associated characteristics.

The major phenotypes induced by the RIP::IGF-1 transgene in GHR-KO mice were (i) an increase in insulinemia, (ii) a normalization of insulin sensitivity, (iii) a normalization of metabolic rate (e.g. increased respiratory quotient), (iv) a tentative decrease in spontaneous activity (i.e. total distance covered), and (v) a normalization of memory function. An additional, yet potentially related, effect was increased food consumption. We propose that the relative hyperinsulinemia-mediated normalization of insulin sensitivity was the most likely primary effect of the transgene, which led to the increase in metabolism [as heightened insulin levels increase metabolic rate (Acheson *et al*., 1983, 1984; Landsberg, 1986), and that the increased metabolic flux then forced increased energy consumption to meet its increased energetic flow. It is tempting to suggest that either this increased metabolism or the increased energy consumption, or a combination of the two, might have adversely affected memory function in middle-aged GHR-KO;RIP::IGF-1 mice (relative to their standard GHR-KO controls) {as hypermetabolism is associated with dementia in mice (Knight *et al*., 2012) and (albeit controversially) in humans (Poehlman & Dvorak, 1998).

The ostensibly detrimental (Blüher *et al*., 2003; Russell & Kahn, 2007; Muzumdar *et al*., 2008) yet potentially beneficial (Berryman *et al*., 2010; Masternak *et al*., 2012) effects of discrete adipose depots on aging and survival are intriguing areas of present gerontological investigation. Consistent with some of the previous findings (Berryman *et al*., 2004, 2010), we failed to observe any effect of the *Ghr/bp* gene disruption on the weight of discrete or total visceral adipose depots, although the mutation did increase subcutaneous adiposity (Fig.1E); identical results were obtained in late middle-aged (approximately 25-month-old) mice (data not shown). Furthermore, we detected no effect of the RIP::IGF-1 transgene, which appears to normalize many slow-aging-associated phenotypes of the GHR-KO mouse, on the weight of any adipose depots (Fig.1E). Thus, the decelerated senescence of GHR-KO mice can be modulated without gross alteration of adiposity.

Interestingly, late middle-aged (approximately 25-month-old) GHR-KO females have a greater BAT weight:total body weight ratio than their littermate controls (*P*-value < 0.0001, Fig. S26); this lends some evidence as to how GHR-KO mice, possessing an abnormally high surface area-to-body volume ratio, counteract their physical predilection to hypothermia.

GHR-KO;RIP::IGF-1 mice are capable of inducing a greater transcriptional (or possibly translational, but probably not simply secretory) insulin action response to hyperglycemia than standard GHR-KO mice when challenged with a glucose load under AL-fed conditions (Fig.3A). (The reduction in insulin sensitivity induced by the RIP::IGF-1 transgene might be partially responsible for the delayed response observed in GHR-KO;RIP::IGF-1 mice in the AL-fed GTT). Yet, the transgene not only does not engender such an effect under fasted conditions, it worsens glucose incorporation in GHR-KO mice (Fig.3B). It seems that GHR-KO;RIP::IGF-1 mice require *something* present during AL-fed situations [possibly simply the energy (i.e. nucleoside triphosphates) generated when not fasted, possibly macromolecules synthesized/retained only when food is available (e.g. fresh insulin signaling components that replace ones degraded, for energy and constituents, during fasting)] in order to initiate that response.

Exploration of the macromolecular regulators of the increased ability of GHR-KO mice to transduce a signal from an activated insulin receptor (INSR) hint that many components of the INSR→GLUT4 signaling pathway contribute to this ability. Consistent with the effects observed during the blood glucose regulatory dynamics assays, the RIP::IGF-1 transgene seems to inhibit these insulin sensitivity-related effects of the *Ghr/bp* gene disruption by means of multiple pathway components, namely appendicular skeletal muscular IRS2 and AKT1, and hepatic AKT1. These macromolecular results concur with the differences between ordinary GHR-KO mice and GHR-KO;RIP::IGF-1 mice in the glucose and insulin tolerance assessments.

Our transcriptional analysis of hepatic tissue suggests that the heightened hepatic glucose production capability of GHR-KO mice is mediated by numerous gluconeogenic proteins; further, we only detected evidence for G6PC and PCX being involved in the RIP::IGF-1 transgene's ability to blunt this enhanced gluconeogenic performance. Moreover, in addition to enriching the results of the pyruvate conversion test, based on this macromolecular data, we surmise that the difference in unstimulated fasted blood glucose concentration between regular GHR-KO mice and those bearing the RIP::IGF-1 transgene could be affected by HGP, in addition to changes in insulin sensitivity.

As has been often paraphrased within the last 10 years, understanding healthspan is arguably the most relevant clinical, social, and economic goal of aging research (Berryman *et al*., 2008; Kirkland & Peterson, 2009; Nass *et al*., 2009; Tatar, 2009; Bartke, 2011; Selman & Withers, 2011). We have previously documented that GHR-KO mice maintain youthful performance on memory tasks into middle age, while their littermate counterparts exhibit the expected, aging-associated decline in cognition (Kinney *et al*., 2001a). More recently, we have shown that aging-associated declines in motor coordination, grip strength, balance, and agility are delayed, attenuated, and/or prevented in aging GHR-KO mice (Arum *et al*., 2013) and that aging-associated declines in blood glucose regulatory homeostasis and dynamics are blunted or forestalled in aging GHR-KO mice (Arum *et al*., 2014). Future investigations of whether the attenuation of insulin sensitivity would negatively affects these healthspan features of GHR-KO mice, or if enhanced blood glucose homeostatic control could beneficially affect these healthspan characteristics in control mice, will be based on the present study on GHR-KO;RIP::IGF-1 mice.

Although hypoinsulinemia and enhanced insulin sensitivity have been linked with life extension in GH-related mutants and in dietarily restricted animals, a few long-lived mutants have been reported as not being insulin hypersensitive (Conover & Bale, 2007; Conover *et al*., 2008) or as being insulin resistant (Kurosu *et al*., 2005; Bartke, 2006), as have rapamycin-treated mice (Fang *et al*., 2013). An alternative hypothesis regarding the non-cell-autonomous importance of insulin action for health and survivorship posits that insulin resistance may be viewed in certain circumstances as a protective, or even beneficial, adaptation to aging- or diet-related increases in insulin levels (Barzilai *et al*., 2012). Thus, inferior or decreasing insulin sensitivity in older or elderly subjects could be construed as a ‘desensitizing’, not necessarily a ‘detrimental’, effect. It is certainly not the interpretation of this synthesis that insulin sensitivity is a nondissociable *sine qua non* of a decreased rate of aging; only that our review of the literature highlights that the two traits are positively correlated (Masternak *et al*., 2009), and our results presented herein suggest that the decelerated senescence of *some* mouse models might be dependent on their insulin sensitivity.

Lifespan was not assessed as part of our study. Future analyses of whether GHR-KO;RIP::IGF-1 mice live shorter than their standard GHR-KO counterparts, as the data that we have presented would suggest, are clearly required.

Other modulators of blood glucose regulatory efficiency worth probing in the context of this study include an alteration in pancreatic islet α-cell activity (e.g. the suppression of glucagon production/secretion by insulin). Very interesting new data are positioning the α-cells central in the development of types I and II diabetes mellitus (Unger & Orci, 2010; Habener & Stanojevic, 2012; Unger & Cherrington, 2012). As diabetic patients have been described as having ‘accelerated aging’ or ‘segmental progeroid phenotypes’ (Hamlin *et al*., 1975; Kent, 1976; Monickaraj *et al*., 2012), such study would be most germane to a consideration of the importance of blood glucose control to organismal senescence. Additionally, both hyperglucagonemia and insulin resistance may be seen in states of low body potassium (Chatterjee *et al*., 2011); such potentially important changes in electrolyte levels or proportions have not been investigated in any murine paradigm of longevity or delayed senescence.

Of special note to experimental gerontologists, we have characterized novel mice [littermate control mice harboring this transgene (GHR-N;RIP::IGF-1 mice)] that could be used for testing the necessity of insulin sensitivity for the healthspan or longevity of *any* long-lived, insulin-sensitive mouse. This should permit critical testing of hypotheses linking insulin and glucose homeostasis with healthspan and longevity. Multiple aging or aging-related studies, including some on human subjects, nontraditionally rely on glucose tolerance testing as a measure of insulin sensitivity, and show glucose tolerance as a correlate of longevity (Holzenberger *et al*., 2003; Holzenberger, 2004; Harper *et al*., 2005; Taguchi *et al*., 2007; Selman *et al*., 2009; Rozing *et al*., 2010; Fontana *et al*., 2010). Considering that GHR-N;RIP::IGF-1 outperform standard GHR-N mice in the assimilation of a bolus of glucose under AL-fed conditions, and that GHR-N;RIP::IGF-1 mice have lower hepatic gluconeogenic capability than GHR-N mice, it would be of interest to investigate differences in the onset or pace of senescence-associated features, possibly including survivorship, in these animals.

This study has potential translational significance, as many pharmacological agents affect insulin sensitivity [e.g. metformin (Glucophage) and thiazolidinediones (e.g. rosiglitazone or pioglitazone)] or blood glucose homeostasis control [e.g. actual macromolecules or analogs of insulin, glucagon, glucagon-like peptide 1 (GLP-1), incretins] might have beneficial effects on healthspan including microvascular and macrovascular complications of metabolic syndrome-related disorders, cognitive retention (as neurobiological function is dependent upon glucose utilization), or neuromusculoskeletal frailty and thus would seem to merit detailed examination of these potential effects.

## Experimental procedures

### Animal husbandry

*Growth hormone receptor/binding protein* gene-disrupted (GHR-KO) mice were generated by inserting a neomycin cassette replacing the 3′-end of the fourth exon and the 5′-end of intron 4/5 of the genomic sequence (Zhou *et al*., 1997). The founder population of GHR-KO mice was provided by Dr. John J. Kopchick (Ohio University, Athens, OH). RIP::IGF-1 mice were constructed by the introduction of a transgene with the rat insulin promoter 1 (RIP) driving the expression of the rat *insulin-like growth factor 1* (*Igf-1*) gene (Guo *et al*., 2005), and the founder population of RIP::IGF-1 mice was provided by Dr. Jun-Li Liu (McGill University, Montreal, Canada). GHR-KO, GHR-KO;RIP::IGF-1, GHR-N (heterozygous *littermate* controls for GHR-KO mice), and GHR-N;RIP::IGF-1 mice were generated by mating of GHR-KO males with females heterozygous for the *Ghr/bp*-disrupted allele (GHR-N) and also harboring the RIP::IGF-1 transgene (i.e. GHR-N;RIP::IGF-1 females). These breeding schemes produce *littermate* control mice that have the same genetic background and are subject to the same intrauterine and postnatal environment as the mutants.

Genotyping was conducted via quantitative polymerase chain reaction (q-PCR)-based technologies (Transnetyx, Inc., Cordova, TN, USA).

The resulting mice had elements of a 129/Ola, a Balb/c, two C57Bl/6J, and two C3HJ stocks; therefore, although lacking the methodological benefits of ‘reproducible genetic heterogeneity’ (Miller *et al*., 1999), this stock possesses considerable genetic variation, and thus, the results are likely applicable to other mouse populations.

The animals were maintained in shoebox-type cages in light- (12 h light to 12 h darkness) and temperature- (22 ± 2 °C) controlled rooms with constant access to Lab Diet Formula 5001 (23% protein, 4.5% fat, 6% fiber) (Nestle Purina, St. Louis, MO, USA) and tap water. Littermate control pups were weaned at the age of 21–23 days, and GHR-KO pups 2 weeks later or at the time of weaning normal pups from the next litter.

## Supporting Information

**Fig. S1** As noted in the Results section, there was no difference in the (raw or total body weight-normalized) weights of various internal organs as induced by the RIP::IGF-1 transgene in either young-adult female GHR-KO mice or their littermate controls. Yet, in an interaction of aging and genetic constituency, we did observe increased pancreatic weight caused by the RIP::IGF-1 transgene in *middle-aged* (approximately 25-month-old) GHR-KO females and their littermates, as well as the decreased pancreatic weight of middle-aged GHR-KO mice that was suggested by studies showing decreased β-cell weight in young-adult GHR-KO mice (Liu *et al*., 2004), which we display herein. We further display results on other differences in anatomical features that, to the best of our knowledge, are also aging-consequent effects for female GHR-KO mice; namely, the decreased normalized weights of the kidneys & liver (previously documented in 24-month-old GHR-KO females in Berryman *et al*., 2010), as well as the heart; and the increased normalized weights of the brain and gastrocnemii, as induced in middle age by the *Ghr/bp* gene disruption. [The increased body weight-normalized skeletal muscle weight was previously alluded to by the documentation of increased lean tissue weight in 24-month-old GHR-KO females (Berryman *et al*., 2010)]. **Fig. S2** Although there were no differences due to the RIP::IGF-1 transgene in the 18 blood cell parameters investigated, there were interesting differences due to the *Ghr/bp* gene disruption; which we present here. **Fig. S3** In addition to the RIP::IGF-1-engendered reductions in lipid energetics macromolecules (presented in Fig. 2E), there were also inductions due to the *Ghr/bp* gene disruption in male mice; which warrant presentation due to their running counter to the retention of health and the longevity of GHR-KO mice. **Figs S4 and S5** Corroborating and expanding upon the data in Fig. 1A, we present here further documentation of the crucial absence of an effect of the RIP::IGF-1 transgene on subject body weight; regardless of how or when the data is considered. **Figs S6–S15** For each repeated-measures blood glucose test presented in the main body of the report, body weight measurements taken immediately prior to testing are presented in the even-numbered supplemental figures between S7 and S15 (in an order corresponding to the order that the tests are presented in the main body). Also, corresponding absolute glucose concentration-based glucose excursion curves for each glucose tolerance, insulin tolerance, or pyruvate conversion test are presented in the odd-numbered supplemental figures between S6 and S14 (in an order corresponding to the order that the tests are presented in the main body). Particular emphasis is placed on the importance of there being no *Ghr/bp* genotype-related differences in testing-date body weight, as possibly induced by the RIP::IGF-1 transgene, for the blood glucose handling assays. This ensures that similar absolute amounts of glucose, insulin, or pyruvate were given to animals of the same *Ghr/bp* genotype. This is important not only because, at least for mice, smaller animals have been documented to be more insulin-sensitive, but also because it suggests that there was no substantial difference in body fat as caused by the transgene; which would have resulted in divergent lean musculature-to-body weight ratios and divergent pancreatic islet of Langerhans β-cell-to-body weight ratios that would have favored superior insulin-responsive glucose incorporation and greater glucose-stimulated insulin secretion, respectively, by the leaner mice. Thusly, during body weight-based, bolus-based testing, these anatomical alterations would affect blood glucose assimilation even if there were no other histological, cellular, or macromolecular differences. While differential blood glucose regulation by any means would be worth reporting and investigating further, a mechanistic understanding of the results presented herein is abetted by this lack of RIP::IGF-1 transgene-resultant effect on body weight. **Figs S16–S17** Longitudinal probing of the relations between food consumption and body weight gain yielded no differences in the amount of energy required to add a certain unit of weight (metabolic efficiency), nor the mathematical inverse (feed efficiency), as induced by the RIP::IGF-1 transgene. **Figs S18–S22** Preliminary investigation of indirect calorimetric measures of metabolism yielded RIP::IGF-1-consequent normalization of multiple parameters of heightened metabolic rate in GHR-KO mice. These data are corroborated by data from another cohort of subjects (detailed in Tables 1 and S3a–e). **Fig. S23** Descriptive pictures of the apparatus, and a model subject, utilized for anxiety and proximal long-term memory taxing (as presented in Figs 5A and 5B and C). Testing methodologies for both features are contained in the Supplemental Experimental Procedures section. **Fig. S24** Investigating the penetrance and expressivity of the effect observed in Fig. 5D, we present here the passive avoidance chamber paradigm-based assessment of distal [(50 + 1)-day delay] long-term memory performance; which showed that female GHR-KO;RIP::IGF-1 mice exhibit (statistically untestable) greater persistence of memory [as considered by the proportion of a population (penetrance) that displays full (300-s) latency on the ‘+1’ testing day (expressivity)] than GHR-KO mice. **Fig. S25** Data-mining of bioinformatics information related to chaperone-mediated autophagy (CMA) and the FoxO3A transcription factor unearthed multiple binding sequences/sites for FoxO3A upstream of the genomic sequences for the two genes that regulate CMA, *Lamp2a* and *Hsc-73*. The genomic sequence data was derived from the National Center for Biotechnology Information (NCBI) databases produced and maintained by The United States Department of Health and Human Services’ National Institutes of Health's National Library of Medicine (http://www.ncbi.nlm.nih.gov/). Due to the length of such information, it is presented here. **Fig. S26** Relevant to issues of thermoregulation, and the rate of metabolism, in GHR-KO mice, an aging-associated effect on brown adipose tissue weight effected by a combination of the *Ghr/bp* gene disruption and aging, is presented. **Table S1** Characteristics of interest in home-cage assessments are succinctly detailed. **Table S2a and b** Dependent Variables for Indirect Calorimetry Experiments (Table S2a) and Voluntary Locomotion xperiments (Table S2b) are briefly explained. **Table S3a–e** Sub-divisions of indirect calorimetric data analysis for the more-active and less-active periods of the AL-fed day recordings (Table S3a and b, respectively), and for the total, more-active, and less-active periods of the fasted day recordings (Table S3c–e, resp.) are presented here. **Table S4a–e.** Sub-divisions of voluntary locomotor activity data analysis for the more-active and less-active periods of the AL-fed day recordings (Table S4a and b, respectively), and for the total, more-active, and less-active periods of the fasted day recordings (Table S4c–e, resp.) are presented here. All measures of central tendency are arithmetic means, and all depictions of variation (error bars) represent standard deviations (SD).
