# Supplementary material for: Specific suppression of insulin sensitivity in growth hormone receptor gene-disrupted (GHR-KO) mice attenuates phenotypic features of slow aging
Source: Aging Cell. 2014 Sep 20;13(6):981–1000. doi: 10.1111/acel.12262 (PMC4326932; doi:10.1111/acel.12262)
Supplement: Supplementary file 1 — Fig. S1 As noted in the Results section, there was no difference in the (raw or total body weight-normalized) weights of various internal organs as induced by the RIP::IGF-1 transgene in either young-adult female GHR-KO mice or their littermate controls. Yet, in an interaction of aging and genetic constituency, we did observe increased pancreatic weight caused by the RIP::IGF-1 transgene in middle-aged (approximately 25-month-old) GHR-KO females and their littermates, as well as the decreased pancreatic weight of middle-aged GHR-KO mice that was suggested by studies showing decreased β-cell weight in young-adult GHR-KO mice (Liu et al., 2004), which we display herein. We further display results on other differences in anatomical features that, to the best of our knowledge, are also aging-consequent effects for female GHR-KO mice; namely, the decreased normalized weights of the kidneys & liver (previously documented in 24-month-old GHR-KO females in Berryman et al., 2010), as well as the heart; and the increased normalized weights of the brain and gastrocnemii, as induced in middle age by the Ghr/bp gene disruption. [The increased body weight-normalized skeletal muscle weight was previously alluded to by the documentation of increased lean tissue weight in 24-month-old GHR-KO females (Berryman et al., 2010)]. Fig. S2 Although there were no differences due to the RIP::IGF-1 transgene in the 18 blood cell parameters investigated, there were interesting differences due to the Ghr/bp gene disruption; which we present here. Fig. S3 In addition to the RIP::IGF-1-engendered reductions in lipid energetics macromolecules (presented in Fig. 2E), there were also inductions due to the Ghr/bp gene disruption in male mice; which warrant presentation due to their running counter to the retention of health and the longevity of GHR-KO mice. Figs S4 and S5 Corroborating and expanding upon the data in Fig. 1A, we present here further documentation of the crucial absence of an effect of [file acel0013-0981-sd1.docx]

**SUPPLEMENTAL FIGURES AND TABLES**

**Figure S1, related to Figure 1. Organ:Body Weight Ratio Differences, Attributable to Either the *Ghr/bp* Disruption or the RIP::IGF-1 Transgene, in Middle-aged Females.**


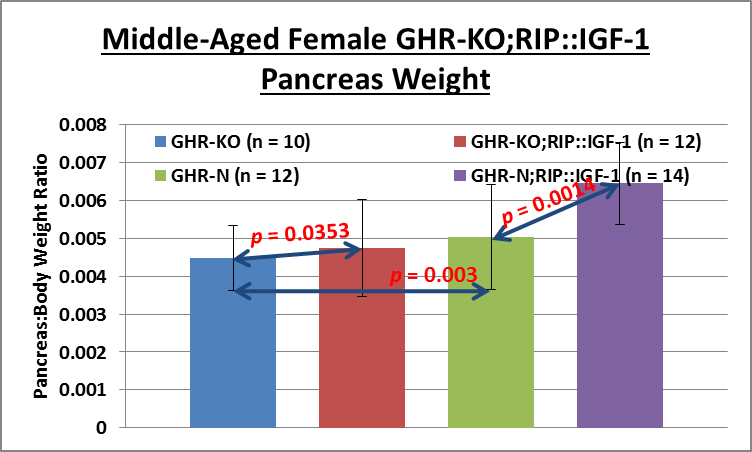


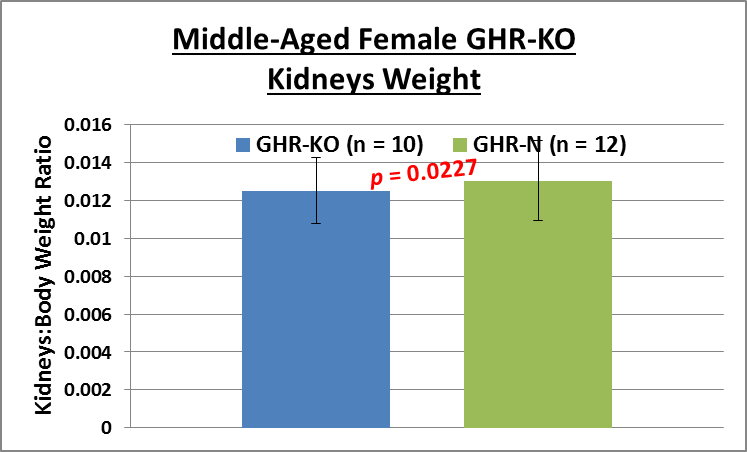

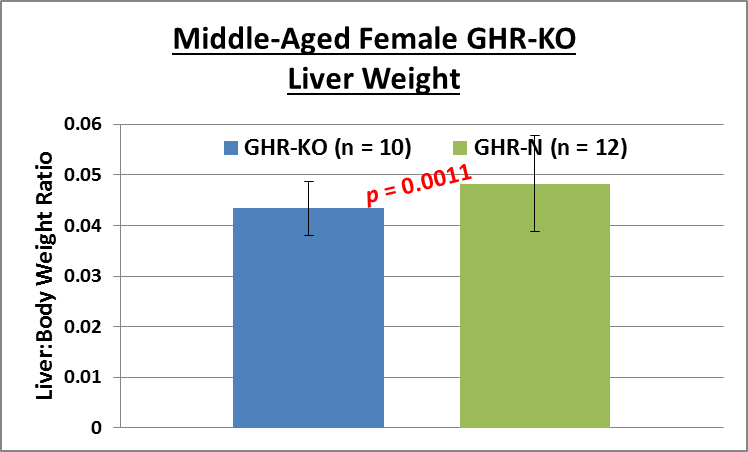


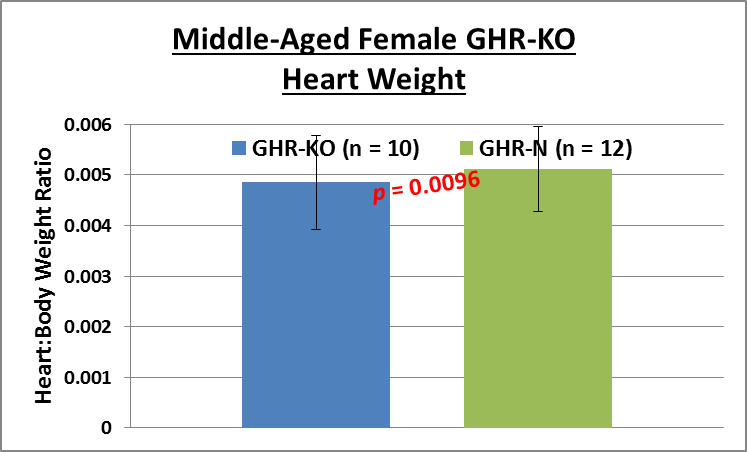


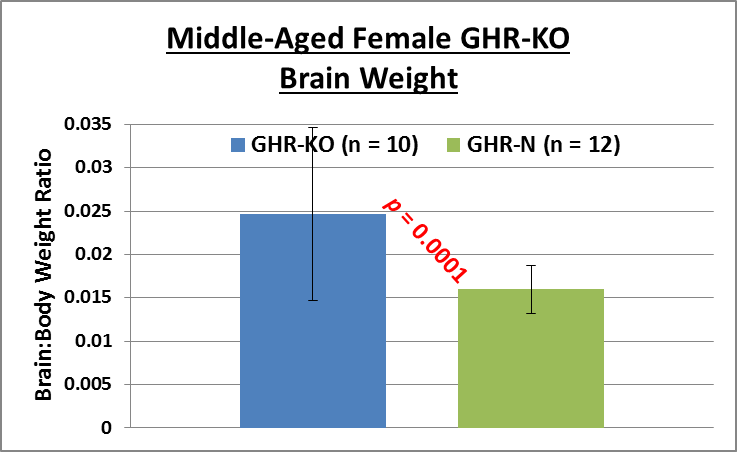

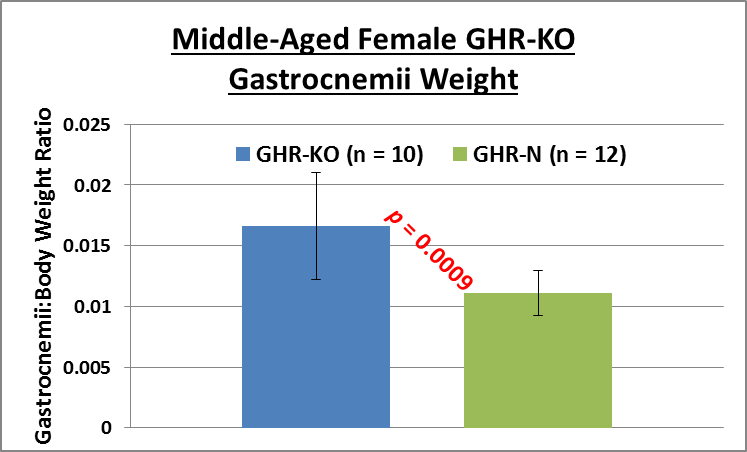


**Figure S2, related to Figure 1. Complete Blood Cell (C.B.C.) Analysis Contrasts Due to *Ghr/bp* Gene Disruption (*n.b.*: M.C.H. and M.C.H.C. graphs are omitted, as they repeat the moral of HGB graph).**


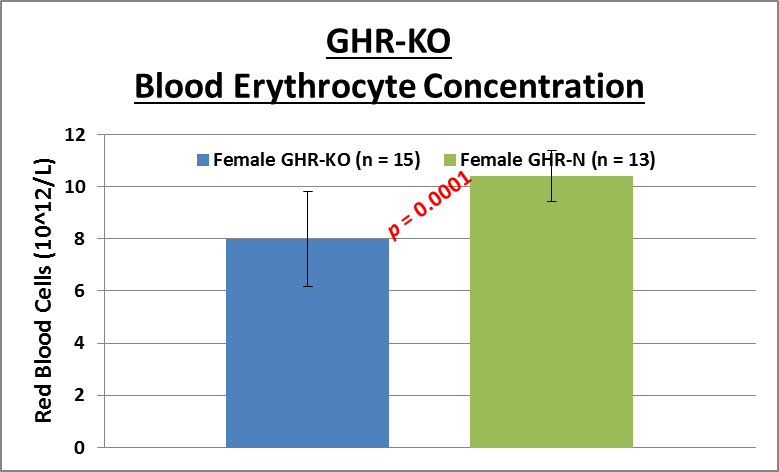

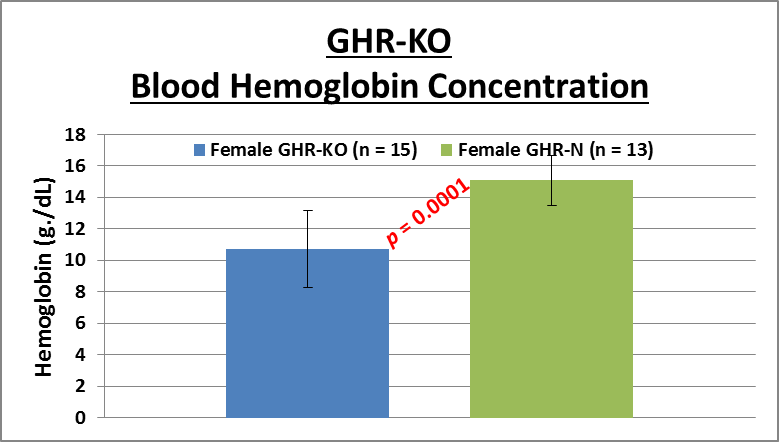


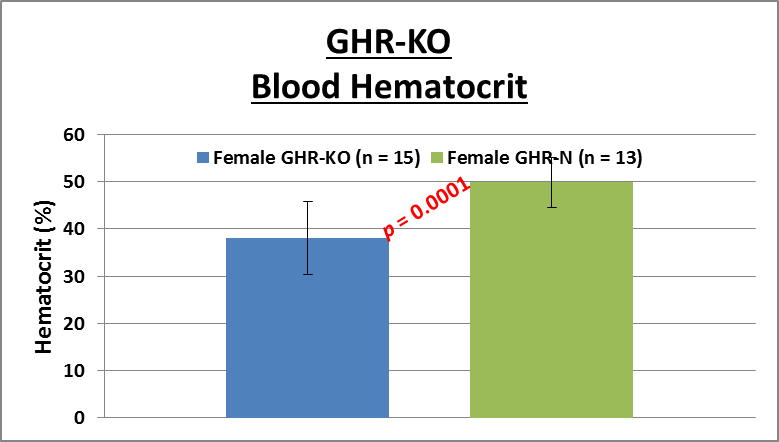


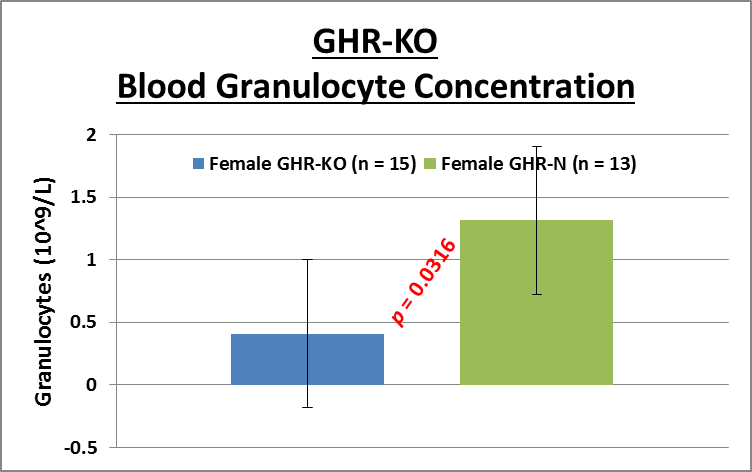

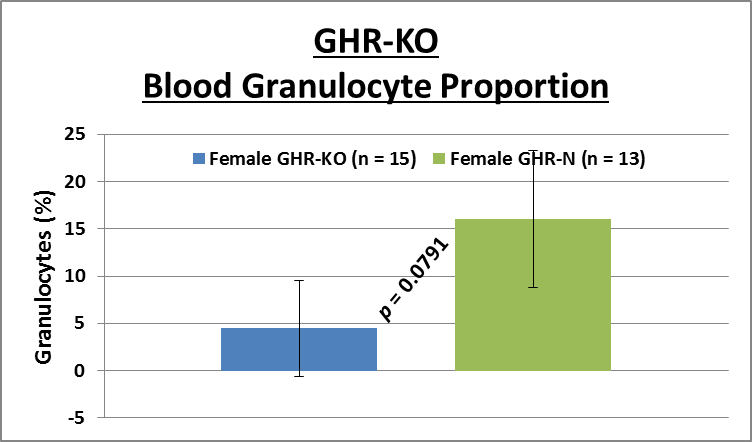


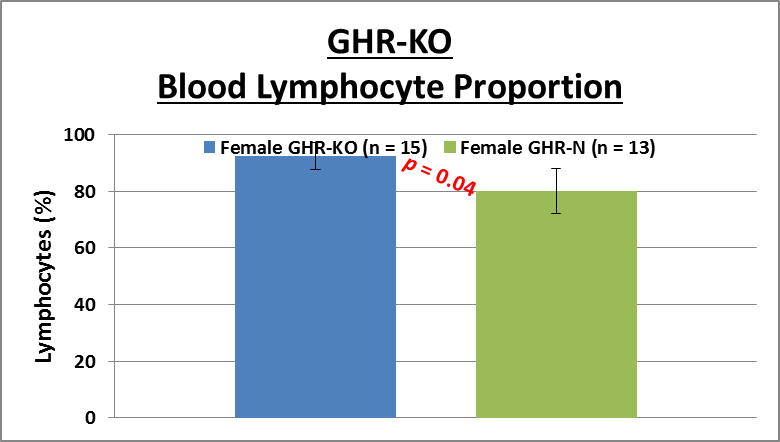


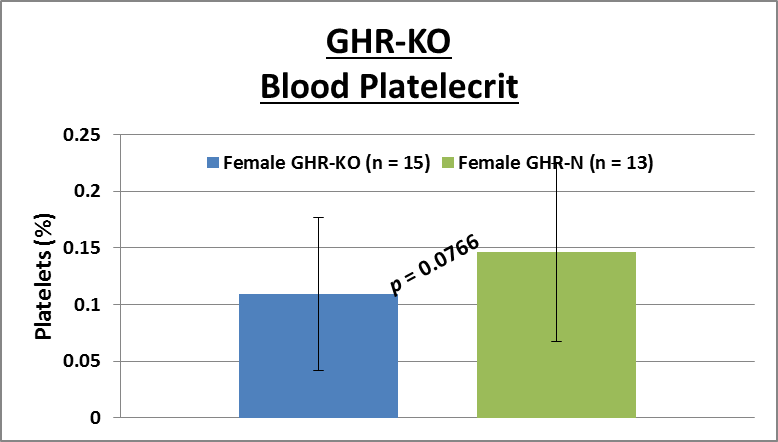


**Figure S3, related to Figure 2E. Differences in Lipid Energetics Constituents (Male GHR-KO *vs.* GHR-N).**


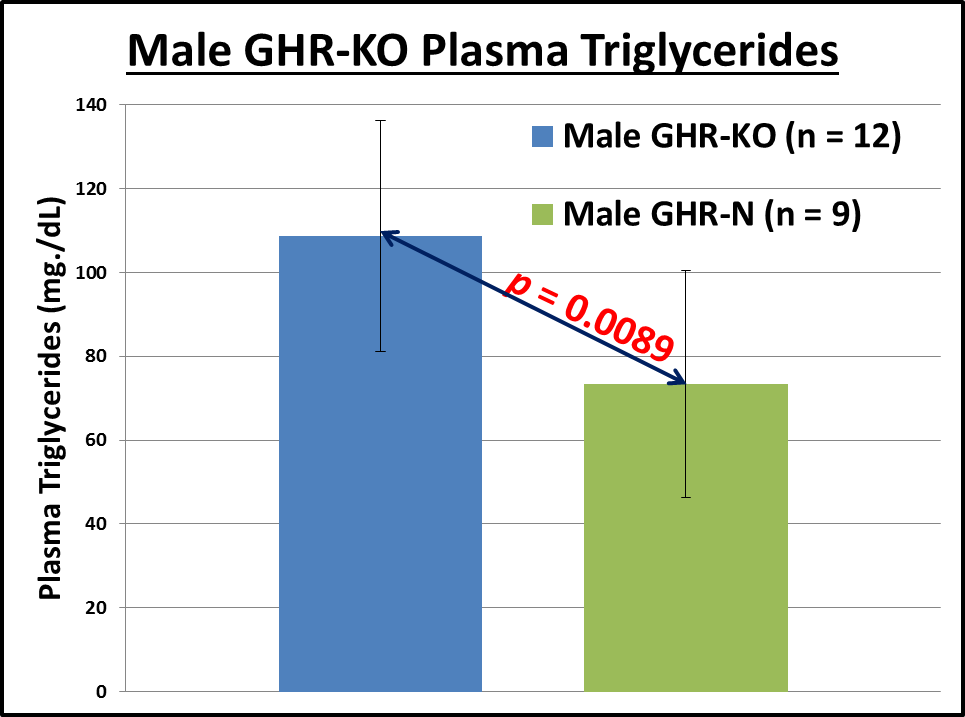

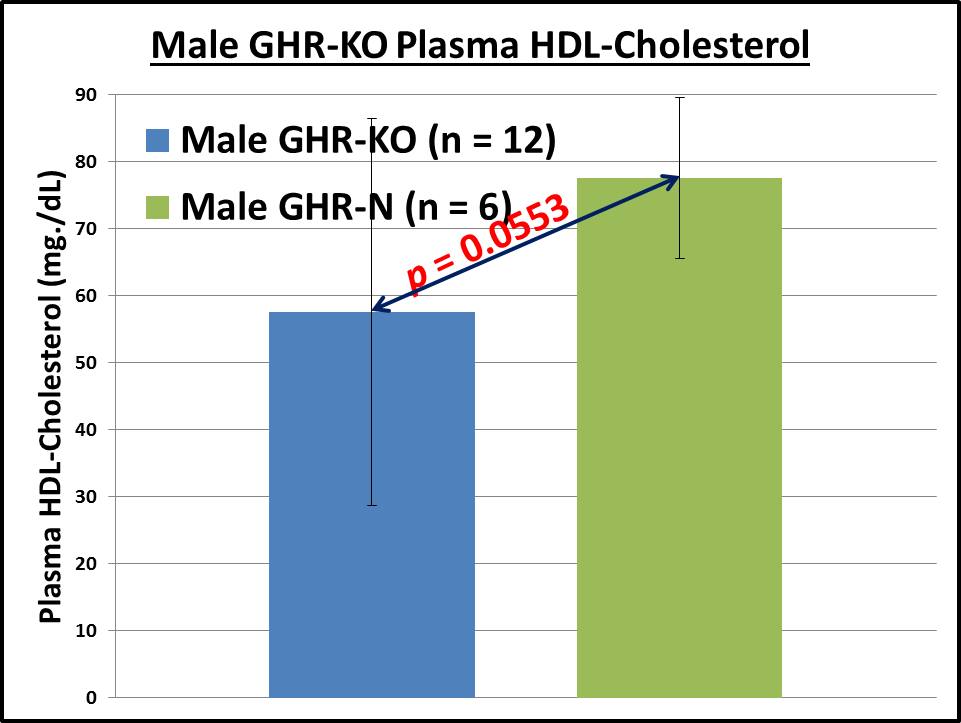


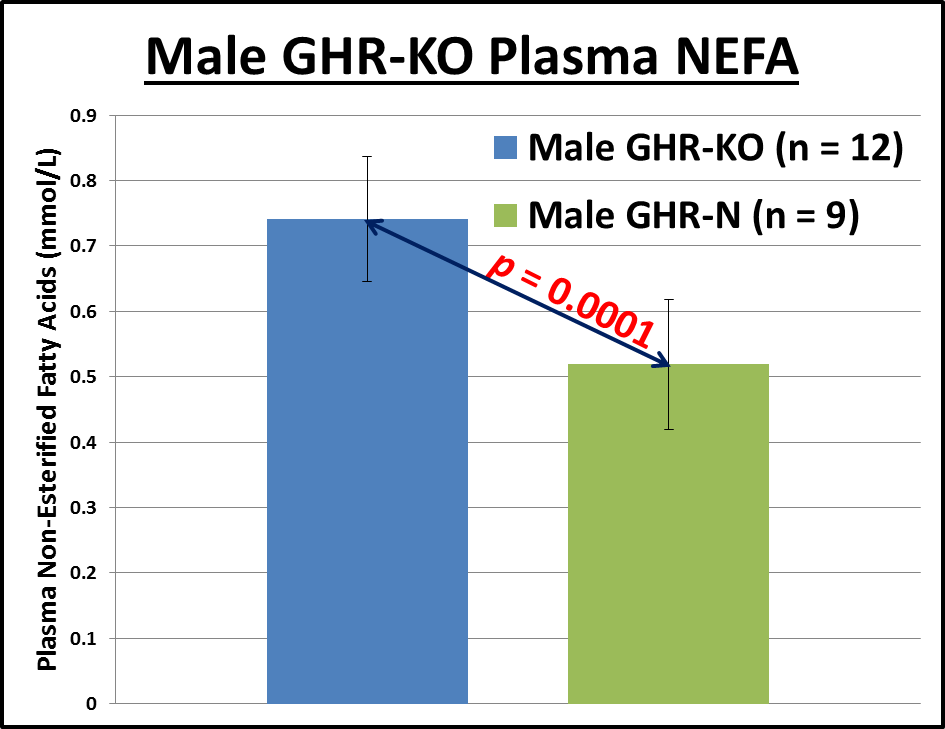


**Figure S4, related to Figure 3. Absent Effect of RIP::IGF-1 Transgene on Body Weight Trajectory.**


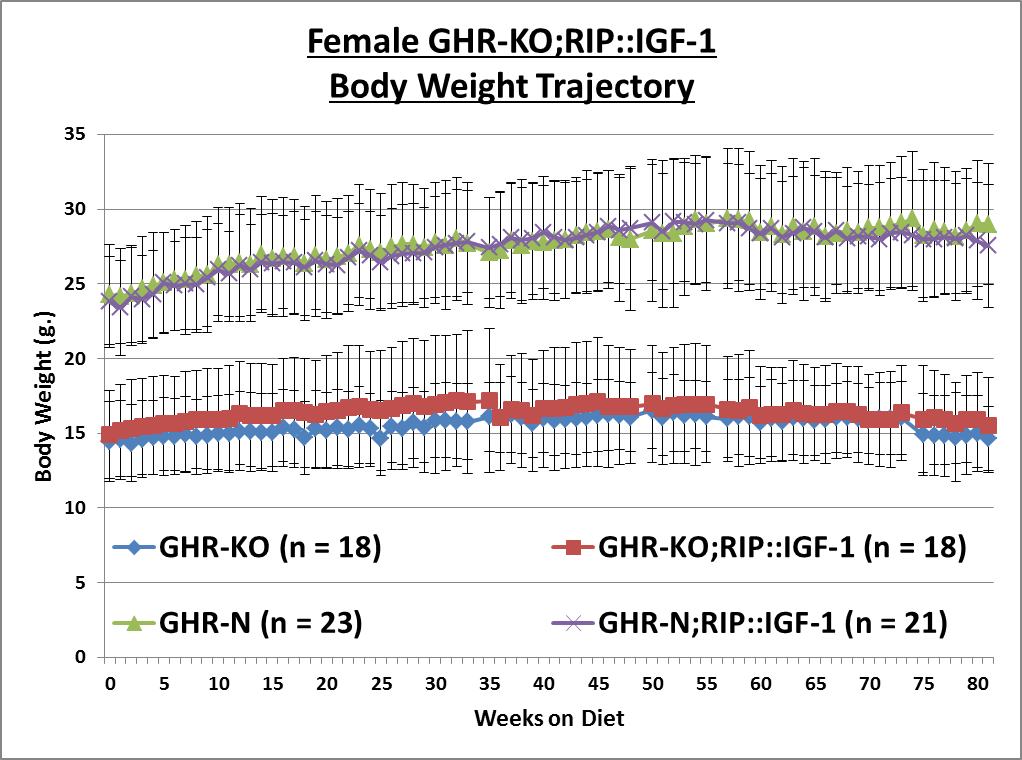


**Figure S5, related to Figure 3. Absent Effect of RIP::IGF-1 Transgene on Body Weight Change Trajectories.**


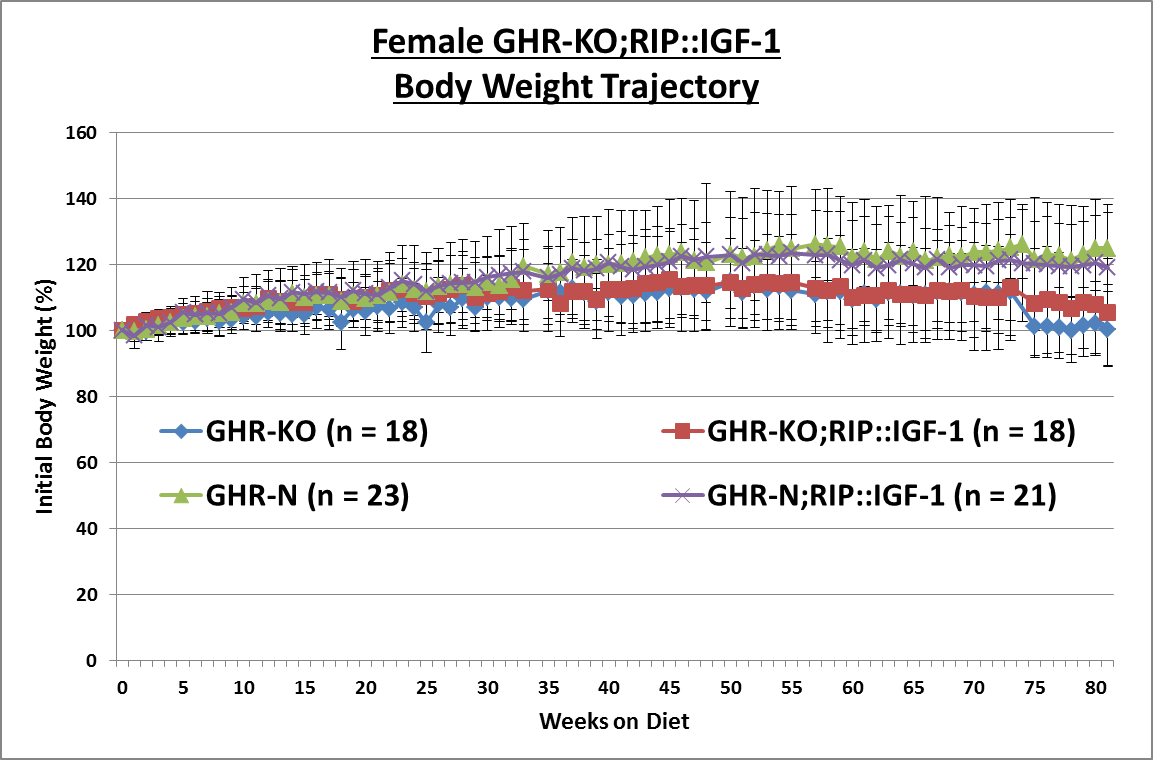


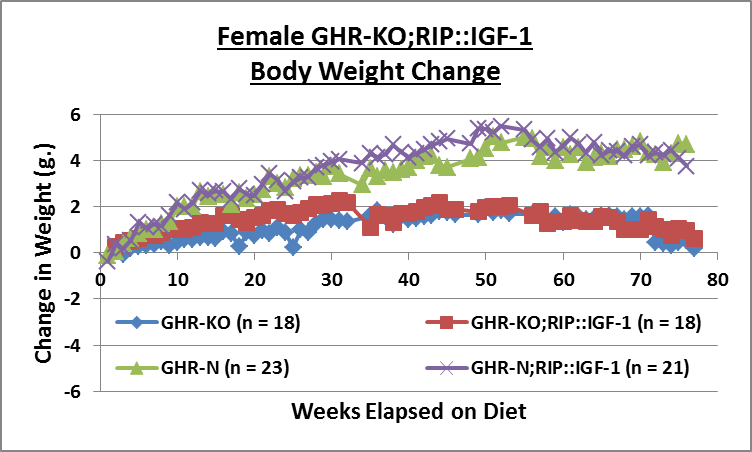


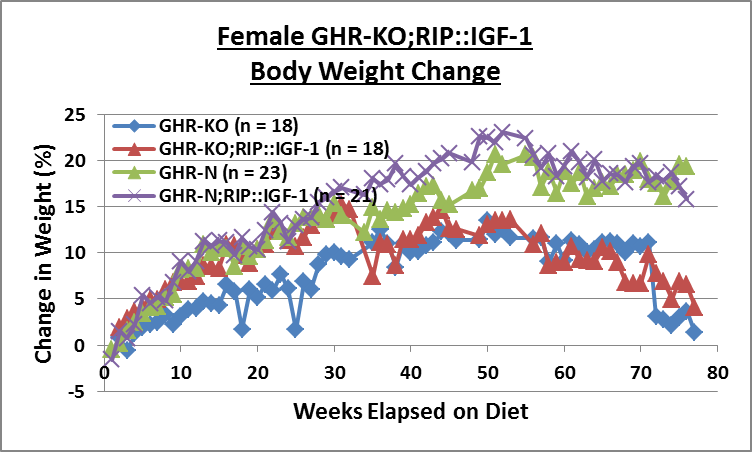


**Figure S6, related to Figure 3A. *ad libitum*-fed Glucose Tolerance Test (Raw Values).**


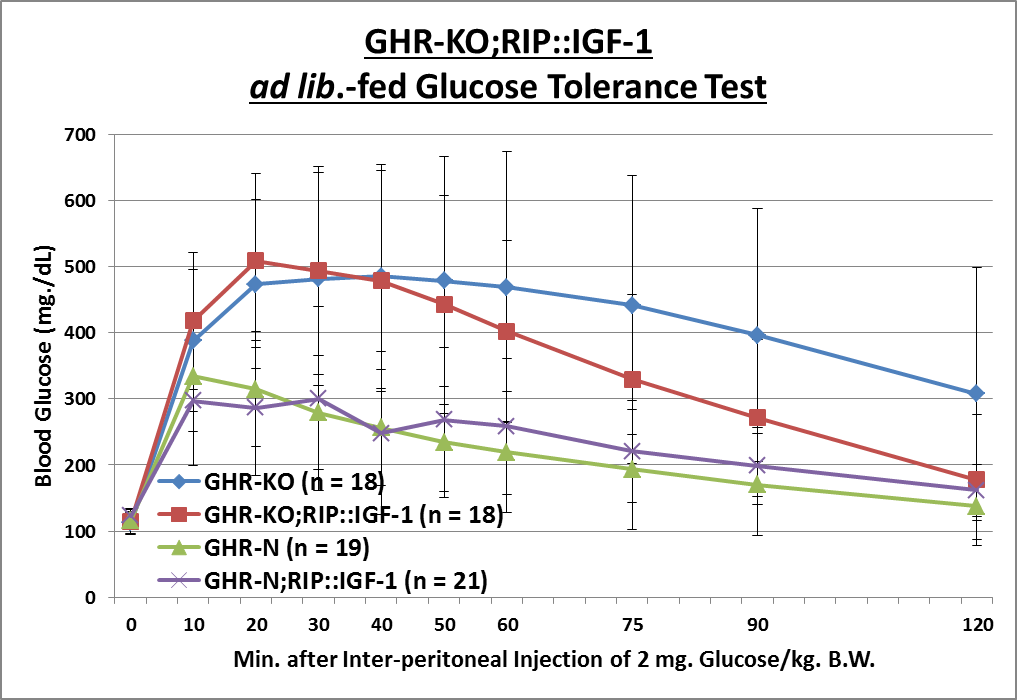


**Figure S7, related to Figure 3A. Lacking Effect of RIP::IGF-1 on Body Weight preceding *A.L.*-fed Glucose Tolerance Test.**


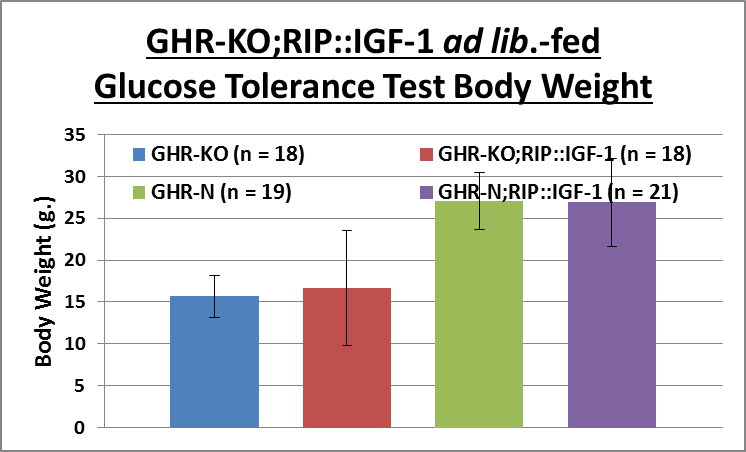


**Figure S8, related to Figure 3B. Fasted Glucose Tolerance Test (Raw Values).**


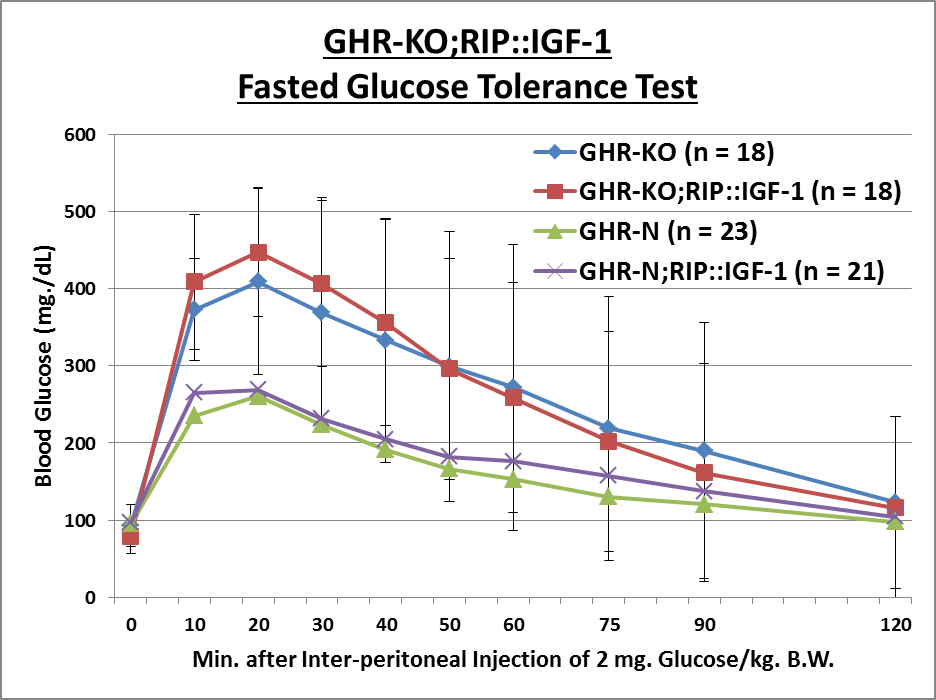


**Figure S9, related to Figure 3B. Lacking Effect of RIP::IGF-1 on Body Weight preceding Fasted Glucose Tolerance Test.**


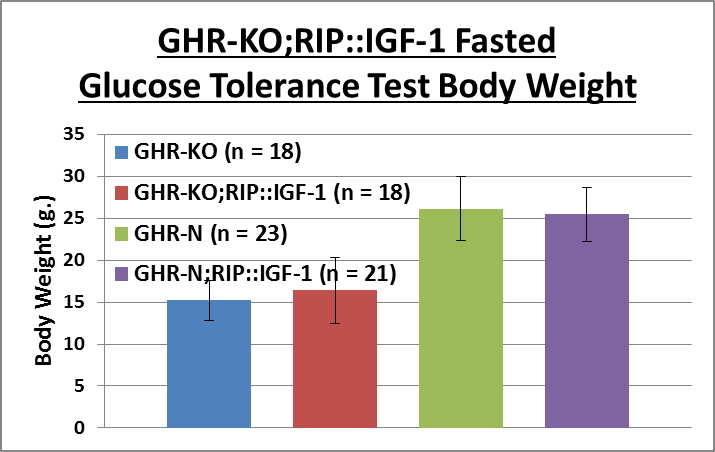


**Figure S10, related to Figure 3C. 0.75 U.S.P.U./kg. B.W. Insulin Tolerance Test (Raw Values).**


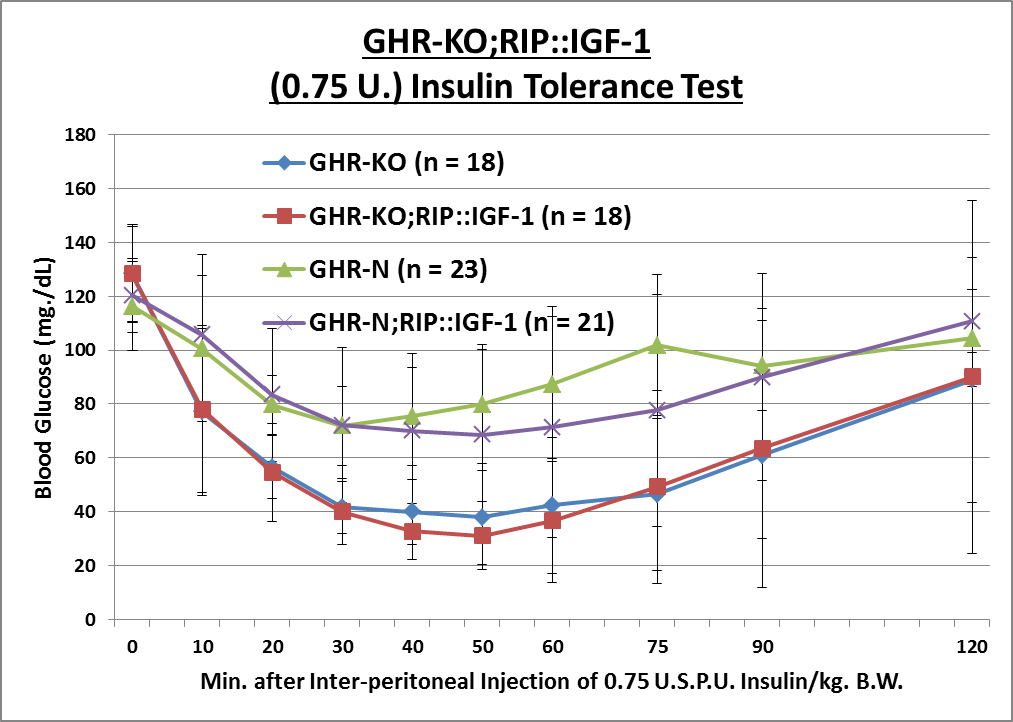


**Figure S11, related to Figure 3C. Lacking Effect of RIP::IGF-1 on Body Weight preceding 0.75 U.S.P.U./kg. B.W. Insulin Tolerance Test.**


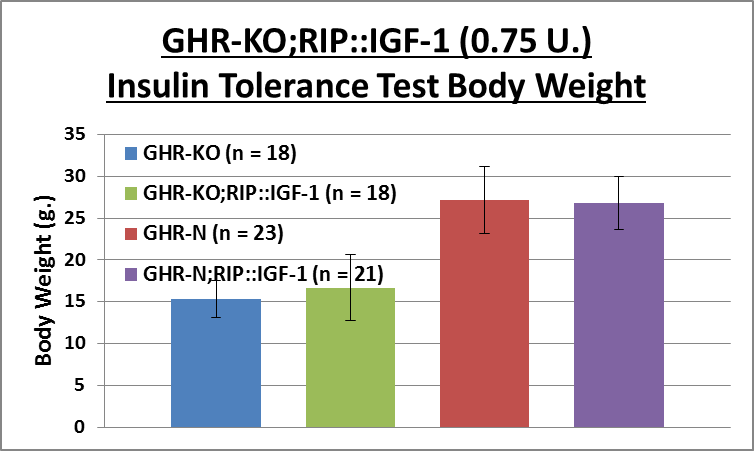


**Figure S12, related to Figure 3D. 0.3 U.S.P.U./kg. B.W. Insulin Tolerance Test (Raw Values).**

**Figure S13, related to Figure 3D. Lacking Effect of RIP::IGF-1 on Body Weight preceding 0.3 U.S.P.U./kg. B.W. Insulin Tolerance Test.**

**Figure S14, related to Figure 3E. Pyruvate Conversion Test (Raw Values).**


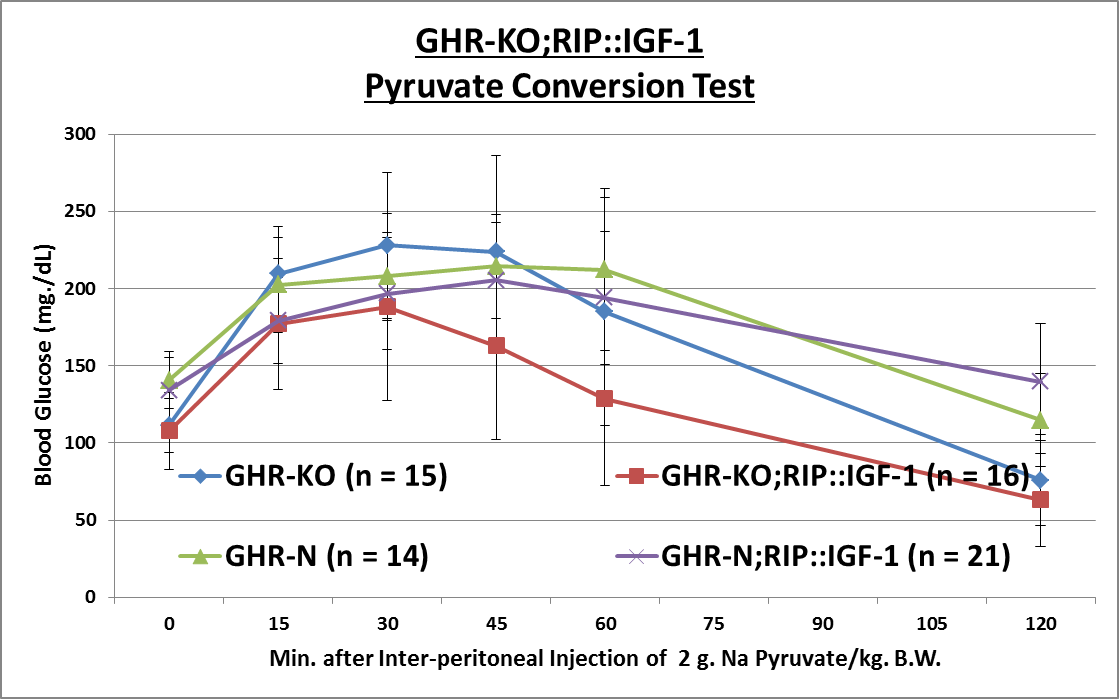


**Figure S15, related to Figure 3E. Lacking Effect of RIP::IGF-1 on Body Weight preceding Pyruvate Conversion Test.**


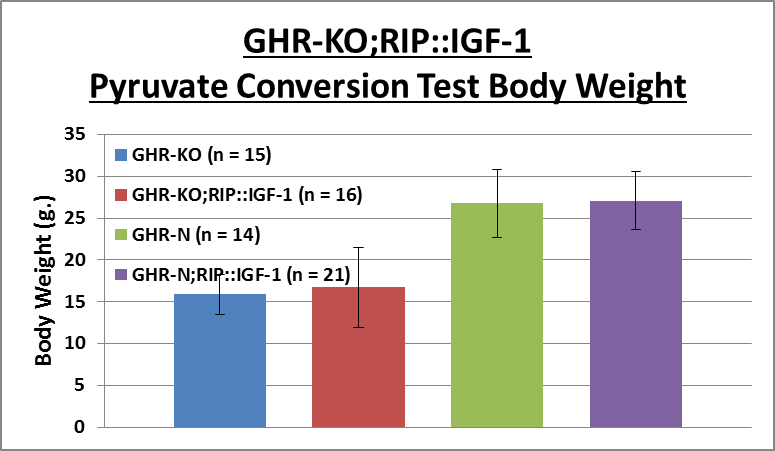


**Figure S16. Absent Effect of RIP::IGF-1 on Metabolic Efficiency.**


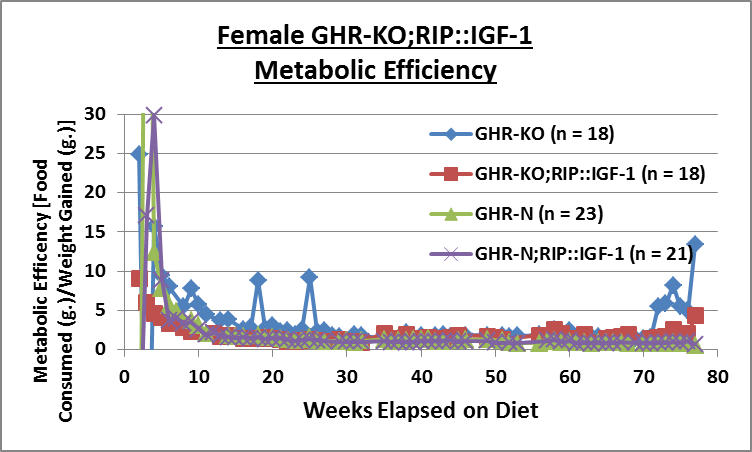


**Figure S17. Absent Effect of RIP::IGF-1 on Feed Efficiency.**


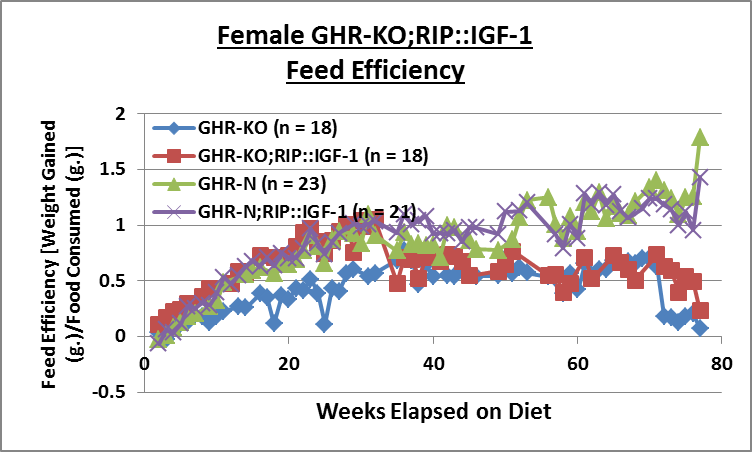


**Figure S18, related to Table 1. Absent Effect of RIP::IGF-1 on Body Weight or Fat-free (Lean) Body Weight During Indirect Calorimetry.**


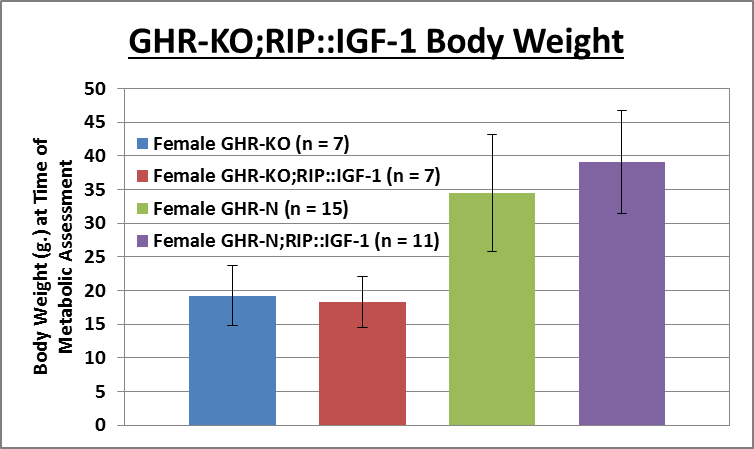

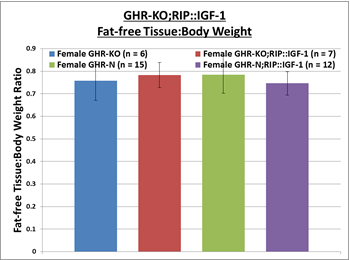


**Figure S19, related to Table 1. RIP::IGF-1-regulated Normalization of Oxygen Consumption of GHR-KO Mouse.**


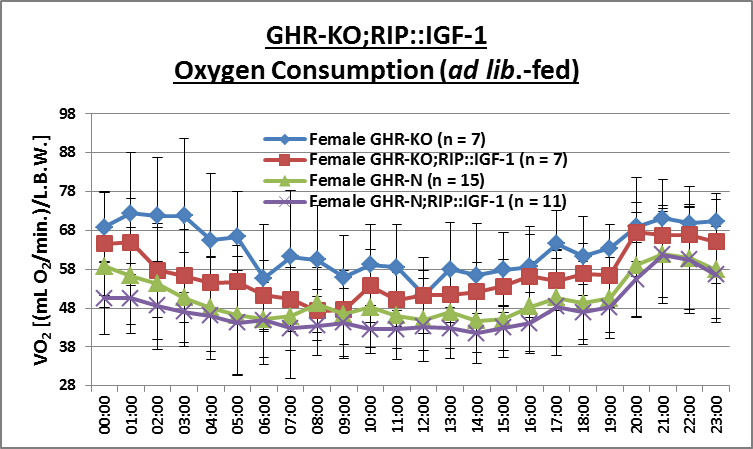


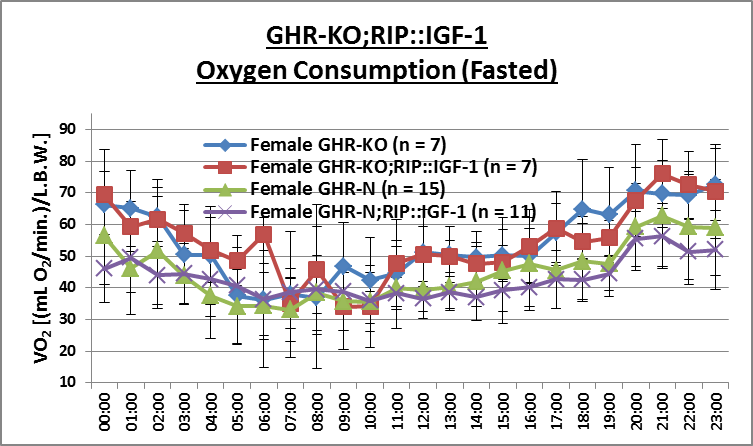


**Figure S20, related to Table 1. RIP::IGF-1-regulated Normalization of Respiratory Quotient (Respiratory Exchange Ratio) of GHR-KO Mouse.**


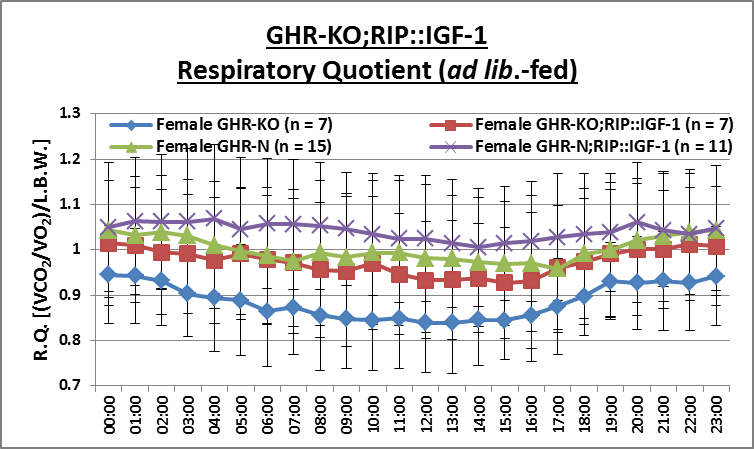


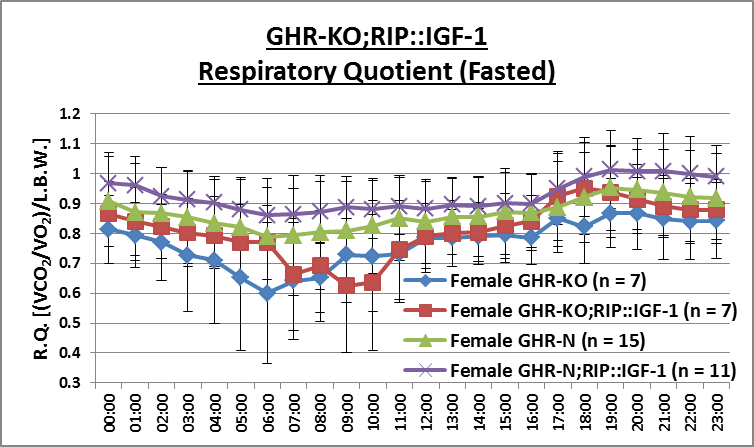


**Figure S21, related to Table 1. RIP::IGF-1-regulated Normalization of Heat Production of GHR-KO Mouse {*n.b.*: Potential inter-genotypic sexual dimorphism, as male GHR-KO’s produce *less* heat that male GHR-N [Westbrook *et al.*, (2009)]}.**


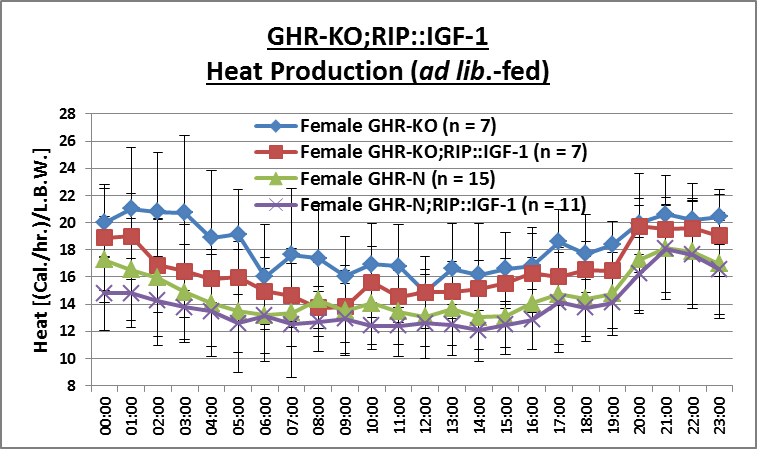


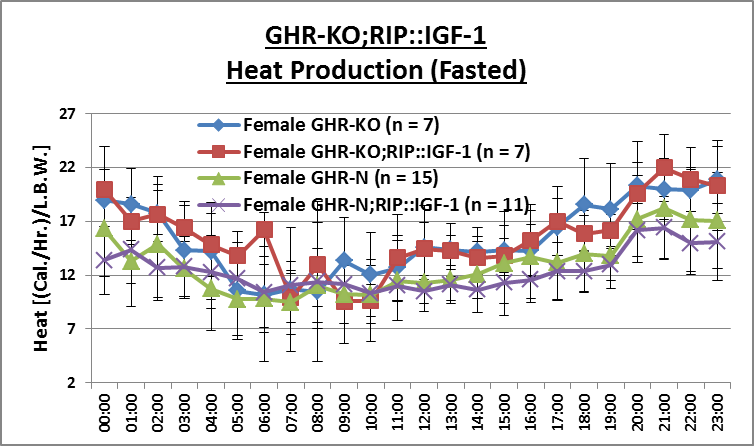


**Figure S22, related to Table 1. RIP::IGF-1-regulated Normalization of Energy Expenditure of GHR-KO Mouse.**


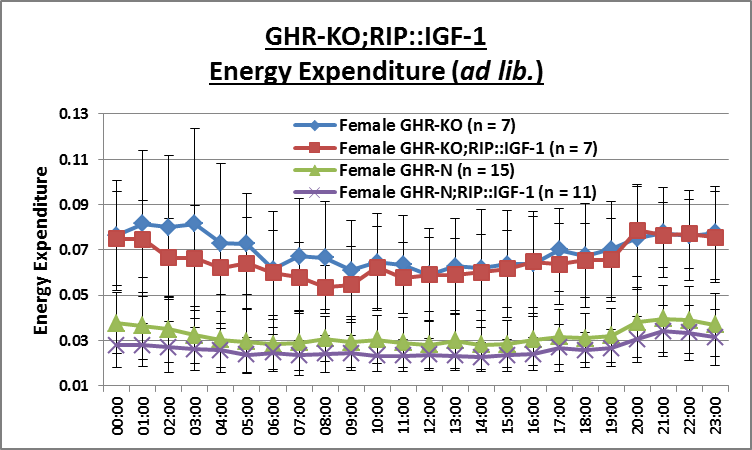


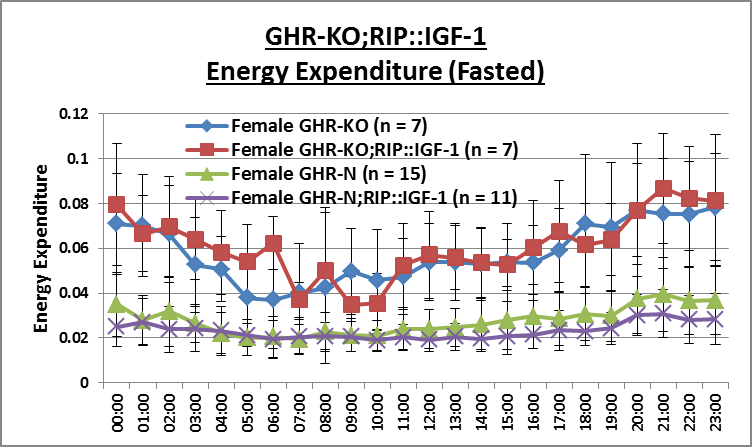


**Figure S23, related to Figure 5B&C. Anxiety/ Exploratory Drive-testing, or Proximal Long-term Memory-testing, Apparatus.**


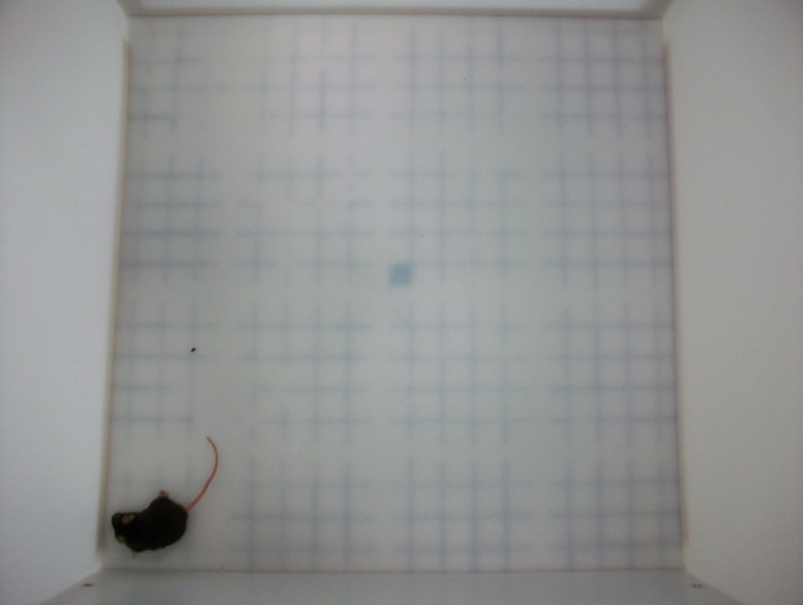


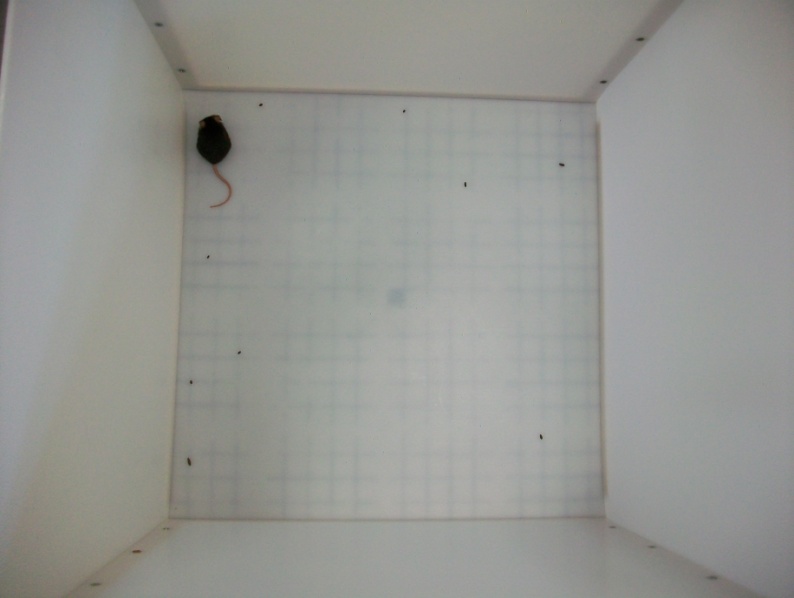


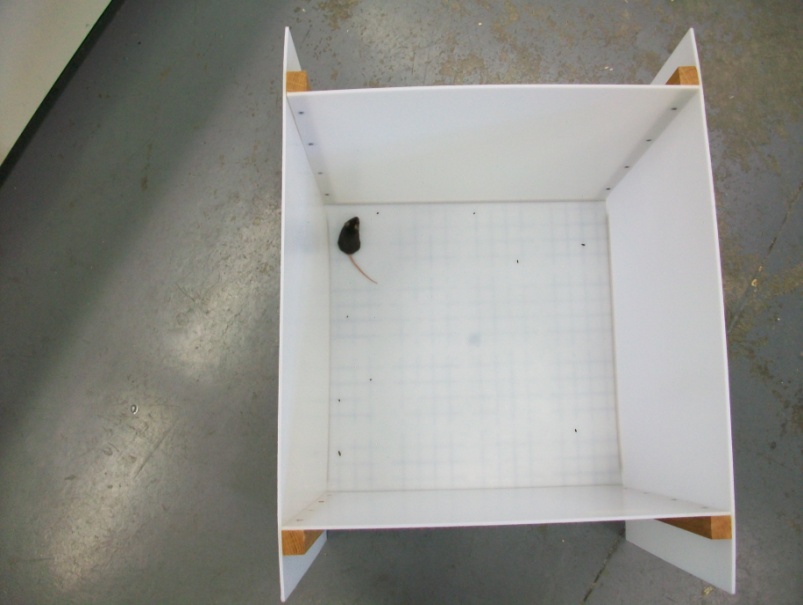


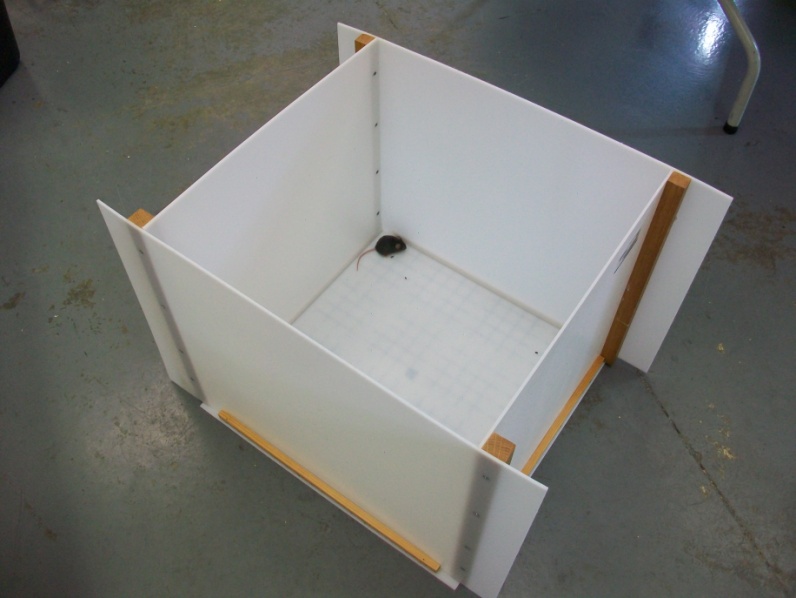


**Figure S24, related to Figure 5D. Passive Avoidance Chamber Paradigm-based Assessment of Penetrance and Expressivity of Distal [(50+1)-day delay] Long-term Memory Performance.**

**
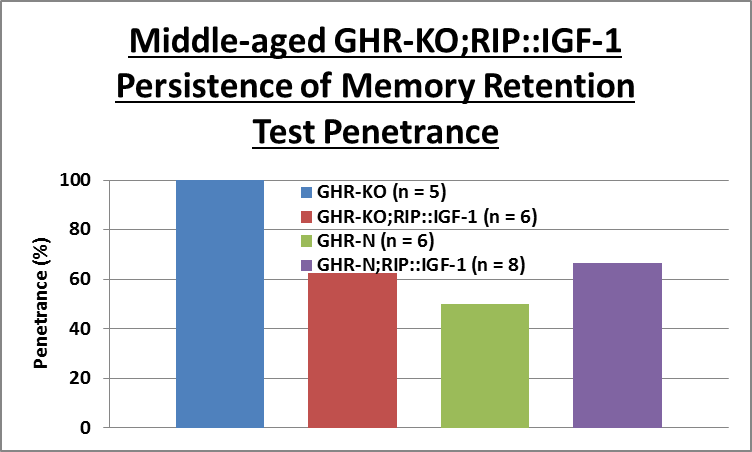
**

**Figure S25. *In silico* Analysis of FoxO3a Binding Sequences about Chaperone-Mediated Autophagy-mediating Genes.**

**Consensus FoxO DNA Binding Element [a.k.a. Insulin Response Sequence (I.R.S.)]**

5'-TTGTTTAC-3' (<http://www.stanford.edu/group/brunet/Calnan%20and%20Brunet,%202008.pdf>) [Calnan&Brunet, (2008)]

**FoxO3a-specific DNA Binding Elements**

5'-[AG]TAAA[TC]A-3' (<http://www.uniprot.org/uniprot/O43524>)

***M. musculus* *Lamp2a* Gene [inc. -10 kbp. (putative promoter)] [mRNA-coding sequence (inc. actual protein coding sequence) in bold&red]**

**(http://www.ncbi.nlm.nih.gov/nuccore/NC_000086.7?from=38405064&to=38456455&report=genbank&strand=true)**

tggtcttatgactccagctgaagcggcgagtccaggcttttgcggtgcctgggtgcctcggtcggtggtctctcgtgctttcgcttccgcagggctgcgaggggattcgttggaattgtcggtccggtggtcgcctggcctgagggatcacgatgtgcctctctccggttaaaggcgcaaagctcatcctgatctttctgttcctaggtgagttttcgggccctctggtgtctttgagccgggcccgggggatcggggagtttcaaaggccacaacctgaaagactggtctttgagaaggctttgagagaccgaacagctgacactgggcggcggcggcccgaggcggcggcggaggggggacgcgggggggccgggagggggggacacacgacctggaggaaggcctggccgtgggatggcccgccaggagggcctctgagagtaacagcggggcaagtaaggggacgagggaggacgacgtcaagggattaggcaagaggagcgcagagttctagacctctgggctagagagagcaacttggaaaaagagtcggggagcagacaccggggaggcttggtttcctggatcaggggagagttttaaagatggtgaaaaaaaagtgtagagcgagaaatgaatttggaacggaaggcgctgtaccctcgtgaaattattggggagggggagggcataggggggtaggtagataggacagtagggcccaagggacagagacaccgttttgggagctttgtaggtaggacttgaggctgggagaagcagtgattgaagagctaaggaatggccttggagttttgagagtgaagggatacacacacacgtacatacatatacatatagtgactggggctggagtaacttccggaaagaaaagttatatccaacctctggagactaaagggatgagttgataactttggtggggggtgagtatctgacttaaaggtggtgaggatctcggtttttttgtttcgttttgtttttttttttttttttgttattgttgttggtttgcttgatttttgttgctttgtttgtttttctcgaataactttcgctacttcctcccagccagacaccggacagaaatacccctagatgctaaagccggggagcttccaagatggtaattgtgtatctgagctttgtatattcaatccattttcttcacaaccgcttggatttttttttttttctgcttcttttttttcctcactacaggtcagcttagtcgtggttttaaagtggtgggaagagttgctgggttaataatgttggttgtttgtgatctgtcccaatagacttgagagtccataacagcctaggcttgaactttgatcccagtgtcctcctgacctgatttactctgctccttgttaccttgtgccttctgctgtttgtgctttaataaagagatagaagaaaaagacccaccagaatgggctggagataggcctattgagggggctttgagaaagcaaaagaattgcatttgtaattcacatgttggtgggatagaatcaactggtacagtgtgttcttacacagtgggcatttggcaatatcttcttagaggcattaatttttttccataatagcttttgaggtttttttttttttgagattgtatttcaattatatttccccctcccttctccacctctaaatcttcccatatacttctcccttcccttattcaaacccatgccttctgtttttttgttattagatgcttataagtatttgtatagatatatgtagtccagaggaatttttgtgtgtcccactgggcagcttcgagtggatagaggccagggatcctgctaacctgcttgcaaggacaggaacagcccctctcaagaaagagttctctctttcaacatcagcagtgccaaatgtgagagcacagcatccagtgggctcctcccgtgactcccaagacgcttactagggagcctttggaatgaaaaccttccccaaccatattaaggtctttcttcttttcctcctagacttttgcttatacacagtagagttttctagatgttgtatacataatatgtgctgatatgattctattgtcctcgccatttcttaatttatcaactttcatttctaatactagtaaatgccagcagatataattcacctaagccgagactctgtgacgtcttccatagaccagaacgttctgactctaatatatttgcaggcccttgatctagctgaccatactgtgtaagaaactcctctaacacttctgaagttgggtcacgaggcttggcacgcacacctaattcttagccttacctgtgaggcacttactggctgacccccaaagagaggcttgtttgcagtggaaaacaccttatcagtgatgagagaacatctgacaaaggcctattaaccagtaattccctgccaattgaatctgcacaaacataatgctgttattgttcagagaaagtgagaaaagtacattacctatgtgctggcttttgtgccattaatttttttttaatgaagtcttttcttttgcatttaaaaaagcagtacacgctcattgcagaaagtaaacagataaggcttagtgcatagaagaacagctcaaacctagtggcttgtagttaactagatccatccttgtagatgtttcctgagctgattccatccacaacttcagcaagggagttacaggagcaataatgtaggatgcagggttgggtttttttttcatattttttgatttatctatattttatttatgtatttttctctgcctgtatgtctgtgtattacatgtgtccctggtaccagtggcagtttgaggggggcaatcatatccccctggaactggagtttgcacagacactcgtgagttgccatgtgggtgctgggaatcaactgggttctctggaagagtgatggatgagccccctccagcccccacgatctagtttttaggtctgcacagctcttctttcaggcatatcctggtttgctgtcaggctgaggaagattctgcggatcttaagaattgtttttggattttacgttttactttttttttggtttttcgagacagggtttctctgtatagccctggctgtcctggaacgcactttgtagactaggttagcctcagaaatcagaaatccgcctgcctctgcctcctgagtgctgggattaaaggcgtgtgccatcacgcccggcacatgttttacattttcatgtcaaaatggacacccaagaataggattttgaaaactgtaggcataactgtgtcttggtacttaagtgggtaccagtcctgaatgtgtactctgcttggtgtactggttcagcttgcaaacttcataattctattccatctctagcagtggcctcttaaaaataactatttaagtggagcagtggttttgcacgcgtttaatcccagcacttgggaggtagaggcaggcagatctgtgagtttgaggccagcctggtctacatattgagttcctggacagccagggctctttagcagagaaacctcatcttaaaaaaaaaaaaaaaaaaacaaaaatctactttaaggttagggctatctctcagttgtagagagcacttacacagagtgggtgaagccctaggtttaatctccagccatttctgtaaaaaaggaagaccaaaaaccttgctttaaagtaagtataggagatactcttaaactttaaattgttttccacagacatttattttttaattttttaaaattatttttctttttttttttttttaaggcagggtttctctgtgtagtcctggctgtcctagaactcactgtatagatcagtctctctgcctcctgagtgcagggattaaaggggcggaccactacaaaaggctcacaaaaaacttctgttggctttcatttgaatgaattacaaacaatgaaagacaaccatttttggggaaaaggcctatgagttagagacatgaaaagggcaacagatgaaagtccttgcctaacatcattgcttaattttgtttccatcgtttcctctgtgctcatcgatttttataactagcctaacctaacttctgttttttttatttttcctcagagtttttcagtgaaatagccctggctgttctgaaactcactctgcagatcaagctagcctccagctcacagagatccacatgtctctgcctcgttagtgctgggactaaacatttaccccccaccatgccccacttaacttctgggttttctttgtgtaactctggctgtcctggaactcattctgtagaccaggctggccttgaactcagaaatctgtatgcctctgcctcccaagtgctgggattaaagcttgcgccaccaccgcccagcttgagtgagactttttaaaaaaaagctttgttaactatcctgagtgtaggtcaggttggggcctctccctcccagaaattattaactgcctactgtaggctaaccacagccagatgaaagggaggcagcaatggacagtggagggtctgctgtcctagagcctacattttagagggtgaagatagccctataggtaagtaacatttcggatagtgggacaaacaaaaggctttctcgtgttgagtgacgttcgggcagagaccttggtgctgggaggaggcccaccatacctgtaagcctttgggatggtgtgggtgaggaggctctgggaggggatggggggtgggaaatcatgatcaaggctaataggaactttcagtcaaaatacaacaaataaattactgaagctgaaaaaggagtatgttttccagctcttagatgtagtagcctattttctgtttttatggtcttagtctctgagtggaggaggtgaccggtgttggcggtagctagcagtagatctactgtggagtggctgaaggtcagtaggtaagatggtgaattagaccaaaggtgatctctttttgtcttccgtggctcttacacggtgttttacaaaaagctcgtttgtgtttttgaatgagcagtaagagttcaggtaattataaggctggtctcatgtgataattctagtctctcctccttgggctgctgtttagggggttaggggaaacacggtctcactgtgtagccctggaacactctacggcaaccaggccaacctcaaatgtaagagctcacctacgtctagctcttcagtgcagggatttcaggcatgcaccttcacatttggcacttgaactttgcctgtacaggcatgaggcccaacgtggagagctaagaaggacttggtgaggagaattaattttctcaaacaaccagcatctctctttttggagaacatcaaaaaatgtttaacactgttttcctgttggtttttttttcccctctgtagccttaccacattttgtttgcacagtgacttttgattttatggttcaattaaaattcatctatctgtggtagagatacttgaaagggcaggtttgacaatttccatgagtcattgggttatcacatctgatagggttctaaatatttaaatattcctgtgtgtctagccttattttcctggaattgacggtgacttttgtctgcaacccagcattgtctcagccctggagagagcttctggatcctgcttcgtgaaggctgcgcttcctaatcgcagtttctaaagtgctcggagattcttccattccagaagtggattttatgagtgcttgttggtatcccagtgtgcattacaaaagcactctggaggcctgccaactgtggctcagcaataggcactttccattacttcaggaaagttgtctccttttcttacctactctctgaccttaaacagaatcaaggaagtcattttatggtttagaacattatgttttgttagttttaactgttgcttttcacgcaccaacattcatagcttaaaattgtattttctgttgtaagcagacctaacaaaaaggaaactgtaaacaagggagtcacaggtaaacatgagaacagctggccagatgtaaactcatgggaaacttgtcacttagcagagaaggaaatgggagccttttgtcctgcagtgggtttttcagtggtcttgttctgtcgtccaagctcacctgcaactcctactgtgtagcctggattagcctggaattcatagcagtcctcctgcctcaatgtcctgagtgctgagattataggctatgccctgttttgtagagtgtgtgtattcatgtatgagtacatatgtgcatgttgtacatgtggaatgtagaggactacttgcaagagtcagttcttctaccctgtgggttctaggaattgaacttagttcttagttcctaagttgtcaggttggtataagcagctttacttctgaggcatctctggccccatgccttttaagaaaaacatttatatgtatgcattttctgcttgtgtgtatgtctgtgttctacattcatgacctagagaccctcagagacaagaagagagagttggttgccctggagctggagttaaggatggttgtgagcaccataagggtgctgggaactggacccaggtcctctgcaagagcaacaaatgcttttaaccactgagctatctctccaggtctgtatgcctccctgcctgccacaaacaaacaaacaaacacacacatacaaaaataaaccaaccaaaaaacccactttttggccaacctcaaggtaaatggataaagtttagggtaggacttaatgggctgaaagtaaagatacattttagaatgagacagattccctttctttacatcctgtgtttattgaaagggaggatctctaacttgcttggaaaacttctcttagcttagagtgtttataccccatcgagaaacccattagacaaatactgcagttcttggcaagagtaactggcccagagtctcccagctagtaagtttgccttcctgagtggattgtagcttttcagctctgcacatgggtggtgagttctcgtcgtcattccattctattgtttctgagatcactgttacgtgggctttggtttttttgtttttgtttttaattttatttctatgagtgctttgcctgcttgcatgtctgccatgttgtgcctggtgcttgaggatgccacgggagggtgttgggtcttgtgggactagaattaaagacgattgtgactagccatgtaggttatgggaatccagcctggctcctctggagtgaccgagccatttctccctcccctgttatggattttgtttctatcttctatctattttcccccaaaccagggttattctgtgtaacagccttggttgacctgtaacttgctctgcagactggcctcgaactccgaaatctgcctgtgtctgctgagtaaaggcatgtgctggcatggtgctttgttttagtctgtgtaccttccaggcaccttttgggccaaacagacccatgaaaacttagccacttttttcccaatggtttgtgtttggatagctgagttctgaaaacctatacatgaccatttggagcagccttgacagcagcattgcactcgagaatgttgtttttgtgaggcatgatcttttttcttttctttcaagaataaagtctcagctccatgatactatgatacattcagctatggctttgaaaggccgtacagccttcaacatacatacagcctttaaaatagtttggcaattctaaaaatcaagcatagcagttaagtgtggaaatcacttgtcagagacactctactcaatcaagtattacatttcaaaattaatctttcactttatggggaaagccattattcatagcttgaaatatgagtcacagtttatagaaaattgacatgagccttgtggcaatatccagaacacggataagttcatgatttggaagttcaaaaggaaactggcctcctgtaatacaactgagccgtatttcaggtccgcattgagggtattttcactgtgatataatcgatcatatttattgaaaacctaatatgtattcagcattactaggaacactaatggaggaacagtcacggacctgttcttcagtgtgacagacaagatagatgacattcatataaaagacactctttcgggtctgtggcttagatgagattttaggattaaaggagcatattggaattcagatgagtgatggaagcatttgcaaagggaatctatccacactcatcacccactcacagactttggtatcttattaatcataaattgcttactttacatcagttgccacagcatactagtttgctgtgaagtggcagttcggaagtgctatatttaggagattttgccagtaggagagacaggagtcaataacaaaataagttggatttgcattgtcctcagagctgctgatgttgaatacttttaaatgtatttactgaccagttgtagttcttcatttgaatagtatctgtcatttgtccctataataaaaaatactgaaataacattaaaaatatcagtaattcaagatagtgtaaagtccacggagtgtgcaaatatgttttatggactgttctgtgagtgcttttatattccagggaactttgtggagaagtgagatagcatgtaataagtatgggtcccatttattattattttttgaaacatgttggttgtagggggctgtagggttggttgtagggggcttatctagcttagagtggccttgaactcatgatctttctgcctcagcttcctaagtgctaggattacaggcttgcataaccgtgcccagcttcagtctcagttacaagcaggggaacaaactctgatggattttaatcaaaaagggagtttattgaaatcatagtggggtagggtttggtagcacaaacctgtaattccagggaagaaaatggagttcttagccagcccggaaaacagtgagttcaaggcattctgggatatatagcaagactgactgggcagggtgattccaggggatttggaggcagaagcaagtgatttctgagttcaaggccatagtgagacactgtctcaaaaagaaggggggggggaggaaaaaagactgtcttaaaacagacaaaccagaaaaccaaacaactccttcccccgagtagcaataacaataaaaccggaaaactgtagtgggacatattatatagtctttgaaaccataattccattgttagataaatactcaacatacaggcttaccagtccatgaaagagtgtttacatctgcctctgtgatgggatagtttgggatctttctagctgtccatcaacaataccacagatctatagcaaaggttcaccctgacatcagaagcagtagactagaatgattgaattgcagtcataccacagtgaatctcacaggttgagtggaaggaaacagacctaaaagagagcatagtatgttattctgcctataccacgccaagaaaataagaggcagtagccaggatggcattttactttggaagaggaatagtagctgaatggagaagctataagaatttgggaatatagtcagccttttctgtcctctacaggttccgtatgcacagattaaaccaggtgtggatcaaaggataagagagaaaaagtatactgagtataatgaactttcttttgtacattacatttattacatttatgctgtattatataagggatccagagaggttatagaagcatgtggatgaattatgtgcaaatacggtgtatatcattttatgtaagggactagaacatctgtgaacttcagtatcttcccagtgtcctgaaaactgttcctgtaggttccaacactgtactttgcaatgttctgcttcttggaggagggtttacacttgcccatgtgataaaaaaaaagctctgagctgtgtatgttacatctcagtaacattttgctttaaagtgagcaagggctgggcatgtagttg**tgtggtagcacacttgctccccatttcaatctcctgtcccaaatcacaaaataatgtataaaaaaaggttcgtagtatatcagtttacttagacagaggaagaactctggtcaggtataccaaaacctaatatcccaggtcatgctactggcttgcattgctatgatcaagtgaaaatagttaagcttctaccattggctagcatac**cttttttctgggtacttgaacctgctcttttccttgcccataggatgctttttctgtagctggttccctcctcagctcttttacttttaagtctgagtggagttgttagggacagtttagctgtaagggaggtttggaaagcaagtagtcaacatttccagtttctatagcagccaatgatgtgtggaccagcagttagaatcatgttgaaggatacagttattttacctttacagccccaaagtagtagtccttaaggtgaagatttaagccatcagaaggaagtggatctcaagtgctagaaacagttgaagtgaagctgagtgcagtgtagcctttgggcccagtggaaaccagttcaaccggattaaagggtgtgttctgggaaaatggtctgaatgatggtggagcaatagggtgggacaagattaagggttactttgagagtcaggaatttaaatttagtttggagtttgggaaccagtaaaattaagcctgaatgttggagaaagaagttgtatctggcagtgctttttagggacagctcagaggggtctgtagtaatataaggatagagtggtagattgggaatagaagtaggaagaaaatgtgaggcctttgcctttgtatttcatggtgtgggatcaagggagtccttttggaggaggaagtaaaaaatgaatctggattttggaattgtttagtttgagctgataaacctatgagtaaaagcagtcctgatgaaggtggacttggagaaggatggctaggtgtgtccgagtcctggggcatccctctgttctcagtcttgttccaggattggtcaagattggtatcctgagtttggtcgaaagggatacaacccagactctcttcttaaagcctttccttttcgccatggcaaattgatcctgttactatcttccttctttcctttaattctttctttcctttaattctttctttctttctctatttatttgtctatttatttatttttttgagacagggtttctctgtgtagccctggctatcctggaactcactctgtagaccaggctggcctcaaactcagaaatccacctgcctctgcctccccagtgctgggattaaaggcctgtgccaccatgcctgcctggcctttcctttaattcttctcttctcttcccttcccttctcttctcttctcttctcttctcttctcttctcttctcttctcttctcttctcttctcttctcttctcttctcttcttttcttttctttagactgggttttgcctgtagccttagctgtcctggaacttgctctgtagaccaggctggcctcgagctcatagagctccacctgctttgagaacagcttaggttaactttttacccgcctcagcatctttttaaatatttattttaattttatgtgcatgggtacttttgcctccatatatgtctgtgatccattgtgttcctggtgccagccttggccagaagaggacatcagatctcctagaattggcggtaatgttggttgtgagcccccatgtggtgttgggaatcaaagtcgggtcctctggaacagcatctagttctcttaaccatggagtcatctctccagcccagcttgcatatcccccctctttttctttcattccagtatttgagatttattttttaagtatattagtattttgcatgaatgtatgtttgtacttcatgtgggtgcctagtacccgtagaagccaaaagaggacatgagattccccccagaactagaattttagacagttgtgagctgccatgtggttactgggaatcaaacctccatcctctgcaagaacaagtgctcttacccagtcagccatctctgcagccccgtccctcagtttcttggatgctggggttgtaagtgtgcaccattatgcctggcttctcaattactttcataatagttttgagtggtttgggtagaagaagaggatcctttatttttaaattgattttaagtaacacataaaacaaaattgtacacagtaaaatgagatgtcatgatttttggatgcatgtgaataattctaattttaaagaatcaaactaaacattgctccctggtgttgctcatttctttatgggaaagttttggaaaaatcctttccttttgaagtgtgcagtatactgctgctgtgccgtgcagtgtgttggtgttgaagcgtatcagggttgcttactcctaagtgtcctaactgttatttagtttctcttgattggcatgtcttcatcccttctcatgtactctgcccagcttcttgtagccacgattttacactcaacttctaaagactacagcaaaagagagatgcacactcatacctgtaatggatatagatgcaaaactcttagaaccatagcaaattgaatgccacaacatattaaaaagatcatttatcatggctgaggaggcttgacaccaggggtataatctggggaactttatgccaagctttgagatcaagcacaggcaaaacaaaactccagctggttaggaattggaagttggagttctcagcagcactctacaggtagacttggatctgcttggcagctccatttcatgtctaagaagaaaagaatggtggcaaaaacctatgttccttatccaaggtctttctgaaatgaatctttgccaatacttagtcattatcagccgccacaagctttagcatagtgtttgggcttgctaaggcagctagggctctggagcatgaaaggtagccttttgtgtatggcttgaaagcggttgctgaggcatgctggcttggtattcctcttaggagttttctctttagtttgatagcatgtctctacctgtaaatgacactggcttgcttggatagcattaaagggtgtgcagtgatcttaacaggaagcagcgtccccttgggtaatatattagacattcaagacaattctcaaaaggttgccagttatttatgttgcaggtgaatttgctggcatattttccccttctccattaaaatattgacactttaggggcttgagtttaataggcgatgttgcaacaaccttttttttttttttttttttttttggtttttcgagacagggtttctctgtgtagccctggctgtcctggaattcactttgtagaccaggctggcctcgaactcagaaatccgagtgcctctgcctcccgagtgctgggattaaaggcatgtgccagcaccgcccagcgcaacaacctttaagtaatagaagattagagttagacaataagtcccaaagcatttctgcctgaaagtttctctctccatttacagaaggagcaggttgagcatgtaggaagagattgcgggtccatgagaacggttcatcaaacttcttactcaattcaggtttaatgagaaatgaagttatgaattctgcatttgtaaagattgacctttcttatggatctactaggctgcaacaaccctttgagttgttgtttctctgttattgggatacatgtgtttagaaaagctaaacctgattacatcagaataaaatcatttggcaagctctccatgtgtcctgtcatgcagttggcagcagaatgttgagtaagaacatcagccatctggttaatcatcagtgcagagaaagtttcactgactgggttgagagaagcaggactttaatacaccttccacacttgcccctacattagttacttcaaaaactaagaaagcattggatggagctcgctggagtgcttgccaaatgtatggagccctggctttgattcacagcactgcaaaaccaaataagaacagcaacaagacaactgtaatgtctaaaactgaactaacaaggcatgaatgaatgtagggatcagatgccactagtttctgccatccgcatcaaggatcgactaaccatttccaaaagagaaaatgctacctttattaccagtggcctgttaaaaactacaaagaaatataatcaccaagataaccatcttctttaacatacgtgttttattttagagctggttgaacttcagcagtaccttgaacttatttttatcctgtatatattgtctttttattttaggagccgttcagtccaatgcattgatagttaatttgacagattcaaagggtacttgcctttatgcagaatgggagatgaatttcacaataacatatgaaactacaaaccaaaccaatgtaagtaaacatatctgcctttttttaaggtttatttgtttattatatgcacactgttttgcctgaatgggtgtctgtgcaccatattcatacactgccctgagaggtcagtagagagcattgaatcccgtggaactggaattacagatgagggtgagctgccaagttggtgctgggaatcctctggaagagcagctgatgctgttagccagggagcagcttttctagtccactagcgtgagcagtttttgtttgttttgtttgtttgggtggtgatggtactggggtttgagcccagggtctagtaggtgctaggtaagcactgtactacttccagtcccatatttgtcttgagttttctatcagggatttatttacatttagtattttttgtttgtcccccaccactgcagacagggtctctgtgtagctctggctgtcctggaactaagattctcctctctcagcctcctaagtgctgagattaaaggtgtatgcaaccacgtccagctttacatttaaacctttttttataccctttactttttaaaaaaaaaaattttattgtgtatgcctgtaggccagaagagggcatcagatctcattacagatggttgtgagtcaccatggggttgctgggaattgaactcaggacctctgaaagagcagtcagtgctcttaacctctgagacagctctccagcccctactttttaaccttttatactgggtacaaataagatctcaggttaatttgcctaacacttaagtcctgtgttgtaaaagtattcatattcttacaagtattctgcttaagattgcagaggatactttgaattcattgagtcttcggagttttccttggagatggttatatgcaaatgaggaaaggcttcatagtatttcagttacaacaacttttgtaataatagaatatactgacattaaaacttgaatttctttttttcatctttaaaaattatgttttaatttttttatgtgtgtatagggtgtatgggtttacatttttttctttgtgtgtctgtgtaccacttgtgtgtctggtgtcagaggagggcagaaaagggcatagcctcggctggaactagagttacagacaattgtgagtccataatgtggggtctataaatctaacccaggtcttccagatgagtaagcagtgctcttaaccactgagctatctttttaggctcccaacttctaagaaagagtttgctgtatctacctatcgttttgttggtctctggtcttttttgccttattttcattttgttaaagccctatctataacatgacttaaaaaaaaacgcaatgtgtaagctgtgaaattgttcactggtagaactcttccccattgtgtatgagaccctagattttctcccgaggtttgctgaaatgagctaagagctagagaagaggccgggtgatttctctcttcttcagacatacatgagcttgcccagacaactggtgtgtgtagctaaagaatctgagattggtttttgtagcacaccaagcaaagagcctcttggctaaggggaaagtgtgatttagctaaaagtggtacacccaactgggttctccaggggtgggcttttactgatgtctgttttttttttcttcttacccactcctaaacttttttttttttaacttttagaaaactataaccattgcagtacctgacaaggcgacacacgatggaagcagttgtggggatgaccggaatagtgccaaaataatgatacaatttggattcgctgtctcttgggctgtgaattttaccaaggaagcatctcattattcaattcatgacatcgtgctttcctacaacactagtgatagcacagtatttcctggtgctgtagctaaaggtaaacttaagaattgcatttattttgtattctcttgctttcattagtaacaaaaaatgtcaacaactaaaataatgattattatgcctatctgtcttcctaataagagtgaaaaaaatcaagtcctcttactcatttcagctctgagtccatgcatagaattgtgttgggcacagagtgatattgactatcattctggagtgttttgctctgcagtattttactgagtgtacagtttatagcaaccttgggaagtaaataaaggggatgaggcttggtgacttccccaaaggctctaaatttagtaagtgagcaaagagctagtgattgcagccagtttcctgagatagagagttcgtacactgaactctttttatctactcagtagtgggcactcagtaaatggtggtgagtgagaaaacgccttactaattgaaagtgtccaaggtcagtaatgtagaagaaatgtctcaccttcagatctttttactattactgtgaacctttacttgaaaagtaaatggagataaagttaagctgtttgctattttgagacaacttaggttaagaacaacttgattaagtcagtctcgtcattcttaaacagtcatgaattcaaatgttctgaaagtgtgtgtggtggagaatttaaggtgtttgttttgttttgttttgttttttgtcaagccaggctgacctgagatgtttagtcctcctatcagcgtccttgaggattgagatctttggtgtttgtacaccaccattctcagtttgtgatgctgattttattagcgatgtaattctctccctttttggccccctccagtgatgttagcttagcaggatgtctggagtagttagctctcttacacataatgtgtcattctgactgcagaaacactggcaagtcagcgtcaggcgctgagatctggagttaataccactcttcgacttgtagatggaaagagcatactttaaagccatggagcttcttaagttcaaggccatcctggtctactcaaaaatcaaacccaagttttaattctactaatctcttgttttgagagaagaaccaaggcaagaggttattaacagctattttaagatatttatctttggctggggcatggttgcattgtctttaattactaggataaatttgtccttggctttctgtttaagcgagaagacagtatataaatcttttgtgaccctgtgaggaaaactctagaccttttttctcaacaacctaaagcacacagtaaattcagaatgtagtatgcattttatagttgtatctgtggatccatgggctgagtgcctgctttctgagtgttctcaccaggggtcctgatgagaagcagagagaaaagagggattgggaacagaatgggagaagtcagtgtatgatggaggggtaaaggtgcttgcttcgtcctgagctgcaggcttatggtggaatgaatgtaagtggaagaattggagcaggatgcttccaccagggggagccagaggatgcacagcagtcattggagagttgcctgagattcaggctgtctgtgtagcaggaggccaaccaagccagttggctcagtacagcgggcctcattataggggcctgggaagccaagaaagagcttttagcacgattaaggtaggcactagggaaaccgtggtaggtttttctaagccagaaaatgatacaagcaaagcatagagagtggtttatatattgctggattaatttggaagtgaggagactacaagcagaagaagccagcttcagaatctgttataatagtcgggatgatgtaacccatccctcaaccgcggaggcaactgttaaaggaagatggctttagtcagacatgttagataaaatcactaacatgctaacccctttccttgtctttatt**ataaaca**agtggttgactttttctgtgactgtcttctgaataataaagtatcaaaccttttgaatttaaaatactttctttatgtacactttagagaattgcctttgaaataatttagtagtttgaaatgctacttttgttttgctttgctttgctttgctttgctttgctttgctttgctttgctttgttggtttttctgagacagggtttctttgtacagcctttgctatcgtggcactcagtctagatgatactggccttcaacccacagagatcctcctgcttctgccttcggagtgctgggattaaaggcgtgcaccaccactgccctgctctgaaatgctaagttttaggcatttaataattgaagtaactttgaagctgctacttaaaaaatgtttttatgattaaaatacatattaaaaacccagagagaataatatagcagactcacagagacttattacccagaatatattttttatttttagagtctcattgtatagccctacctagcctggaattccctatggagaccaggttggtttctaatttatagagatctaccagcctctgtctcctgggtgctgggattaaaggagtatgcctgggttaataaatcgaattttaaaatgtttcattatttaaagaaacaaaactttactgataaatttgagatactccttgatttcattttcttctctgcctgctagaggcaaccagccttatatcttcagggtgtgatattcctgtatgtgttcatagggaatagttttaacttatatagtttcatagtataatcctaaaatgtttagaaacctttttttcccatcatcattttttgtttgttttgtttttgaaacagctcaccaagtagctcttgttttccttgaactcaactatgtagactaggactggcctcaaactcagagatccatctgcctttgcttcctgagtgctggaattaaaggcatgtgccacatcgcctggcttccgctcattgttttttaaggtctgtctttatatagcaagttcacatctgttcctttttgataacaaggagacaattttaggaaaacatttaaatttaaaaatccccaaactccatctgtccattagggggtgccaagaagcactacgtggctgccttgttacatgatggagatgcatagaagatgacccatgttagggatcatgaatacattcttttctgcttcgccaagaaaccaggccacttagatctgtatgagttcagtattttgcagtttcactataggcattccagtaaaaattctgttcatagtggcatacatttttacctgaagatttcttgaaatggaatcatttgttttacgtttttcttcattacactttatttttgtctttttgagacagcttctctgtgtaatagccatggctgtcttggaactcactttatagaccaagttagcctcaaactcaactagatctgcctgcctctgcctccagaattaaagattaaaggcattaattaaaggcatgtgtcaccaatgccagtctgggacacagtcaccaggcctgttacttctctgaatcacctcagtagccccagaaagaggaaggctctatcacttctaatgtagcccagatggacctctggctagctgaagccaagcacaagcttgaagtcccaggcctccaggctctctacctctggagcggcaggcattttagtgctggcctatgtgcctccaccaccttattttctggggcctctgatatgcttatagggcagtaactctatccactgtgcaccgttcaaagcagcctgcctttcttacagtcactaagtcccatccagttactgtagaaaggcgttttgaacttgcctttgctttttttcttcctcctgaccttcactatatctcgagtttcacctaagcagccattggccttctgtttcccctggtaattaaaagttagggtcacagttttttttctgcctgtggttggatatttattaacattccttaaaataaattaggccttatttcctctcttaaggcttaccctaagtaggattggtatctgttggtaaaggagatcatttttctccagcaaaaatggactcccttgtgttgttgttgtatgtgtatacaccttctgttttgacactttttgtcttaccgttctttatttgtttcaactctccttgctatgctcttggttattttgagagtgagggtgtagggagaggaaggaacatgaagttgggtgggtagagagggtcagggacggattgggggagggggaacatcatgactttgtgtgtaggagtgtgtgctgtcaagtctgacaaccccagttggctgtctggaatctgaaggagagaaccatctcctgcagtttatcctctgaccccccccccccacacacacgtgtactctccataaccagcttgcacacaagtgtccatgcacacaaaaccataacaagagcaacagatattgtgaaaaacagaagcagttacagtgaatttgtcattcctgtcaatgctccacagtttattacctttgacatggcaaggcctagaagaaaagggaacctgtattttgttaatataaatatattagtttctcttgtcttcctggaaatatcattgttaaccttgttttataggagttcatactgttaaaaatcctgagaatttcaaagttccattggatgtcatctttaagtgcaatagtgttttaacttacaacctgactcctgtcgttcagaaatattggggtattcacctgcaagcttttgtccaaaatggtacagtgagtaaaaatggtaagtaagttccttccttccttccttccttccttccttccttccttcacacagggtttctccgtataacagcccaggctgtcctgtaactctctgtagatcaggctggcctcggactacgagagatctacctgcctctgcctctctagtgctgggattaaagacattcgcttccacggcctgaccaaaaaccatgagtccttaaatggacttgtatatggggctcttattttaagggcagactatatcaggtagcattcactgagatcattgtctgagaacatttcttttttttttttctgttgttttgttttgttttgttttgtttttttcattttttttattaggtattttcctcatttacattttcaatgctatcccaaaggtcccccatacccacccccctaatcccctacccacccactccccctttttggccctggcgttcccctgtactgggacatataaagtttgcaagtccaatgggtctctctttccagtgatggccgactaggccatcttttgatacatatgcagctagagtcaagagctccggggtactggttagttcataatgttgttccacctatagggttgcagatcactttagctccttggctactttctctagctcctccattgggagccttgtgatccatccattagctgactgtgagcatccacttctgtgtttgctaggtcccggcatagtctcacaagagacagctacttctgggtcctttcaataaaatcttgctagtgtatgcaatggtgtcagcgtttggatgctgattatgggatggatccctggatatggcagtctctacatggtccatcctttcatctcagctccaaactttgtctttgtaactccttctatgggtgttttgttcccatttctaagaaagggtagagtgtccacactttggtcttcgttcttcttgaatttcatgcgtttggcaagctgtatcttatatcttgggtatcctaggttttgggctaatatccacttatcagtgagtacatgttgtgtgagttcctttgtgaatgtgttacctcactcaggatgatgccctccaggtccatccatttggctaggaatttcataaattcattctttttaatagctgagtagtactccattgtgtagatgtaccacattttctgtatccattcctctgttgaggggcatctaggttctttccagcttctggctattataaataaggctgctatgaacatagtggagcatgtgtccttcttacctgttggggcatcttctggatatatgcccaggagaggtattgcgggatcctccggtagtactatgtccaattttctgaggaactgccaaactgatttccagagtggttgtataagcctgcaatcccaccaataatggaggagtgttcctctttctccacatcctcgccagcatctgctgtcacctgaatttttgatcttagacattctgactggtgtgaggtggaatctcagggttgttttgatttgcatttccctgatgattaaggatgttgaacattttttcaagtgcttctcagccactgagaacatttcttaaggtgcagactaaatatttttaaagtgatctatcttccttcctcccttccttccttcctcccttcctcccttccttccttccttccttccttcctccctccctccctccctcccttccttccttccttccttccttccttccttcctcccttccttccttcttttcttcctcccttccttccttccttccttcctttcttccttcccccctctctttctttctttctttctttctttctttctttctttctttctttctttctttctttctttctttctttttttgtgagacagggtttctctgtgtagtcctggccatcctggaactcactctgtagatcaggctggcctcctatctcagagatctacctgcctctgcctcctaggatcataggcgtgcactgtgagcataggcttaaagtgataaccattcttacagataatttcatttgaaattttatgatatacatgcaaatgtcttgtttctaatatggatggtgtgtgtgaatgtgtgtgtgtgtatctgtatgagagaggaggtgggagggagggagggagggagggagggagggagggagggagggagggagagagagagtgtgtgtgaatgagaatgtaagcactagagacccacagtggactgtttaatggaaatgtaataatttcatattcattactagaaaactaatacttcctaagaagggatttaaataccaataacctgctagaggagtgatagaaataaaagattcctttgtatttaaacccttcaacgatttcaaggctagtttttagtaccacttcttttttttattattatttttttatttttaagatttatttattattatacataagtacactgtagctgacttcagacacaccagaagagggtgtcagatttcattacgggtggttgtgagccaccatgtggttgctgggatttgaattcaggaccttcagtagagcagtctgtgctcttagccactgagcaatctcgccagccctttttatttttttaaaggtatgaagactggcacaagaatgattttttttttaagatttatttatttatgtggttgctgggatttgaactcagggcctttggatgagcagtcagtgctcttaaccactaaaccatctctccagccccttttaaaggagcttttttttttttttttttttttttttggtttttcaacacagggtttctctgtgtagccctggctgtcctggaactcactctgtagaccaggctggcctcgaactcagaaatctgccagcctctgcctcccaagtgctgggattaaaggcgtgcgccaccaccgcccttagtaccacttcttcacagtggttgttacatttgaaggaaaaaaaaaacatttttatcttgggggtggggggtggggttctttttagacttgtctctgaatttctcatcagagacaggggaaattctttgaaataaaggcatttgtgggccctagaaggaagattacgtggccacagttgaatagctataacttttgatgtgttccgagtttgtctattctggtcacaactcataagagattatctgtttatgaaacagtagtttaggaacctgtgcttcatccactttgaaaatagaccaccgaggtcttctttgctctaattaacccccttggactgtga**gaagctaactcaaagtagtttgctcttccctggccaaataaagcaagatcaccctagcttgttttgtcctttgttttagaacaagtgtgtgaagaagaccaaactcccaccactgt**ggcacccatcattcacaccactgccccgtcgactacaactacactcactccaacttcaacacccactccaactccaactccaactccaaccgttggaaactacagcattagaaatggcaatactacctgtctgctggctaccatggggctgcagctgaacatcactgaggagaaggtagggtttagtgaaaccttttttgtaaattacatcttaagtaatgtatagtgtgccatgcacttaataaccgatactagactgatttaagtttaaatgcagagctggtgttgacttgagttttgaataatttggaatgagtatatccatgaatagttaatatcaccatctaggaatcttgaccagcagaaaacctgccaatttactaaaagacttctttacagcttagattttataagccttcagtagctcagcaatgtagttaagttggagatgggagaagataaagttcagggatagagcagttacatcccatgcacaaggtctgggcaccaccccaacactgccaaaaatgaaaggcaaaccaaagcctttgcaggcttttatttcaaccacactgtaggttggtgcaaggctatttgatgagtcagagcacgattatggtcaaggccttgtccagttgcaatacctcatttggacaccagagggagtgccagtgaacaaatctcatttttcaggatcctgtgattgaaacaagccagacagatggcttttgtgctctgaaaaaaaaaagtgttttcttttgactcattactgaggaaacttccctcttgttattgtagttgaagttggctgagtcataggttgaagtcttgcatcaagttctcatgaaagtaagacatcaggaattcaagtgtccacacaaaagaggttgtgttgtatgttgacattggttttatcagtgtaaagggctttggtgttgacttttacagttaatctgcacctccgttctctttattctcccgaaggtgcctttcatttttaacatcaaccctgccacaaccaacttcaccggcagctgtcaacctcaaagtgctcaacttaggctgaacaacagccaaattaagtatcttgactttatctttgctgtggtgagtactgactgttttctaaagctaagcgctttctctgagaagtgtacctgcaagtttgaacagtggtaggtgtatgcggggagggggacgatatttgctttacttatccctgctcatcttggagtcaactgggctatctatggactggactcttgtattcataatcacacaaagtggccattgtaccattatatgtacttcagtgtagaaacaagaatgaactcacagtaaaagaaaactaactatagttagtttttatattattttctctattattagacaattctcatgctaacacaccattttaaaccttttatagttatatcctttgtttgtaaagatacatttattatgtgcattggcgttttgcctgtatgcatatgtgagtgaaggcgccggatgccatgaactggaatcacagatggctgtaagctgccatgtgggtgctgggaattgaaccccgggtccttgaagagcagctagtactcccaatggctgagccatcttttttagccctcttaacattctttatagtatctgtattagtaatagccagcattaatgaacatg**gttagctgtctatttctctacgccctacatacaaccttgaagtagtgttaggtaaaaatgtctcaagttcagtatctgtggcagacattcttgcttgtaatacacaagaccctgagattgatctccagaaccacaaaaattatatttattgtgaacatgcacagttttgtttttgttatttaaatataaacaatacactatttttatacattta**tatttaaggattataggtaatttagggatgactgtaagtatacaggagccacatgtaaattctaagcaaatagtacaccattttgaaagggggacttggacttcctagattttggtatccgggtactaatcctttgagaatctaaggaacagttcccttgttatctatgtgtaagtccatccattgactcattaaattagttgtgattgtgatgttctggggttccatcgtcagcactgtaaaaattacaaaataaagaaaaaaagaattagcctcttcaatgtgtccaggacagacctgccttcccttttctcttgctctccctctcattctctttttctctctcaattgggattgaattcagagcctcacagatgctaaagaagcatgcagctacctggctatatgcctagtgccaggccagatgttaacacaaagaggaagtgaagagcttttagttctgaaggtttaatggaaaagatgacatttgagggcattcagggataggtcagatctgtacatacagaggtgaggagaagtagaaaagtagaggttgtccgtggatgaaggaacataatcagtgccagctgggaggacgccgacgagctgtgtgtccagctgagaatatgaagggagttagaacaaagttaggagtgcatcttgagtgccaggcagagcattgtctgaacagaggggagggtatagtaagaggacacatcaagttctccattacacttgctgctgaaatgtctctcatttcattctgctctctctgcctcatgtctgtttccagccatggtttctctcttctgggtggttcaaaggcctcttacctaagctccccctttgtttatctcccctagattaatcttcccaaagcacagctcttcccataaatcttcagtgattgtgttttgccaacaagctaacatctaaaggacagaagggcagctcaagccctttcctccacgcagtgaactctgctctcgcagcttggatttcactccatgaaccttccttctctctgctttcatgcaatagcttgtgatgtctaagagcacagcagtaaccagtccgtatgtttggagccagactttgggtttcaattccagctcactgattgctaattggcagcagtgggggaggccacttagcaactctgtgcttgagtgactcatcctgaactgggcatagtaacagcatgagtagtatagggtgttggagccaaggctggtggcacagctctatactcccagctacttgggaggttgagggaagaagattgcaaattcagggtcagtttagttgagttgagtttagtactagtctgggcaacttagtgagaccctgtctcaaaatagcaaacataatgactgaggacatacagctcagtgtcaggatgcttgtccagtgcctgcaaaacgctagggtcaatttccagtgtctcacacccagaaagaataatggctagcacttagttctcagttgttatttaggtaatgtttctttgtgattgattacctttgcttttgctcttgccatcttgaacatatccttgatccacttctttcctaaccagatcccgcccacccccaattccccacccctcagctgacatgagggatgcatcttcccttattggagatgattttcttgccaaatttctaattggccactttcgttttttaaagacaggattaacttatttgtgtatctttttttttcaagtcaaggaacaagcgggttacaactccaggactcataaatgctgagcaagccctccaccactgagctgtatctttaatttcttatggatctatgtttcctcgcagattgcaagcactgggagggcaagatacaagtcccatggagcagacatgttactttgtgtatactaagcctgcagacaattagaatgactggctgatcagttaaatgctttagaaagatactacggaatgtaggctaggtagagggacagtaaatgttccatactataatagaatgactagttcaaaataccctatgttgtattttatgaagatctagaaaaggggacttcagtgcttcaaatacaaacagttcagagccgggcgtggtggcgcaagcctttaatcccagcactcgggaggcagaggcaggcggatttctgagttcgaggccagcctggtctacaaagtgagctccaggacagccagggctatacagagaaaccctgtctcaaaaaaccaaaaaaaaaaaaaaatacaaacagttctaaggcaagatgaatgccaattgccctttatttgattagtatttggtgtatgcatgtgtcagaatatcacactgagctcgattaatacgtataattaagtactgggcatggtagtacagacctttaatcctagcacttaggaggcagaggcaggtggatttctgtgagttgtaggctagcttggtctacattgtgagttccagaatagtcctggctatgtcagaaaaaccctcttttgaaaacaaaatacattcaattaacatatttaatgggatgtggtagtgcaaggctttgatccagcacttgggagatagaggcagggggattgctgagttgagctagcctagtctatatagtaagttccagcttatcaaaggctacatagtgagatactgtttcaaaataaaagtaaaatatatgttcataaaaaaaataaacttccaaacacttgggaggcagaggcatgtgagtctctgagtgtgaaaaagttccaggacagccagagggtatataacagagagaccctgcctctaataaacagacagacagaaaggcagacagacggacagataaaagcttggcaacatgggtcacttgctgccaagtctgatgatggacttgagttttcattcttagaagcaacatgattgagggagagagctatgagttgtcttccgatctccatacacgtttatgcatgtatacacatgaaaactttaataaacattggtctgatgatgccttcaagcctgttacatgcaataagccttgggtcctagacccaaaccacaaagtttagaaaacaaaacaaaatgtaaaggtattaaaagatcaggatggggggctggtgagatggctcagtggttaagagcaacgactgctcttccaaagttcctgagttcaaatcccagcaaccacatggtggctcacaaccatctgtaacaaaatctgacgccctcttctggagtgtctgaagacagctaaagtgtacttacatataaatcaatcaatcaatcattaaaaaaaaagatcaggatgatgagccaacaaaagagctaatagtagtgaaaattagagagctgggtgggttaaaagatcacatggtgagctggccatggtggcacttgccttttgttttccagtaagttattttttattcactttacatcctgatagtagcaccccttcctcctgtcttcctagtcctacccttacaaatccttccctctattaccccctccccttcacctcaaacccctgccttgggtaccaccccatcctgggccatctagtctgagcgggactaaatgcatcatctttcactgagtcggaaccaggcagtccaggtaggggaaggggatccaatggcaggcaacagagtcagagacagtccccactccagttgtgctgggattcacatggagaccaagctgcacatgtgctttaaatgtgtagggggcctggggccagcccatgcatgctctttggttggtggttcagctttgtgagcaccagccccttggcccaggttagttgaatctctaggtcttcttgtcgtgtccttcaaccctctggctcactcagtactactccctactctttcacaagactcctgagctctgactgatgtttggctgtgggtctctgcatctgttttcatctgctgctgcatgaagcctcttgggaaacagttatgctaggctcctgtctgccagcagagcagataatcattaatagtgtcaggggatggctctttcacatgggataagtcattcattggccattccctcaatctctgctccatctttgtccctgcacatcttataggcaggacaaagtgagtacataccatgttcatctctctgggtctgggtggcctcactcagggtgatattctcaagttccatccatgtgcctgcaaatgtcatgatctctttgttttagctgtctttgttttagttgaacagaattcagtagtgtacatgtactcatttgggcagaggcaggaggatttctgtgttccaggccagcctgatctacagagctgagtaccaggacagccagggttatgcagagaaaccctgtctcaaaaaaagcaaaaaaacaaaacaaacaaacaaacaaaaaacaaaaccaaaatggttacattgtgacctaggctagctaatacggactcagcaggctgcaaaaagaatatgacaggagaggatgaattagcctatttccaaaacgcctgactgctgcagtcagagtttagaaatgaaatgcattggaaaatagattcgtgacaattagtgttgagattttgttgtagttatg**ctgctggcctaggtcttatggtttgtttcctcatctgtataatggaactagattcctattctgtagataccaactttttctttaggcagttgtgatatgttattaactagaagctctttttcaaacagaaaaatgaaaaacggttctatctgaaggaag**tgaatgtctacatgtatttggctaatggctcaggtaagcacagcattggcttggaggagaagagtggattttatggagggaaaagaaatactaattagcttatggccagattgtgtcttatgaaatagtagatgctatgtaaaaatatttcttgatcacagcttaaaatctaatgtagaactacagtgttgtctcccttgcagaatcatcttttcaaagaatggggggtgtttgttagggaagcgactagattttaggctactgagagctctatggaaagattcgagagaagtgaatgaggtggtgcatgcctgggatcccagccttgggaagcagagacaggaggccagcctgggttacacagtgagccctaggtcagttgggtctatatagtcatatccttcctgtctcagaaaacaaaacaaacaagtgaatttattaatgaaaacagttgttttgtttttttttttgggggggtggtgttggtcagagcctgactccaaactcttatactttactaaaacgccctgaggaagggctagggagatagctcagttgttcaagtctttgcagagcacaaagatctgagtaaaaaaacaaaccaaaaggtaggtatggcgatgtgcacttgtgatttcagtgctggggaggcagagccaggcggatctctggagcaagctcaccagctagtctagcttatttggcagctttaagccaatgaaagaccctgtcttcaaaaccaagatggacagcatatgaggaacagtactcaaggttgtcctctggccaaccccccacatgcatgcacgcatttacacacacacacacacacacacacacacacacacacacacacacacagcccctccacctacacacatgagacacaaccctaatgactgccttatttttcttgtgttgatacactaagaagttactaacctgccttttttttttcctgcagctttcaacatttccaacaagaaccttagcttctgggatgcccctctgggaagttcttatatgtgcaacaaagagcaggtgctttctgtgtctagagcgtttcagatcaacacctttaacctaaaggtgcaaccttttaatgtgacaaaaggacagtattctacaggtaagaaacaaaccttactcactccgcccttctgtcaccctataggtggcttttcctctgactcaagaggattctgtgcatgaacatagagaaacaaaaatgcccctcttgattctgatagaaagttcttacagcctggaaccaattgtttaactcttcatttggctcatcattttgctcaaagcagatacagtttttgaaaacctgaaaataatttgaagtatttaagaatcctttttatggaacaggtactgccctggggttgaatttgggtggggatggcacttggagcagcagctttaagtaccttacatctgccttcttgggaccatcttcgggaacattttcatacccatatgaaaacaatttggcttgatttttcttaagacccaagatacagagtcatagcaaagcctcacacccgtgaattcatcttgctagccagagtgtcatatagggagatctgcaagtacaatttagttcaaataaggtgttttgaagtcctactttagaagggcaaatgatggccttcatttgggttttaatgaaatcctcatgtagagttcattatactgtatttgtatggatacaaaagatgtatttggcattgtaagattaagatttgcacgacagaaagaaaagtagagcaaattccttaagtgacttatgcttttaaaccaattacttaaattggcagtttgtggagcagagctttgctgagcagagctttccagtcattgccatcctttcaaacaattaatccagaggcttataagtaaacagagatgctatgtttaaagtgaatatactgtgaatgattcggctgcctttgcagtgctagcaaatactaccttggctttgctcaggccaaaagagacctttaccattcttgtgggtagaaaggttaatcgcaacttgaaaaaatatccaagactccatttcctttgccagaagtggtgacacgtgcttaaaaacatagtactcagctggtcaaggcagagtcatctggggttcaaggtcatctttggctgaaaagtcacttccaggctaacctggagtgtatgagatcctgtttcaaagaaacaaaaacagaatccaattcctttctgtgtttgtttccttataactttaattgttttgatcttacagtagatttatttttgaaattgtctagctcccctacatcattatcctaattttaggtctgattttgtgtttaaaatgtaaaatcattcttactactatttgcagttcaggtctgttttatctagttggcagtggcctgagatgagccaatagtctttgcctaatttgaaagcttgggtgccagaaagatatataacatttaatactgatattgaatgcatgatctatttatggaactaggttcttggttgtgggtaactataaatatcacatccaggtaatgcttaagtgtattcagggaggaaatagaattgcagtcaggttggagtgttgattggattttattagatcagactacttgctataaatagaaaaaaatagcccctttgggctgctattctgagctagattgataaaacatgtagtaaacacactcaaattagtaagaggtcctagtttcttagctcttccacccactcaaatcacatattttatgccagggacagaagctttttgtgaaaccgttccaacagtgatttctcaagctttgaggtcatctcgtctgtgtggcaactaactgtacccctgcctctgtagcttgggaacctccctaggcagttcatacagatgagtgtggctgtgttctagtaaaactttaaggatatgaagatttgaatttcatgtaactcccacgtttcacaaaagactgtctttttttccagtcttttaaaattatttatgttatttttagctctttagccaggtttggcacttgggctatagtttgctgacccctattgttaatgaactccagggccagcctgattgttcagtccagtaataccctaatttctatttccccagagagagagatacactctcccacttagatgttaaaatggttctatagacataggcttggttagatcttttcatttatgataaagtaattttctcatcttagaagtatatgtaacacactgagtgcatatgatgtgataacattgtttatttagagctataaataaatctggattattcacacaaataaatatcttc**atatgtattcacaaatgaaaggacaaatacactttaagattgtagctttaactggtcatcactttctatactttatcataaaaatatgcctgcctttgttgaaatttcttttataagagcatttctgacttttaaatcagcatcttttacttttggatatttctttttgattatactcattatttgttacacactttttctcacctacagcc**caggagtgttcgctggatgatgacaccattctaataccaattatagttggtgctggtctttcaggcttgattatcgttatagtgattgcttacctaattggcagaagaaagacctatgctggatatcagactctgtaacactaatccacatgtgatctgttacaaaacaataaagcaagtacaagttctcgcatgcaatactattgaaggtacattagttacagtcctgctcttagattgggtggtatagtagagctcaaattaaaacaaaagaattccaaatactatcaactacaaatgaatcatatgattttggttttcctgactaaggagtttaaaattactatggcaaaaggcatgtacaaaattatctggttctgtttcactggtctggaatttctgaatgagaacgtccagtgtaggaaaaagccggtttcttctgcctgcatgaaattgagtttggccatcctgatttccagtggatttgtacttgctccttttgcattgcttgtgacttttgtttgcaggttgacttagctacttcggccttgttccatatttgacctatgaaaaaggtatcagtagatttgaacagagcccagaggtaccatgctcttgccagtaaaattcttttcccatgtttttgaatacatttgtatttaatgtggctgaaatgacaaaaacaaaggtcctaaattttagaatagatgttatctttttatttaatctttaaaaaaaaaaactggatgtttcaatcttttaaattgcaagacacaaggcaattccaactgggcatttcaatctggttt**taggtgctttagagataattgctaggcagtgccaaatgaaggcattagtgctttgtacccagaaattggcctctacatgcagcactaagattaatgcagatttctcttgaaccttccagtctt**ccttgttggtggtaatgtgctttgttctttcctttttttcctcaagaagaaatgttagtatgtcttagaacctaggtaacgaagtgcacttatgctcataaaattgcttgcacctaatccaaaagtcatggtcttgccttagtccattaacatggtcttcatttccagtttgcattgactgctacagcacttactaatttggcttttatgtttaaatggttctgtgataatcaaaacttaaatagttactgttcgttcattgtggtacttttgtattcagttctgtactcagagatggcaccaattgttcatttactatgcaccacctactgcttatataagagaactaataactctttcagcccgatacttaacacatcatgtattccttgcagcttctaggacatcttccatacatgtatggatgtgtacatacatatacatgtgtacatactggtatggcccagcacatatttcttaagctctgcagccatatatagaaggtccttgttatttggggaggacagattaaatgcctttcattcacaagaaaatgaagagctctctgggttccaaaatgctgatgttgaatacatgatatatatgcacacatataggcacttgcatgttagcatgttcatgtctgtgactgtcttttgagttatataggagtggcagagattgtgtcactttaacttgttgtcgacttagcctgaaaaccttactagttcagaatcatactcctgactgtcttctggagcaacagggaactgcatggccaacttaaagtacttgttataaatgaaccagaaatctgattttaaaatacatttttcattttaattgtttttgcattttgttttgttaatccttaaaggatggattgtgcttttaagaatgtaagtgttatgtatttgtcatgaatcaacaaaacagcttttaaaaaagactaattggaacaagtgggtggcactggcttaatgctgttaatgtttctaaacgattttacatttagattatatatcggattcatattgagatacctccaaagcactgttttatagaaagatctgacttcaataaatatttttgtattttacatgggccagtttatgtaccgctatttacactattatttcctatagacaatacacactgtctttgtagtttttgtatttttgttttgttttactggttttgcttcttgatggtaatatactctgtctggtgtgggatattttccacactttagaatttgtataagaaactggtccatgtaagtactttccatgttttctcttcaaatgtttaaagtgctagctgatgtacgtacgatatctcttctcagatatttgcctgtctgtttgcccaaaattgcttctaaatcaataaagattcttttatttcttaaggaaatttgctcacattttttcccactgcttaaaagtgaagattttgtatattacatgatggcaattaaaaaaaaacttagaagtgcttaagaatagaaagagctagattgtttccagtatatgttgtctgtatgctactcagtcgaaagaaagtcccatttgtagggtttgagtgacacaaggatggctatggctgcagaataagaattacctgaggcatgtatctttacctaggcctcaggtctggatgactttatttggcaagtctgttataaggagagagagagacagagagagacagagagagagacagagacagagacagagacagagtgagtgagtgagtgagtgtgtgtgtgtaagaaaaagcactcctgtggaatctaactcacaagtaaatagtaatggaggatatttatttcatagtcaaagtttcagagcatcttcttggtacacttttcttaattcccaaattgagactaacatttcatatccataacacaaactgtctcttaaattagctcacacttgcttttcaatccccatgtaatttgccagtcatattagtatgttttgcaagacaatgttgcagttcacctggcaggtttcccctgagatcctgaggtaagccatggcactgcagttatcacggttctcaatgccactggtagctactgtgggagactttgtccaactttctgaacttctttaggatgttagtctcacagctttcttcatctagtgattgtggggactttaaaattctgctttttcttacctctttgagtaatgcctttcatttcagaaaacttaggcacacttttgagtcttttagaaagagtcttctaagaagccacatgttttatttcaaacacatacttaaactctatgaataatgtaccattttacattgcctgcatattgtcaggaagaattcttagaagattcatctattttgaatattataacattaccatctgtttgtttaggtagataaagttacattgtcttgggttcacgtctcttctactcatctccctttcttctacctagaaaccaaaatcctgtcagtaatcattatgtcttgtctttgaaatttgtgtagacatatatagattatactgaatagcttcaaatattttctgtatacacatgcatgtacttgtgtgtgtgtatactgtgtgtacctgtaaaggctacacatctatgttagggtttttgtgtgctttccaccttatatttgattttttttttgagtcagggtctttattaaggtggagcttgttagttttgctagaccgactggccagagtgctcgttggctccagtgacctccttcccccagtgctgccctgggattgcaggtgcacacagtggatgcttggtgtctgagcttaggtcctcatgcttacacaacatgcatgcactttacccactgagctggctcttcagtgtcagcttcaagtatctgttcatcttcatgtgatagatacacttgagatgtttggaatgtagatctaaaagaaatctaagtacggtgttacattataggtgagtcaccaatttactgtttaaaaagcaatcttaattagtttagtttggttttttttgtttgttttttttctgtgtgtgtttttttttgtttttgtttttgtttttttggttttttgagacagggttactctgtatagccctggctgtcctggaactcactttgtagaccaggctggcctcgaactcagaaatccccctgcctctgcctcccgagtgctgggattaaaggcatgcgccaccacgcccagccttaattagtttttaagagaagttttatatttctttttttattagatattttctttatatacatttcaaatgctatcccgaaagttccctataccctccccccccacgccctgctcccctacccacttactcccacttggccctggcattcccctgtactggggcatataaagtttgcaataccaagggcctctcttcccagtgatggccgactaggccatcttctgctacatatgcagctagagacacgggctctgggaatggttaattaatattgttgttccacctatagggttggagaccccttcagcttcttgggtgctttctctagcttctccattgggggccctgtattccatccaatagatgactgtgagcatccacttctgtgtttgccaggcactggcatagcctcataggagacagctataccagggtcccttcagcaaaatcttgctggcatatgcaatagtgtctgggtttggtggctgattatgggatgatccctgggtggggtagtctctggatagtccatcctttcctcttagctccaaaccttgtttctataactcctttgatgggtattttgtttcctattctaaggaggaatgaagtatccacccattggtcttccctcttcttgattttcttgtgttttgcaaattgtatcctgggtgttctatatttctgggctagtgtccatttatcagtgagtgaatatctaatgacttcttttgtgattgggttacctcactaaggatgatatcctccagatacatccatttgtccaagaatttcatgaatccattgtttttaatagctgagtagtactccattgtgtaaatgtaccacattttctgtatccattcttctgttgagggacaataatattcaaataatattcataatataatataatataaatatacctcattttctgtatccattcttctgttgaggggcatctgggttctttccagcttctggctattaaaaataaggctgctatgagcatagtggagcatgtgttcttattaccagttagaacttcttcttggtatatgcccaggagaggtattgctgaatcttccattagtattctgtccaattttctgaggaacctccagactgacttccagagtggttgtataagcttgcaatcccatcagcaatggagaagtgttcctctttctccacatccttgccagcatctgctgtcacctgaatttttgatcttagccattctgactggtgtgaggtggaattttaaggttgttttgatttgcatttccctgaagattaatgatgttgaacatattttcaggtgcttctcagccattcagtattcctcagtcgagaatttttaatggggttatttgaatttctagagttcagcttcttgagctctttgtatatattggatattagtcccctatcagatttaggattggtaaaaatccttgctcaatctgttggtggtgtcttttgccgtacatacagaagctttgcaattttatgaggtcccagttgtcgattcttcatgttacattacaagccattgctattctgtttaggaatttttcccctctacccatatcttctaggcatttccctactttctcctctataagtttcagtgtctctggttttatgtggagttccttaatccgcttagatttgatcttagtacaaggagataagaatggatcaattcgcattcttctgtatgatagctgccagttgtgccagcactatttgttgaaaatgctgtcttttttccactggttaggttttagctcccttgtcaaagatcaagtgaccatagatgtatggattcatttctgggtcttcaattctattccattgatctacctgtctgtcattgtaccagtaccatgcaacttttatcacagttgctctgtagtacagcttaatgtcaggcatggtgattccaccagaggttcttttattattgagaatagtttttgctatcctaggttttttttttattattccagatgaatttgcaaattgccctttctatctcagtgaagaattgagttggaattttgaaggggattgcattgaatctgtagattgctttcggcaagatagccatttttactatatcaatcctgccaatcatgagcatgggagatctttccatcttctgagatctttgatttctttcttcagagacttgaagtttttatcatacagatctttcacttccttagttagagtcacaccaaggtattttgtattatttgtgacta**ttgtgaagtgtgttgttttcctaatttctttctcagtctgtttatcctttgtgtagagaaaggc**cattggttttttttttttttgtgtgtgtgttaattttatatctagctactgcactgaagctgtttatcaggtttaggagttatctggtagaatttttggggccacttatatatactgtcatatcatctgcaaatagtggtattttgacttcttcctttgaaacttgtatctccttgatgtccttctgttgtcaaatttctctggctagaacttccagtactatattgaataggtagggagaaagtgggcagccttgtctagtccctgattttagtgggattgcttcgagtttctctccatttagtttgatgttggctactggtttgctgtattttgcttttgttatgtttaggtatgggccttgaattcctaatctttccaagattttttatcctgaatgggtgttggattttgtcaaatgctttctcagcatctaaggaaataatcatgtggtttttgtctttgagtttgtttatatagtggattatgttgatggatttccatatattaaactgtccccgcattcctgggatgaagccgacttggtcatgatggatgatcattttgatgtgttcttggatttggtttgtgaggattttattgagtatttttgcatcgatattcataagggaaattggtctgaagttctctatctttgttggatctttgtgtggtttcggtatcagagtaattgtggcttcatagaataaagtgggtagagtaccttctgtttctattttgtggaataatttgagaagaactggaattaaggtgtgatagaactctgcactaaacccatctggtcctgggctttttttttttttttggttgggagactattaatgactgcttctatttctttaggggaaatgggactgtttagatctttaatctgatcctggtttaactttggtacctggtatctgtctag**gaagttgtccatttcatccaggttttctagttttgatgagtatagttgtggtaggatctgatgatgtttatggatttcctcaggtctgttgttatgtctcccttttcatttctgattttgttaattaggatactgtccctgtgccctctagttagcctagctaag**ggtttatctatcttgtttattttctcaaagaaccagctccttgtttggttgattctttgaatagttctttttgtttccacttggttgatttcagccctgagtttgattatttcctgctgtctactcctcttgggtgaatttgctttctttagttctagagcttctaggtgtgctgtcaggctgctagtgtatgctctctctaatttctttttggaggcactctgggctatgagttttcctcttaggactgccttcattgtgtcccattagtttgggtatgttgtggcttcattttcattaaactctaaaacgtctttaatttctttctttatttcatccttgaccaaggaatcattgagtagagtattgttcagtttccacgtgaatgttggctttgtattatttatgttgttattgaaaatcagccttagtctgtggtgatcagataggatgcatgggataatttcagtatttttgtatctgtggaggcctgttttgtgaccaattatatggtcaattttggaggaggtaccatgtggtgctgagaagacggtatatccttttgttttaggataaaatgttctgtagatatctgttaaatccatttgtttcataacttctgttactgtccacttgtctgtttagtttctgtttccaggatctgtccattggtgagagtggggtgttgaactctcccactattattgtttgaggtgcaatgtgtgctttgagctttactaaagtttctttaatcaatgtggctgcccttgcatttggagcatagatattcagaattgagagttcatctttgtagattttacctttgagtatgaagtgcccctcgtcttttttggtaactttgggttggaagtcgattttattcgatattagaatggccatttcagcttgtttctttggaccatttgcttggaaaattgttttccagcctttcattctgaggtagtgtctgtccttttccctgaggtgggtttcctgtaagcaacaaaatgttgggtcctgtttgtgtagccagtctatgtctttttattggggaattgagtccattgatgttaagagaaattaaagagaagtaattgttgctttctgttatttttgttgttaacgttgggattctgttcttgtggctgtcttcgattaggtttgttaaaggatttctttcttgctttttctagggtgcagtttccttccttgtgtcggtgttttccctttagtatcttttgaggggctggattcatggaaagatattgtgtgaatttggttttaacatggaatattttggtttctccatctatggtaattgagagttttctgggtatagtagcctgggctggcatttgtgttctcttagggtctgtataacatctgtccaggatcttctgtctttcatagtctctggtgagaagtctggagtaattctaataggtctgcctttatatgttacttgacctttttcccttactgcttttaatattatatctttatttagtgcatttgttgttctgattattatgtgtcaggaggaatttcttttctggtccagtctatttggagttctgtaggcttcttttatgttcatgggcatctctttctttaggttttggaagttttcttctataattttgttaatatttgctggccctttaagttgaaaatcttcattctcatctactattatctgtaggtttggtcttctcattgtgtcctggatttcctggatgttttgagttaggatctttttgcattttccattttctttgattgttgtgctcatgttttctatggaatcttctgcacctgagattctctcttccatctcttgtattctgttgctgatgctcgaatctatggttcctgatttcttccctagggtttttatctccagtgttgtctcactttgggtttcctttattgtttctacttccctttttaggtcttgtatggttttgttcatttccatcatctgtttggttgtgttttcctgtgtttctttaaggacttgtaactctttagcagtgttctcctgtatttctttgagttattaaagcccttcttgatgtcctctaccagcatcatgagatatgattttaaatctgagtcttgcttttcgggtgtgttggggtatccaggactggctgagttggaagtgctgggttctgaagatggtgagtggtcttggtttctgttagtaagatttttacgtttgcctttcgccatctggtaatctctggagtcagttgttatagttgtctctggttggatcttattcctcctgtgattctgttagcctctgtcagcagacctgggagtacagctctctcctgagtctcagtggtcagaatactctctgcagacaagctctcctcttgcagggaaggtgcacagatatgtggcattcagacccgcctcttggctgaagatgaaggcccgaaacagggccagtcccagaagatgtgttgcctctgcagtctgcatgctcacctgcacagactggtccccaagggacaggggacaaaagatggctctctcaccttctcaggtggtcagagccctccgggcagacacttctcctctggcggggaaggtaccccggatgtctggagcccgaaaatgggtctgtcccagaagctgtgttgcttctgtagtccatactctcacctgtgcagactagtctctgagggacccgggacccaagatggctccctcccctgctccgaaagagccctcccaggtggacacctctcctttggtggggaaagtgcctggatgtgtggagcccaaaaaggggtctgtcccagaagctgtgttgcttctgcagtctgctcactcaccctttgcagtctgcgagctgacctgagcagattggtctctgagggacccggtacccaagatggctccctctgacaggactttttgtttgtttgtttgtttgtttgtttaacagtgtttttattggaactcactctgatatggaactcaaaagattctcgtgcctctgcttcctgagtgctttctgttgctcctgtggtctgacacctgttcacaagggtgggtgtttctcagtgataggacactttcttagcatgtgtcaggccctgagttcaatttgcagtgatggaggtgagggtgcaggggtgggggtggagaatctcgacagtcaggcacaatgctacacactcctttatttgcagcacttgggacataaccaggcagatctctgcaagttcaagcccaacctggaatacatagtgagttttaagtcaaacaagatggcacagtgagaccctgtctcaaacaaaaaaaatctcccaaatacagttttttttctaggaacattttcattgtagtattaagtgtacattagtcaaatagctgctgcactcaaattggcctacataacgagtatttttcttcaggacacactcctattaacatgaactgttttagtggtatagcacatccataatcagcacttggaaggtggagacacatcaggatttctgggtcatccctggccacatagcaaaattgagttccatttggactgcatgagatcctgtttcaaaaacagcagagaacagagtaaaaaaaaactttttttttaaaatcagattttgtttcagttacctacaaattctttcaagttgaatttataaaatcaatagttattataaattagtatttcatatactaattgtgcattttaagtaaatgacagcatctagaaagatctttgtattagtgaattgcctttttgatttgctctgggaatatttgtcttacatgaaagccttattggcagtatttatgaaagtcatctcagtattaggtcaaacacccaatttgggaaagagaggactgttgagactttttgccaaaataagaaattaatctactaatgaattttttaaaatccaaggcataatattttatggtatatgccaccaaatagtttctaggctgggtggtaatgtgggaaattgaaggggcggagttaactgggaactctttagtaccaactgtcctcaagtcaaatccagcatcatagactctctggattcttaatagaacacagtttaaaatgaccatcaatttggcagccaaagccaatgtcaaactttctataaactctaggattgcatcttgttttcaggtacttctccagaggctggcagtcagagtagagcttggtacattttgtgattgcagagcatagtcagcactgcattgcagcctcccctttctttttcttttctctttcctttctctccttttttcctgtatatgagtttttgcctgtgcatatgtatgtgtaccacttgtatgccttttgaataaagaagaaggcatcagatcccctggaacaggagttatgaatagttgtgagcagctaccatgtggatgctgggaatcaaacccaggtcctccgaagaacagctagtgctgctcttattaactctaatcctcttcattcctatcttagcataatcctaataggcttacttaactgtgtaatttaccaacctctggtagagtccaacccctggaagaaaacattaagcaacatgaagcaagggagtgggtaatactagtaagtggtgtttcagcatcctgattctctttgtatgtctttgcaagtgtcttagttctccatccttccctgtagtcatattagacttttcatgatcagttcttctggaatcttatttctcctaggtcctaggaaaccttctatcgaacatcttcaacatgtcattattagccttgctctttaaaacattttttttaactaaatataagtatctcttcaaactggagatggtaataaaagaccctaacttctaaaaacctccaaagtctgctttcataaaggggagctattactttgtgttataatgtataatgtataatactctgtattataatgtataatatataatgctgttcattagccttgttctttttatgaacatttatctcatcttacagacaaatttatctcttccagatgctaccttttcttgaagtccctattctaagcaccccgtgctgtctttttcttaatcttaaatttataaagaagactctcttcttagtccttgatgttgtgactgatcgttagctttcacttgccattcagttgtctctgcagcagtttgactcataactcatcctgcacattgctagatcctataggcattgttttagaattctctaacatgttaaattcttctcatgacaatgggagctctccccttcctgagctgtgacaccatcttctacctgcctttcctacgtctatttttataccttagtccctcaaatattagtattcttaggggttttctctctggtcccttttcagtttactcattccagtttattgatgttttgtttggaaactagatgtcttgttttcttagtgttttaaaaatgctacacagactatttcattatgatggtttcccacacactcattcttgctgctgctttcccctccccctctcctgctttcacgttacatgtattacactccttccttaaaaatccttttctcagtctgtcaatctagtttcatgactacatacaaactaaaatacagtatctgagtttcagaaattggggtattcgtctttgagtctgccttattttgctcaacatgctaatattagtttgatcaatttgacttaaaatggtatgatttcatttttatttatagccaaataaaatgctattgtatatacacaccacattttatctagtcaccctttcatagacatctagactgtttcctactgtgaattagtttactactatgtacaatgcagcagtaaatacagatgtacaggtatttgtggtaggatagacccctttaggtatgtactcacgaatggtatagctaaggacacagacatcttaaattcaaccccaaactgagtatctcccttttctacatacttccttttatgatctatctcacttatcttcagtaactataaatgcaacatgactaaaattcacatttctttcttttggattcccgtcatggtgaagggcaccagcatctgcagaaccactgaagtgtggaactagaaagtatcctgttattgcagcccattacactcctcacattcatttttgccaattttaccttctaaatggatctgtggtctccccattgcacctacacgtcattaacccagttcaggaatattggtcacttggttgttagcgatagaggtggctcctagcttactacttgtatagtcttccatctgcaactctgaagtgtaccctgcttgaattcctgtttgtttgtttgtttgtttttccattgggataagacagaaccactgaagagtgtacttttgtgagctggtcttaaatacactttttctggcttccatcaattgttctatgtcaccttccttggcattgctcctggtgtttttatgttttcttactgtccatgtttttgtctgcattgtattttgtttgcttttatttgttgttactgtttctgtttttatttttttgtttttctctacaggtttctctgtatagccctgtctgtcctggaactcactaggtagatcaggctggccacaaactcagagatccacctgcctctgcttcctgagtgctgggattaaaggtgtgttgcaccgccacctggctctctgcactgtttcttacatctgcagtgcccccactatctgcatagttagttataattaattaatggttaattgtttgtcttagaacacatcaaatgttgcctttccaaagtgtcttatttaactaagctgagttgaccgaatccctgaaatatctccatgattctttgtgtattgcttgccccaccatagcacatcttgaatttgtaactactgatttctgtgtttgactctcacaccagattacatatcttattttttatgttatcatacagcattttattatatcattcaatatatatattgcattatatgttctcatgttcttcttccccagtactttccctgtcctttctttcctcttgctgctttcaggtcatcgaaacctttacctgtatatcacatctgtaccatttccttcctctttagaagctcttttacccctctcacattctcctttgtatttatacacatttaaatctacatataaaaactgatatttgtgttattctggcttattttggttaacataacttcatttgcagtttttccagcaaatgacacaattgcacttttcttcatggctatatatatatacatatatatatatatatatatatatatacacacacacacatatatgcatatatatgcatatatgtgtgtgtgtgtgtgtattcagtttccttatctacttacttgttggacatctgggttaggtcttgatatatcttgtgactagtgctggaataagtgtagatgtgcaagtatctctgaccttgatttcatttgttggatacctactcaggagtgggatagctggcttatatggtaattctattagtaaggcttttgttttgagatactaaattattttctctggatacataagtagtagtggaactcctagattgctatatacatgttttttctttttaatttttcttttcttagattttgtttctttgaaaacttgaaatacctatatattctcatcagaactctctcaccttttattttcctcccattcctatcaacgtgccttcttcctggcttgtcctttttccagattcctctcctcccctcccctcccctcccctcccctcccttcctcctcttcctcctcctccttctccttcttctctctctctctcctccctccctctttctttctttctttctttctttctttctttctttctttctttctttctttctttctttctttctttctttctttctttctttctttcttttttgtttttcgagacagggtttctctgtgttcccctggctgtcctggaactcactctatagaccaggctggcctcaaatttagaaattcgcctgcctctgcctcccaagtgctgggactgaaggtggtgccaccatgcccgacttcttccttccttccttctttctttctttttgaggaaacttgatactgatttctatggttgctaaactagaacattcctaccagtagtgaataaatacccttttttccctcaactacataaacagcatgaagccagcagggccagattaatcatctttccttattactaagtgagctcacattctatgcattgagctctgtgtctgctttatatacgtgtttatttggtgagttagtactctctaatatcgaggattcaatgtcatagttagctctggaagctccaggctgatcaagggaaagcaaatttctgtaagaagcctgggagtgtgtgctcactatacacatttttccccccaatgactgctttttatgaaggggaactgcatagaagtacttgttttttgtttttgcttgaaaaaaaaaaacttttggtgcaacatctcatgaaatatcatatcactactgataatgaatgccaatgacttgccattttgatgtaaaaatctgtatgcagggggaagggttgtcattttaacttaaattgagacatatgaaatgccattgtttctgttctcaattgtcctttctccacatccagctcaagactgcagtgcagatgaagacaacttccttgtgcccatagcggtgggagcagctctgggaggagtacttattctagtgttgctggcttattttattggtctcaagcgccatcatactggatatgagcaattttagtaactgcaatctgattgattatattacagaatacatacaaaaaaacaaagttgtcttacctgtcagtgtatgaagcactttgctccttaagacagacaccttggaactttcattcggaaattagtccccgaagtttgagataagtctttttttgaatttttttttctctgatctgcttaaaatgcagattggctttaaaaagaaaagaaaacaaacacacatatgcaatgttttaaggtctgtcttaagaagctttggccaaattgggatcctaacctaagatgccttaaacttattaacatgaccattataagaaaagatatatggagttgtatcttactggaattaataaacactgcttgaccaccggtgttctgtttcaatttgtaacagctgtcctaatttaaactcaccactactagcttttgcatatacttaaaatacctatcagatattcacagacttggtttttggagggatgaagtagagagtccgtagggccatccacagtttgtttttgttttttgttttttaaataagaactgaagaggaaccagagagtgtttgtttgtttggttttttgaaacagggtttctctgtgtagcccttgtcctggaagtcactctttagaccaggctggccttgaactcacagagatccacctgcttctgcctcctgaatgctgaaattgaggtgtgtgctatcgccacccagctaagtattacatttttaaaggttatgacttgatgtcttttgagttcaagttaaatatgaaattgattctcaaacttgtgatttttttacataggttaaaaacaggctcagaaagttataacagagctcagactggcttcttaattagaaggttttttttgggggggggggtggtgttaaaagaatttgtctgtagctctggctgatctggtagttgctatgttgtccgggctggattcaagctcagatccatctgccttggctggctaaataagggtcacaggcatgtgttaccatgcccatcccgggcttttaagaaaatggttttttttttcagcactacagacggaaggggttcaaacagttaaccgttcccctcgctctcgtcctctttggatgcctggttagcactgaaggatagaggattctatgaactaattaggacagattgtgttgtgtttctctacctcatcttgttgatctctggagcattaaaatctatttagtgttgtcatcagactggtacttatgaaatgtaagctaacagcaatctcagaagggaggcaatgaagcacagcagttatgctcttgcttctccaagacagccgcggtggggcacagctctgagggggtggagaaagaggctgttggcatccaaatgagctaggcagatagtccagattgaaacattttcctggtaaaagtgaagtgctgatggaatacataaataaatcaagggatttaacagatgagaccatgtacacactgaaatgatcattaattcattttaatgtgtatattggcttgctcagagtttctgtcacgtactaattaacagtgaaaatgtgtttaggtatccagctgtcagtgtctgttaaggagctttctgaaaaacagtagtcctccaaaaaaaaaataggctaattgtagttattgaaatcgacattatgttttcagaaccatgacaacgtttgtgtgagtgacaaaagtgtgagagaaaaatctcagataacacaaaacattataatttattcatggctctagccttgtactgaaaagaccttagtgattaggtactccttaagtccagagctgtgtgaaaagggatgtgacttcctcaatgttacatgttcagcagcaatgaaaccgttagtatttggattaaattctcaataaaatgtaatcattcaatagtaaaatgttgattgattaaaacttgagctcctgggtccaaaggtggcattttgagagctgacttgcttcagtcgttttgaaataatgaatgcaggccttctcagtcgtccactaagacacaactgttaggattcctgtgttgtcaaaactcaagcctctttggatcatacacacaggaggaaaagggaaggggctgagtaattgtggctcacttgctgtgacccaggttctttaaaacatattgaattctcaagtactatataatggaagcattcgtggaagggagggaggcccagcttagataacttggcagtttccctaagttagttattacttcagagtctgatttccaattcagttacgtctgggggtcagagtttagtgtccacattgctcatgctgtgctctggtgattccataggacggactcacatgcctttgatgctgctattcttcaccacgaccatcagttagttggaaagaattatttgtaacccgtgagctacctaatgcttatgtggaaaaaacaaaacaaaacattagctggagagattactaagcatatttatttgttggtcttgtggaggacctgagtttgactctgagcacccacctgtaacttcagttctgcggatgagatgccctcttctggcctccagggcactagatgcatgtggtccatagccatacacataagtgaaatacctgcataataagatcaaataaatctttaaaaagctagaacccaccttgatcctgtcttttacttgactagaagcccccaccccacccccatgtgcttggtatggaacccaagagccttaggcatgctagacaagcactctagcactgagctagcttctctttcccacttaacactttaagtctagatgtggcaggggggaaaggaagaggagatgcagaggcagtaatttccctaatgccatcaggcaactctgaagttgtataagcaagtactataactcaatattgtataagcttaattcagagtttgaaccatttcccagtttccctttctgtactgagaattttttttttttttacatgaaaagttaaacagtggtcttgctgtgaaatgttcgggtggatgttgattaagtaatactccaaagaaaatgatagcacgtgtctctgctgtcaatgcttcatgtttgcttcttcctcccattgtattctttactaattggctctctctgctgtctctctgttcttttctaacagctgaggaatgtgctgctgactctgacctcaactttcttattcctgttgcagtgggtgtggccttgggcttccttataattgctgtgtttatatcttacatgattggaagacggaaaagtcgtactggttatcagtctgtctaatcaagccagtctcgtgcttccattggccaaaagccaggaattgctgtgcgataaatcggatccacagactgacttcagtgagttgcaagctggcttgggcatgagtagaactttaaaaatggattaggaccagtctttatttctgccattttcttctctgttcattaagatgtaatttaaagaaaacttacaacttagactgtctttttaaagtacataattttaagcatctggatccaattttggaagcatttctaccgaagtataattttatatgttgcacttagttgtattttgctactttgatgttatttgcaaaatcagaactgaaagaggatgtcacttgcttcccaaaataagttttgaaacgttatagttcatcttctttgaagacaaacataaaacttagctcataaaaggaatgactttctgtaccttctaaggagaattgtttaaatctgtgtaattctgttactttagaaaggggtctcggggctggagagatggctcagtcattgaaggctaggctcacaaccaaaaataaaaagaggtctgggatgtaacccagtgatactgcatgcatatatgaagttctgggttccagcctcacaacttaaaagtagcatctaaaacagactacactcaacagaaaaatctgcctatttctccattactttcagagctactatattttaagaaggaattgtttgcttatttttttggctttggcagtttggggtgtattgattatattaaacctgatatttctactgcaagatgaagcaatactgttcgtgttttttttaaggtgttgatttagaatggcctattttttaagggtataatttagaattgtttcccagctttaacttgttcagaaagttaatttatcctgcactaacaaaaagaaaaacccttctgtacacttttgtgcctcagtgagttcaagaattgatatttctaatgcattgacttttcttctcctggtaccaagtgctaactgtggaatctagtggacgctgacatgtcgtctgtcaggtggctgcctaaccaaagctcacctgggatgatcttacaaatgtattgattttgatagagtcttgtatccctgtcggggccatgagtgcctgtggcttctggagtaaggtcctagacactacgtctccttgtgtttgttggattctttagccactgagttactttgggtgtttaatgctgtgcatgtttatgccccacatgccagctttgcacacctgatactagcacgaggcagatgttgaattttctcgagctattttctctctactgagaactgaagagtccaaagatctgttttcacttcagagattttagttaagcctaaaatagtaatcttaagtccaagtcagtggttttcaacctctggattttgaccccattaacaaacccctgtctccaaaaatatttacattacaatctgtaacagtagcaaaattagttatgaagtggcaacaaaaatgttttgggatggggggggggtcaacagaaagggttgcagcattaggaaggttgaggaccactgggctaagtgatgttagtttgatagcagatcccatgatcaaatcccaatctacctctgagcatactgcagctgctcaccactgtgctgctgccacgagctgatgccaggctattttaccattaactgcagttctgaaatcctattaaaaagaaagcaggtttgccaagatgctttcctgctatcttatctctgtctatctcaaaagcaaagacacccaactgtgtagaattgtgggtaactcaatccctctccacttgctctaatagatacagaacggcttttaacaaaacgtcctccaacggcttctgcttatttgttatttagcaaaagtcatctagccaaaatgccagtactgtaaagaacagtgacttcctttcaatatatattttggatattatcttgaagcctaattaattaaatggactttttggcttgctaagtatatgacttgaatattcagagagactccaaaaatatgggtatattcaaaacctttctactctttgagcaggatactaatgtaactaattcttcattaggtgttttggctttatcttaggcaattagcagatgtgtcttttagtctctgagcattgaaatacaaaatcataggaggttaaaaaagttgcttcaattccagtgaagttcaaagtctgtgaactgagttgagtgttcagttagcttttctttgtgagttcatgttacaccaatataacagtggatcagactaagactttgaacttgtgaatggtgaatcattttctacctaatcctctttgggagaactgatgcttgctgttctttcaagttttggatttgtcccatttaaagaactaatggatccagggaaaataaagtatccttgtttgatctcatatgtgtgattttagattgatacatcagcgctaataaaggtgatacatcccatgccaccatggataacctcacttttattctgtgtacacagccttaagagcttagattgatttatagctttaagtccttttcttttgacatgccagcacaaatcatgatcttaaaggtttgcaatttgactcaagtcccattataaggtaactactagaacctttaaaatagacaattcatggggatggaaagatggctcagtggttaagagcactgtctgctcttccagaggacctgagttcaattcccagcaaccaccattcttaaatgttagcatttcattttgtgactgctccatgttttattgccatccactgtttttttttttcttttctgagacatgatcacatataacccaggctggggctttgaactcctgagcctcctgcttctaactcctgggtactgatattataggcagcactaccacatacaactttagttggaaaagtggctctacacaggatttgtgggactggttagggaaggggagtctcaacagaatatttcttgtttcatctcctttactgaatgtattcacaattcaatacttaacaagtgcagctagcaagaatactccatgtatattgtctgtaagggttcctatggatgtaagtcaatttgaaggatcaccttttaagttcagtgtatgtaatccctgagagcactactgcctttagaactcagaggaaatatttcctcttttccattacttttattgtttatgtatttttaaggcactgtaaatttggatcgctttgtttcaagaggtatgcacaatgaacaacaagttttggtacatagtgtttttgtttgtttttccttgaaatggtttctctgtgtaacactgactgtcctggaacttgctctggcattaacctcacagagatccatctaactctgctggaattaaaagtctgtgttacaactgaccaaatggaaatggcggcatggaaatcaactgccctcccctccccccccaaacacatttttctttttttaaccagcaaagcaactctaatttcttttctcaatacaaagtctcactatgtagctctggatggcctcaaacttccagagattcttctgccccccctcccacccccgcccagtgctgagattaaaggtgtgcaccaccgccacctgatagcaactctagtttcagtgagctgctccagggctaagctcctgccccacattgtgtcattcaggacagtaaatctgaggttgcaaattatcattcccagttcacagaagtgaggtcactgaagcattgaagttagtggccaagcggctatctgagttcaggtttgtctgcctccacagaccataccatttagcattatacgtcctgctcctctaagatgcttcaggcttatagagcatttgccttgtattagtcacagaaatctgacttgtgctctgccagcctgcctttaatcaatccaatacatacactccattattcttgagtttggaagggtagatgagttctgtggcaacgtgccttttgaaagctcagtgccagtttgcagtgtgccttattactgatgctaagctctgaggatctttttttccaagctagcctcatccatttattctgatcacatatcattatcattctgccctcagattcttctgcatcatgaatcatttcgactgtgcattagtgtgtttaagaaactaaatggctctctctctgtcctcgctttccttaatgtcactcctatcccagagtcatcccttggatttaagtgcactgtagctaggacacttggcagtttgagctctgatacacagctcctactcccaaatgtagggcagtgctgtggttatgagtatgcatggccatacccagctagggcttcaaatggggatccttcagcttatacagcaagtgctcttacccactgagccatctcttccgttctgaagtgccatttaaaactggctggacaggtaatgcttcattgacaagatgacagttcaaagagacttgactggggtgggagtgtgagctatgaagggagctatggaacaagctttgcagatagaatgcagagaaagtacaaggacctagagaggaaagcacccttgggggctttggggggggtcaatatggctacaaaggagtagagggatgtactagacaagatatgctacagattatggccagaagtttaaatatgaaggtaagcatctcttgaaactctttcaaaactgccctagatttgggagtacgtacttccttgccagccagtggaccagaaacggcaaacttaaggccagtaaaaaactctgtttttttcaacacctagtgttttattcagatagcagtcccgttacacatggtccaagaacattcaaataagaactcaaatcagaagttaaagattggtcttcaaacatcatagccaacaatgccacgtttgcctatgatctctccgatataaaaccacatccacacctcagtggccaccaaaccattcagcacagcttccttcactgtaaggtgtttgaagctaccagttttagcactttgaatggttttttttttcacactctgaatagctgtagggatttcaccaggggttgggggaaccagctcaaccttggcgtagtgccaaaatgtggccaatcgaggcttcgagtaagtcacggcagcggccaccatcgatggtgccgtctccgtgaagttacagatgagcttggccatggtctaaggtggaaggtatgtctccccgaaccaccggctgaatgtccctctgctttttttgttggttttgttttggtttgtgtatgttattttgttttgatttttaaaaatcttactgttttttttatttgttttgattgagggattggagagagatgagtttggggagtggaaacacgataaaaatgtatgaaaaaaaaaaaagagaaaccctgtttcaagcaaataaggcagaaaatgacagaccaggacacccaatgttgtctgatctttgtttacgcacatggacatgcactagtaatgcatacaatgtgcacatgtgtgtgttcatgttcgtgcacacacacacacacacacacacacacacacacacacacacaacaatattcctatgactttcactccccctaccttgtagaccgaggttaggttcctagcatccacagggcagctcacaaccatctgtaactccagacccaggggatccactcttatgtcatggtcccctttctagtgtcatgaccagtaaccacatccacaaagacataaatgtataaagttaaaatgtgtatttttactgggatccacaggagagaaaatgttttgtaaatgtatgtgatacttttctgctatgccacttaacatgatttctagttctactgatttttctgtaaattttatttctttttgtacagatttattttttgtatgtatttgtgtacccttatgtgttgtgtgtgtgaaggtgagtgtaggtgtctgtggaggtccgaagagggtgttagatccccaggagctgaagttacaggtggttgtgagttcaggttctctggaagagtagctggtactcttaatggttgggcttctctccagccccataatttcatttttaaaaaaaggcaacaaaaatccattgtatatattgaacacttttttctttatccattgatctgattctatttcttagctattatgaagagtacagcaatatacatggatgtgtggtctctgtggtagggtttacagtccattacataaagataaatataaaatataaatataaatatattctccctctttccctctctattctgactgcagtttcacctcccttcctcctctcttcccagtctcccccctcctctcctttgtccccctcatccactcctccagttctcttcagaaaagaataggcttcccagccaaccatggcatatcaagttacagtaagactaggcacctcctctcctgttgaggctggatgaggcagcccagtaggaggaaaggttcctaaagcaggaataaaagtcagagatagcccttgctcctgtgttaggaatcttacaagaagaccaagctacac**aactgtaacatacgtgcagagggcctagatcagacccatgcaggcttcctggtcagtctctgtgagctcctgtgagtccaggttagttgagtctgtgggttttcttgcaatgtcctcaaactttatggttcctacattcctttaccctcttctacaggatttcctgagctctgcctaatgtttggctgtgggtgtctgtatctgttttcatcaattgctgggggaacagtctctggtaagaactgggcaaggcaccaatctatgagtatagcaggatattaggaataattttctattctaggtctctgagctatccatcttctgcatcctggccttccaggcagtgtcaggggtggtcttatggcattggtctcaagctggaccagccattgattggccattcccacaatgtctgctctagctttacccaagcacaacttgtaggtaggaccaatttgtatagttagtgttatggctagatgatatgatagttgcatttttactcttt**

**Butcher’s Bill for *Lamp2a*:**

**4 binding sites in putative promoter**

**19 intronic binding sites (inc. FoxO consensus D.B.E./ I.R.S.** ttgtttac)

**1 exonic binding site**

**Prob. of Chance Occurrence of Four FoxO3a DNA-binding Sequences within *Lamp2a* Putative Promoter = {prob. of chance occurrence of specific heptanucleotide sequence 5'-[AG]TAAA[TC]A-3' (0.5x0.25x0.25x0.25x0.25x0.5x0.25) } ^number of observed occurrences (4)^ x [opportunities for specific heptanucleotide sequence in 10 kbps. (9,994)] = 3.55 x 10^-11^**

***M.musculus Hsc-73* Gene [inc. -10 kbp. (putative promoter)] [mRNA-coding sequence (inc. actual protein coding sequence) in bold&red]**

**(http://www.ncbi.nlm.nih.gov/nuccore/NC_000075.6?from=40801273&to=40805199&report=genbank)**

tctttcaggagcctttctgtctgtatcttaagacaggataaggtcttcctaaaataaactgagcatggtgtagcctacagctttaatccaggcactagggaggcagaggcagcggatctctgtgagctgagatcagctctgtctacatagagttttccaggtcccacaaagctgcacagtgagaccctgcttcaaagtaaaaataaacgaaacaacagagatctgcctgaaatagttgccattcaagtcacacgagaaagcatacgttaatgtttaatgtggctaaatatttgttttccttaatattattttttttgtctaatataatgaaagtagatgatgttaggaggtcttatacgtttaagtaaagtaattcatactttgctacaattagaaaggcaaactatagctgggcgtggtggcgcacgcctttaatcccagcacttggaaaggaggaagaggcaggcagatttctgagttccaggccagcctggtctacaaagtgagttccaggacagccagggatatacagagaaaccctgtctccaaaagccaaaaaaagaaagaaagaaagaaagaaagaaagaaagaaagaaagaaagaaagaaagaaagaaagaaagaaagaaagaaagaaagaaagaaagaaagaaagaaagaaagaaagaaagaaaggcaaactactaatacgggtttgaatcattctagcttcaattttcctgaaagaatgcagtaaacacattcaagtcttgggcaatcaggtcacatgctgcagtttttagccatgtcattggttcctagagggactctttttgatggcctgtccatgtcggtccaccattctttgatctatcttctttcttctctccccctccaccccccccccatcccgccacacccctctcctttcccccctgaggcaggatctcactcaatcctaggctggcctcaggctcaaggcaatttttttgcctctccctcccaagtgttgggattgcacacaggagtcacaatctccagctgagtaatctttaaaacggaatacttttgttttcctttgttgtgttttttttgggggtggggtggggtggggttgggggagggttcttttattttgaaaccgggtttcaatgtagccttagatagcctcaaacttactttgtagaccaggctggcctacacaaaggtaaacctgtgtctttcctccccagtgagtgtcagtgtgttaagggcatgtgccaccacacccagcactagaagcacattttttttttcctagacaacacggtttctctgtgtagccctggctgtcctggaactcactctgtagaccaggctggcctccaactcagaaattcacctgcttctgcctcccgagtgttgggatcaaaggcgtgtgccaccactgcctggctagaagcacatattttaaactaggcaccaaggcacccatctttaattccagtactagggaggtagaggagagtgggtggctaggaggcctggaccatcttggactacatatagttccaggccagctagatagctagatagggagaccttgctttaaaaaaaaaaaaataatatagctgggtgtggtggcgcatgcctttaatcctagcacttgggaggcagaggcaggcggatttctgagttcaaggccagcctggtctacagagtgagttccaggacagccagggatacacagagaaaccctgtctcataaaaaaataaaataaaaaataaaaaatgtatgcttagaaacttccacactgggctggtgagatggctcagaggttaagagtgccgactgctcttccaaaggtcctgagttcaaatcccagcaaccacatggtggctcacaaccatccgtaacaaaaactctgatgtcctcttctggagtgtctgaagacaactacagtgtacttacatataattaataaataaatattttaaaaaaaaaagaaagaaagaaacttccacactgagctgggtggaagatttctgagttcgaggccagcctggtctacaaagtgagttccaggacagccagggctatacagagaaaccctgtctcaaaaaaaaaaaaaaaaaaaaaaaaagaaaaagaaaaagaaaaagaaaaagaaaaagaaaaagaaaagaaagaaactcccacactatttatgacttttttttttttacatttacttatttagtgatatatgtggaccatatgcaccatgacattcctatacaggtcaaggaacaaggtgcctgtgttgggtctcacctcctaacatgtgacttctagtgacagaagccaggcttggtagcaagtgcctttacctactgagccatctctttggttctttttttaattttgttttgttttagtttagtgctagaaattgaaccaaggcctccaaatgctaggcaaatgccgacgatgaccttatccatatcctatcctgaaggtgactgacttgcctctgctgtttgtggaggggtgtgtgtgcatgtggaggggcctgtgcacgtgtgctcatggaggcccaaggttgataccagcttgctctggtagcctctgtttccacctgccaagctctggaattacaaagcggctcatttttcctactaccggtgatacccagcttttggtaccacataattctaacacaaaccatctaactaaattcttagcttaagttagatagagtgtaagactctggggagccttagcttaccggggatcccttgagtgaaccatgtagagctgggaacgatgcaaaaagcaagataaatgtatgtatgtatttatttatttatttatttatttttggttttttgacacagggtttctctgtatagccctggctgtcccgcaggctggcctggaactcagaaatccgcctgcctctgcctctgcctgggattaaagggctcgcaagagaaatttattgttccagtgcattggggtcatcccagactggagggagaggcggcaacccccagcagggggttagaacagagcaccagcgactaggcacaatatgattggcagaacagtgcactctttaaactgattggtctttagggaattaggtgacaaggacttcccttgtctgatggtgggcaatggtcagtcctggggaatgtgtccccacccgcaggtccattcctgccctgtggtctgagaaatgctaattagcctctcccttccggagggggggggggagagtgtttctcgtgaccttcccaaagttcctgagctgaccttttcaagagctgcacagtaactccaacactctaaaagctgaggcagaaggatcacaagtatgaagcccatcttgcctacaaaatgagactgtctcaaaagtaaatacatagcaggctggaggggtggctcagctcagcatctgttgctctgtcagaggacacaggttcccagttctcacgcagtggttcacaactgtcccaattccagttccggggatctgaactcttctgactcccacagcaccaggcatacacacgatatacataaatacctgaatgtaaaacacccatacacacacaaaaatacgtaaattctaaaacccttacaaagggaaaagacaaaacaaaacaaaaggccgtctaccttaggctggtttacttactatttgcatagtagctaataaattatagtgatctgagtagttcctttggtattttctttaattggtggtgccagcgcactactctcgtgaaacaattacctatgatgacctagcagaacaattttacagattgatacctgtacagccttcttgtgctgcacctggtggttcaggcttgtgatcccagagactcaccggatgacacaggatgagacaggatgacacaggactgaaagttgacgtctcccaggctgcagagagctcagggcagcctcggcagtttggtaagaccctgtgtcagaattgtaaagcatctgtccatataaggaactgctatcagggagtacatatgcctatgaatcctgtttgtgttctagacccagtataagtttgggcccccctggcctttccttattagacaagcattaaatagtaagggcatgacaaattcagagtttccatgaactaaatgttccataaacccaatgtcctaaatgttctgatacttccagacttaagaaccccaagggacataaagaaaaccaaacgacagaaaaagttaaaaaagaaaaagtcctgcacattttatttaacctctacagctctgaaggttttgctctataggaaaagaagcagaaattttgagtgctacacacacttgtaatcacagaactcaagagaccaaggcaggagaattgctatgagtttgagaccatcatgggctatgtactaagtaccaagacaacatggtaagatgccgaacagaaagggggggggctgaaattatctctctgttaccacacaagaaaatgcctggaatttaaattcagatgtaactgcagagtccaaactcttgccactccctgaagctgctcaccaaagcaagattttgtgctccatactgataagacattccagggctgactaacatctcatggatttcaggattctgagacactatgaaagaaagccacaccggtccaatcttcacttaaatgtactttatttcccttttctatgaatgctgagattttaggtagcataaaacaatgtttttggtttttttttgtttaaaaaaaaaaaaaaaaccagaacaaaacaaaaactctagccaacctgagtttaaacgcctgattccaggaaagcacgcttctcacaaaggactgttttccctccttcccagagagagaagaggaaaacacaagaacaaatcctggtgcagagcccattcctgcttcaaggaagtgagctgccagggatgccacagaaagtccatggtcttcagccaggcacagacacagacacttttacgtgttggctgctccactggccttagcaggcttcaggagaaaagataaggcttacggaaggaagttccctctggaggaagcagagctgaaaactgatcccaataaacacatttgagattagagaaaacaaaaacagacagacagacagaaaccctggtctgatggtttaccagtttaaccatttaacctttcctcatctacaaaatgaaaaccatgccaattcgtacttcttaagatttgaggtaaatctgttgttgtttgagttgtagctcaactcaaatcctcttgcctctgcattttgagtgcagacatgtgctcctatacctgaccttgtcatgagaattaaaccagttgtgtatgccacatacttaaaatagagcttaatagtgttgtatgccacatgacaggcacacagcagtgtaaatcattattcaatttcgagtcagaaccccatgctccaaatttctccattataagtcccaagcacccaagctgtgatttatttattattattattttttaaaggtttactattatacataagtacactgtagctgacttcagacacatcagatctcattatggagggctgtgagccaccatgtggttgctgggatttgaactcaggaccttcagaggaacagtcagtgctcttacccgctgagccatctcgccaaccccatattattattatttactttttttcttttctttttttttttttttttgttttatcaagacagggtttctctgtgtagccctggctgtcctggaactcactgtgtagaccaggctggcctcaaactcagaaatctgcctgcctctgcctcccgagtgctgggattaaaggtgtgtgccaccatgcccggctattttggttttttgagacagggtttctctgtgtagccatgactgtcttggaactcaatcttgtaaaccaggctggtctggaacttaggaattggcctgcctctgactcccaagtgctgagatttataggtgtgtacaagcaccctgctctttgcttctgcttctgctcctgcttctgcttctgctcctgcttctgcttctgcttctgctcctgctcctgctcctgctcctgcttttcttctttcttcttcttcttcttcttcttcttcttcttcttcttcttcttcttcttcttcttcttcttcttcttcttcttcttcttcttcttcttcttcttcttcttcttcttcttcttcttcttttttaaacagtcttaatgggtggcctaagatagcctagaactagcttttttttttttctatctccacctcctgagatctgggcttataggtgcatatcacatgcttggtttatttcagattgtgaataagtaaattttctgtttgaaaccctgagatgctagaagcaaaggaacaactttcactatatatataatgggattaaaggcgtgtgccaccaccgaccggctcattatattttctgcttttatttggtcaataattttattttttataatggaaggagcactcatgccatggagcacgtaagaaggtcatagtacaaccctgaaggaccagtttgcctctctctctctctctctctctctctctctctccctctctctctctctcttccccctttctttcaatagcttccaagaatcaaactcatgtcttcaggtttgcatggcaagcactttacccatcgagctaggtggtcttttgtgcttttgatatattagatagtaaaaacagctcaaattcaagagcacattttgagtgacaaaggcagagattaattcttctgtctaaaaactatttctgtgcatgtgtgtatgtgtggtgtgtatgtgcaggggtgtggacctgtgaggtgtcggggatcatactagaccagagttctaccttcttggcctgggactggaatttagcagtgattaatctggccagaagccctagaaaattatacctcgcctccacctataactactctcaggtaatctgacacccttacacgtagatacagtaataaaaataaatcttctttaaaagatccccattccactttcaggacttctgactttactttgtgacccacaggattaatgaaggctgcctttgtgggcccaagtgtgcgcttagcagctagagcaaagcttactcacaccagtggttccccactgaagacagacttcctctccactagggtgaggcagagcccctgagcccctccccatttgtgactatttagacagtccgtcttgagaggatcccatgcagtgatggcagccactgactccggagtgccactgccatgtcatgcccaggatgtcataatcctctgcctctttcttctttcaaccccctcttccctcatgcttcctgagtctgggaggtgatacgacatccctgtaaggttgagcacctatcagtcgattgaactcagcattaacctaaccggcattctctgcagagggaagctgagactgcactaatctacaaccccctgtcttgcagggcctcaaatgtcccctcctccttttctgtctcttgataagtgagattataggttggctcctgagatgcctcggctggaatggtcatctgataaagtgaagcctggggccactaagactcactagaagccgggcatggtgacccacgcctttaattccagcacttgggaggcagaggcaggccaattcctaaattccagaccagcctggtctacaaagtgagttccaggacagccagggctacacagagaaaccctgtctcaaaaaacaaaaacaaaaccaaacaaacaaaaagacttcactagagttagtcaagtcttttccctgtagatggggtttctggtgagattcctggaagcttgcaggctcatggctgggaaggtagaagggtactaaggagttgttaatttgctggaattcttatttttccactggcaagaggcttcaactggattgccaggcaaggggcctgctctctccttctcctaccctcagcatttcctgtctccccatgctgcctcagtttgacttcttttgagacggggtttcactgagttgctcaggcaagcctttgtttgggcagggcagggcaggcaggccttgttttagactctggagtcgcaagggtcacaggcctgaaccacccacctcccatcccagaatgtaagcttgtgaaacagactccggctctaaatttttaaaaatttacttacagactgacagtctcatatatcctaacttctgtctagcaaaacttttgatccttctcctcagtgctaactaaaagctcgaaccaccacaccttagcttaaagaaaaaagaaaaaagtggagagggtgattccaagaaaaaacagttgtgtctattcctataagtaatgtctctgtgtctcactttccctcaggtttctaatatggggtactacataccttagaaaagtgttttgatatttatagagagcactttacatgttatgtacactttaggttagcaaaaagcattgtattaaaacgatcttcttgaactgcaacagagttacatccttgtacatcctcccatgaactccaagccaacattcctcccgagatgcaaataccaaagaaaaaatttatttatgactgtcttagtgattactagcttttgacaaggacaaagagtccccggtcttccgctagccttcctcacgtggtctggagaccttccaatttaaacgccacaacagtattctttaaaaaataaaaagacaacctatctaactaataaagagctgcactccctgcctatttgaaaggacgcgtggaaagtgctagagagccgggtgtgacgaggcccggaacgcccagcctccaggcactgcggtttcagagtctaagcactcgctccggccattggctcagtcacgttttcaaggactcagcgcggccgggcgtttaatttttggtaaaagtcaagtcaggagagacattacctcatgttactgaaaataaaaaggtcgcgatcactctatcaaggacacggggctgccggaaaccaggaatgccagtgcctagctcacccacaaagggacaaaaaacatttatctttcgtacatttattccattaactgatgagtctggtggagcagagaggggacagaggtcacgctgtcccctgtgggacagcggaggtggggaggggcgttcggctcaggggtaagcagagaggactgggccatttctttgatagaggatcacaaacgatcctggggacatgtggcgagccaggcagatcgagcctccgcactggggggggggggaatcgaggtgtcccattttgtggggggtgaggggtggggaaagccagcgagatagcgcggcggcttagcctcccacaagctggcagcacgaaacaagaagcctagcagtgctccttagtctcaggctggggtggaccaagcccgaggccgggaaaccgccgggctcagggagaaggaccgagggtcgtgtgggggaggggcgcaacaaccttcgcagccattttgtcctcgccccacttccgtcttctcttcccggactccccctccctcaggctcttctctcaggcagcccggggtcccaagaggtagggatccggccgaacgctgctctcattggtccgtcgcggaggtacccgccttcctcccagggcatccttccgccccctcccgcctgcgctgccgggtcgcgcacgcgcactcgccggcgaccctctcccctcccccagcagctctgagtgacagaggactcccgcgcgcgggctcccggcgccccgccttctccgctccccccgggccgttcccagagcgggggcgagcgtgaaagttccagaacgccgcggtcgtgcgtcatcgcgaggcggggcggggagaaggcgggacctaggcgagcgttctggctcttcattagtgcagtcggcaagggctggtcgtccattggctaagaagctatgatcggcagcgggatggggcggcacccttctggaaggttctaagacagggtataagagagagggaagcgggcggaaacc**ggtctcattgaacgcggaggcagctgcctggcatttgtgtggtctcgtcgtcagcgcagctgggcctacacacaag**gtgaggcatgggactcgggggtttgcgggggtccggggtggtgcaggagtcccggggagtctcttaggcggcggctgcagctcaggccgtcaccaccagggacagagactgcagcagatccggaggatgggctgagtcagggcggcgccgccatggccttgtggctccgcgtggcgggggaggggagccacaggcgccctggtctttttatcctgtctgtgacttcaaatataaggcttctgcccggcgcccacagggcagggccccccgaggtgggaaagggggatcctggctcccgcggtgcgcggtccgggtggggggggttcggggggcgctcggaggtggccacgtgctccaccattgtcgcagacctttaggacagatggttccttgagccttgggcgggctgtgccaggcttggcacagaccacctatactgcagcactgctgccacgagcctgccgcgtggcttcaggtgataggtctgggtcaccaagtccagacccactgcggactggataaaagccggaaagcgagtgcttagcccccctcctttttttttctaatag**caaccatgtctaagggacctgcagttggcattgatctcggcaccacctactcctgtgtgggtgtcttccagcatggaaaggtggaaattattgccaatgaccagggtaaccgcaccacgccaagctatgttgctttcacggacacagagagattaattggggatgcggccaagaatcaggttgcaatgaaccccaccaacacagtttttg**gtaagtcgccagcatctttgtgccactagcatgagtaaaagggctggggggcagggcatttggaatgtagaaacagatctgccttaataactgcagcctgagattgcaactgtttaaatatgacaatataaggccacaagattttaaatatgggccttggcttcctgcactagagattacgtccatctagatgggtgctgcaggtttttgttttctgtttttttggttttttttttttttgcagtgataggcactaaggagtgtgatgaggtatgtttaatattttacag**atgccaaacgtctgatcgggcgtaggtttgatgatgctgttgttcagtctgatatgaagcactggcccttcatggtggtgaatgatgcaggcaggcccaaggtccaagtggaatacaaaggggagacaaaaagtttctacccagaggaagtgtcctccatggttctgacaaagatgaaggaaattgcagaagcgtacctcggaaag**gtgggttttttttttaacaggtgccatttgagatgggtgttacctttatgtttaattcacacattctgatcgggaatttgccaaccaaacaattgtgcaattcattgttttttag**accgttaccaacgctgtggtcacagtgcccgcttacttcaatgactctcagcgacaggcaacaaaagatgctggaactattgctggcctcaatgtacttcgaatcatcaatgaaccaactgctgctgctattgcttacggcttagataagaag**gtaggcatttcaaatcaagtacaactttgttagggttaacttgccagaaacaggcctgagctaagaatgtcttatttgacag**gtcggagctgaaaggaatgtgctcatttttgacttgggaggtggcacttttgatgtgtcaatcctcactattgaggatggaatttttgaggtcaaatcaacagctggagacacccacttaggtggagaagattttgacaaccgaatggtcaatcatttcattgctgagttcaagcgaaagcacaagaaagacatcagtgagaacaagagagctgtccgccgtctccgcacggcctgcgagcgggccaagcgcaccctctcctccagcacccaggccagtattgagattgattctctctatgagggaattgacttctatacctccattacccgggctcgatttgaggagttgaatgctgacctgttccgtggcacactggaccctgtagagaaggcccttcgagatgccaagctggacaagtcacagatccatgatattgtcttggtgggtggttctaccagaatccccaagattcagaaacttctgcaagacttcttcaatggaaaagagctgaacaagagcattaaccccgatgaagctgttgcctatggtgcag**gtaaatacatacagcctttatagtctttataatctcaacaagtcctgtaagattcgctgtgatgatggattccaaaaccattcgtagtttccaccagaagtactgtgttggctagttccttccttggatgtctgagcgaatgaaacatacatatgttgagtagactgcagttacagtgcgttgtttctaacattttttttctttttatag**ctgtccaggcagccattctatctggagacaagtctgagaacgttcaggatttgctgctcttggatgtcactcctctttcccttggtattgaaactgctggcggagtcatgactgtcctcatcaagcgcaataccaccatccccaccaagcagacacagactttcaccacctactctgacaaccagcctggtgtactcattcag**gtatgttgtccctagaattttgtagcttgtatagaaagcccagggaagttcctcgctgtgatgatggattccaaaaccattcgtagtttccaccagaaatgctgtgttggctagttccttccttggatgtctgagcgaacttatcttccccaaatttgagcttaagccacacgagcagtagtttctaaaattggatgtcttaatttttattgtctcttctag**gtgtatgaaggtgaaagggccatgaccaaggacaacaacctgcttggaaagttcgagctcacaggcatccctccagcaccccgtggggtccctcagattgaggttacttttgacatcgatgccaatggcatcctcaatgtttctgctgtagataagagcacaggaaaggagaacaagatcaccatcaccaatgacaagg**gtaagtatggacctgctgctttctttctgaatagaaagaacaaggtttgctcttaccattaaagagacatgctcatgattttgtaactttcatcatcag**gccgcttgagtaaggaagatattgagcgcatggtccaagaagctgagaagtacaaggctgaggatgagaagcagagagataaggtttcctccaagaactcactggagtcctatgccttcaacatgaaagcaactgtggaagatgagaaacttcaaggcaagatcaatgatgaggacaaacagaagattcttgacaagtgcaatgaaatcatcagctggctggataagaaccag**gtatgtgttttgccaataagtagatgttcattcagaaaagggctgagaggggtcacaatgatgatgaatggtccaaacattcgcggtttccaccagaacgcaaggcagtgttggcagttaccttccttggatgtctgagtgactcaatagctaagggttctttaggcttagttgtcagtctcctccccccaaagtaattggagatcttttaataaatttgatttcttttttctcag**actgcagagaaggaagaatttgagcatcagcagaaagaactggagaaagtctgcaaccctatcattaccaagctgtaccagagtgcaggtggcatgcctgggggaatgcctggtggcttcccaggtggaggagctcccccatctggtggtgcttcttcaggccccaccattgaagaggtggattaagtcagtccaagaaggtgtagctttgttccacagggacccaaaacaagtaacatggaataataaaactatttaaattggcacc**

**Butcher’s Bill for *Hsc-73*:**

**6 binding sites in putative promoter**

**1 intronic binding site**

**Prob. of Chance Occurrence of Six FoxO3a DNA-binding Sequences within *Hsc-73* Putative Promoter = {prob. of chance occurrence of specific heptanucleotide sequence 5'-[AG]TAAA[TC]A-3' (0.5x0.25x0.25x0.25x0.25x0.5x0.25)} ^number of observed occurrences (6)^ x [opportunities for specific heptanucleotide sequence in 10 kbps. (9,994)] = 2.12 x 10^-18^**

**Figure S26. Middle-aged GHR-KO Mouse has More Brown Adipose Tissue than its Littermate Control.**


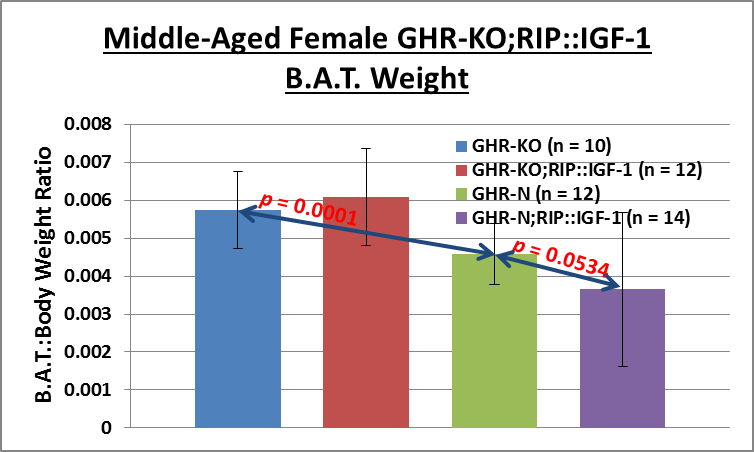


**Table S1.**

| **Supplemental Table I. Home-Cage Assessment Rubric** | | |
| --- | --- | --- |
|  |  |  |
| **Condition** | **Rating** | **Description** |
| Initial Posture | 1 | Sitting or standing normally, rearing or asleep |
|  | 2 | Crouching over or lying low |
|  | 3 | Slight sway while in standing position |
|  | 4 | Excessive sway or head bobbing in standing position |
|  | 5 | Flattened, limbs may be spread out |
|  | 6 | Lying on side, limbs in air |
|  |  |  |
| Salivation | 1 | None |
|  | 2 | Slight |
|  | 3 | Severe |
|  |  |  |
| Lacrimation | 1 | None |
|  | 2 | Slight |
|  | 3 | Severe |
|  |  |  |
| Fur | 1 | Normal, silky and smooth |
|  | 2 | Pilo-erection |
|  | 3 | Over groomed |
|  |  |  |
| Vocalization | 0 | No, spontaneous vocals |
|  | 1 | Yes, spontaneous vocals |

**Tables S2a & Table S2b, related to Table 1 & Table 2, respectively.**

| **Supplemental Table IIa. Legend for Indirect Calorimetry-based Metabolic Dependent Variables** |
| --- |
|  |
| **VO_2_** (Volume of oxygen consumed per hour, normalized to body weight.) |
| **VCO_2_** (Volume of carbon dioxide produced per hour, normalized to body weight.) |
| **Respiratory Quotient (Respiratory Exchange Ratio)** (VCO_2_/VO_2_) |
| **Heat Production** [((4.33 x VO_2_) + (0.67 x VCO_2_)) + (Wt.(kg.)) x (60 Min./Hr.)] |
| **Energy Expenditure** [VO_2_ x (364 + 113 x R.Q.)/22.4] |
|  |
|  |
| **Supplemental Table IIb. Legend for Voluntary Locomotion Dependent Variables** |
|  |
| **Total Distance** [The total distance that the subject has traveled. For the purpose of this variable, the location of the subject is defined as the centroid (center of mass) of the subject.] |
| **Horizontal Activity Count** [A count of sensor changes (beam breaks).] |
| **Ambulatory Activity Count** (Number of beam breaks while animal is ambulating. Does not include stereotypy behavior.) |
| **Rest Time** (The length of time that the subject spent at rest. A resting period is defined as a period of inactivity greater than or equal to 1 second.) |
| **Rest Episode Count** (The total number of resting periods. A resting period is defined as a period of inactivity greater than or equal to 1 second.) |
| **Movement Time** (The length of time that the subject spent in activity. Activity is defined as a period in which ambulation or stereotypy occurred.) |
| **Movement Episode Count** (The total number of locomotor episodes. Episodes are separated by rest periods of at least 1 second.) |
| **Stereotypy Time** [The total amount of time that stereotypic behavior is exhibited. A break in stereotypy of 1 second or more is required to separate one stereotypic episode from the next. If the animal breaks the same beam (or set of beams) repeatedly then the monitor considers that the animal is exhibiting stereotypy. This typically happens during grooming, head bobbing, *etc.*] |
| **Stereotypy Episode Count** [This corresponds to the number of times that stereotypic behavior was observed in the animal. A break in stereotypy of 1 second or more is required to separate one stereotypic episode from the next. If the animal breaks the same beam (or set of beams) repeatedly then the monitor considers that the animal is exhibiting stereotypy. This typically happens during grooming, head bobbing, *etc.*] |
| **Stereotypy Activity Count** [Number of beam breaks that occur during a period of stereotypic activity. If the animal breaks the same beam (or set of beams) repeatedly then the monitor considers that the animal is exhibiting stereotypy. This typically happens during grooming, head bobbing, *etc.*] |
| **Vertical Episode Count** (Each time the animal rears up, this is incremented by 1. The animal must go below the level of the vertical sensor for at least 1 second before the next rearing can be registered.) |
| **Vertical Activity Count** (Cumulative vertical beam breaks.) |
| **Vertical Activity Time** (When the animal rears up, this timer starts incrementing.) |
| **Locomotor Clockwise Revolutions** (Counts the number of clockwise revolutions that the subject travels in an open field.) |
| **Locomotor Counter-Clockwise Revolutions** (Counts the number of counter-clockwise revolutions that the subject travels in an open field.) |

**Table S3a, related to Table 1.**

**Table S3b, related to Table 1.**

**Table S3c, related to Table 1.**

**Table S3d, related to Table 1.**

**Table S3e, related to Table 1.**

**Table S4a, related to Table 2.**

**Table S4b, related to Table 2.**

**Table S4c, related to Table 2.**

**Table S4d, related to Table 2.**

**Table S4e, related to Table 2.**

**SUPPLEMENTAL EXPERIMENTAL PROCEDURES**

*Weekly Body Weight Determinations and Food Consumption Measurements*

Mice were weighed (in the late afternoon) weekly for the first six months, then once every four weeks, on a Scout Pro Balance (Ohaus Co., Pine Brook, NJ) that was calibrated to weight standards on a monthly basis.

200 grams of the above-described food was placed in each cage-hopper on a weekly basis. After six days of food consumption, the remaining food was weighed on a Scout Pro Balance (Ohaus Co., Pine Brook, NJ) that was calibrated to weight standards on a monthly basis; the food consumption values analyzed and plotted were calculated as follows: {(200 g.) – [food remaining after six days (in g.)]}/six (days)/number of subjects in cage.

Daily food consumption was also determined for nine-month-old females on a genotype/age/cage/cage pop. basis wherein the measurements were collected daily for seven consecutive days. The intergroup comparisons of food consumption yielded comparable results regardless of methodology.

Metabolizable energy (M.E.) is empirically defined as the amount of gross energy available to the animal once the energy from feces, urine, and combustible gasses are removed. Basically, M.E. is the energy left for the animal to use once all digestion is complete (Nestlē-Purina, St. Louis, MO). Metabolizable energy consumption was calculated as 3.02 kilocalories/gram of Lab Diet Formula 5001, based on food-manufacturer’s proscription against gross energy or physiological fuel value calculations (Nestlē-Purina, St. Louis, MO).

*n.b.*: Mice were housed in genotype-, gender-, and age-specific cages.

*Body Length Measurement*

Live mice were gently manually immobilized, and measured snout-to-anus to the nearest quarter-centimeter.

*Age-Grade Classification*

Subjects were young-adults at testing for all experiments; save for the confirmatory indirect calorimetry trials, the spontaneous locomotor activity experiments, and the behavior (anxiety & memory) experiments, during which subjects had to be middle-aged in order to address gerontological queries.

Age-staging was based on a combination of 1) quantitative extrapolation from prior stock-specific survivorship data [Bonkowski *et al.*, (2006); Arum *et al.*, (2009)], 2) presence/ appearance of aging-associated wizening (as represented quantitatively by declining body weight), and 3) spontaneous, testing-independent, (and thus, presumably) aging-resultant mortality. Thusly, young-adulthood is marked by at least 90% of reproductively competent negative control subjects being alive; middle-age is the period between when approximately 90% of the control subjects are still alive and median survivorship; old-age is the period between median survivorship and when approx. 10% of the subjects are alive; and oldest-old age is designated as the period when ≤ 10% of the controls remain.

*Blood Glucose Regulatory Assessments*

All animals underwent home-cage assessments of gross health (Supplemental Table I) and any animal exhibiting questionable health by these criteria, or which was aberrantly hypoglycemic at the inception of a test, was excluded from the testing and/or data analysis. Also, all animals were given at-least two weeks of recuperation in-between tests.

Glucose Tolerance Tests [*ad libitum (A.L.)*-fed or Fasted]:

For A.L.-fed tests, animals had access to food for at least 16 hours before the test. For fasted tests, animals were fasted for 16 hours. 30 minutes prior to the beginning of the test, each animal was weighed, had a small nick placed at the tip of its tail with a razor, and re-housed without access to food. After 30 minutes to recover from the handling stress of the weighing and tail-nicking, each animal was measured for blood glucose concentration, of the blood obtained by applying gentle pressure to the tail-tip, with a blood glucose monitoring system (glucometer and test strips) (OneTouch Ultra 2, Lifescan, Inc., Milpitas, CA); without releasing the animal, it was also injected intra-peritoneally with 2 g. D-(+)-glucose (Sigma-Aldrich Co., St. Louis, MO) per kg. of body weight. (The powdered glucose was dissolved in 0.9% sodium chloride.) Subsequent blood glucose measurements were at 10, 20, 30, 40, 50, 60, 75, 90, and 120 minutes after the injection. Animals were given A.L. access to food immediately after completion of testing.

Insulin Tolerance Tests (0.75, 0.6, 0.3, or 0.0375 U.S.P.U.):

Animals had access to food for at least 16 hours before the test. 30 minutes prior to the beginning of the test, each animal was weighed, had a small nick placed at the tip of its tail with a razor, and re-housed without access to food. After 30 minutes to recover from the handling stress of the weighing and tail-nicking, each animal was measured for blood glucose concentration, of the blood obtained by applying gentle pressure to the tail-tip, with a blood glucose monitoring system (glucometer and test strips) (OneTouch Ultra 2, Lifescan, Inc., Milpitas, CA); without releasing the animal, it was also injected intra-peritoneally with either 0.75, 0.6, 0.3, or 0.0375 United States Pharmacopeia Units (U.S.P.U.) of porcine insulin (Sigma-Aldrich Co., St. Louis, MO) per kg. of body weight. (The lyophilized insulin was dissolved in 0.9% sodium chloride.) Subsequent blood glucose measurements were at 10, 20, 30, 40, 50, 60, 75, 90, and 120 minutes after the injection. Animals were given A.L. access to food immediately after completion of testing.

Pyruvate Conversion Test:

Animals were fasted for 16 hours. 30 minutes prior to the beginning of the test, each animal was weighed, had a small nick placed at the tip of its tail with a razor, and re-housed without access to food. After 30 minutes to recover from the handling stress of the weighing and tail-nicking, each animal was measured for blood glucose concentration, of the blood obtained by applying gentle pressure to the tail-tip, with a blood glucose monitoring system (glucometer and test strips) (OneTouch Ultra 2, Lifescan, Inc., Milpitas, CA); without releasing the animal, it was also injected intra-peritoneally with 2 g. of sodium pyruvic acid (Sigma-Aldrich Co., St. Louis, MO) per kg. of body weight. (The lyophilized sodium pyruvate was dissolved in 0.9% sodium chloride.) Subsequent blood glucose measurements were at 15, 30, 45, 60, and 120 minutes after the injection. Animals were given A.L. access to food immediately after completion of testing.

Non-stimulated Blood Glucose Comparisons [*ad libitum* (A.L.)-fed or Fasted]:

Un-stimulated blood glucose values were obtained from young-adult mice at the beginnings of A.L.-fed and fasted glucose tolerance tests, drawn from a small nick at the tip of the tail and measured with a blood glucose monitoring system (glucometer and test strips) (OneTouch Ultra 2, Lifescan, Inc., Milpitas, CA). A.L.-fed blood glucose values were collected after an overnight (~ 16 hrs.) period of A.L. feeding for all subjects; fasted blood glucose values were gathered after equivalent overnight fasting (~ 16 hrs.) for all subjects.

A.L.-fed blood glucose values recorded immediately preceding a sacrifice & tissue harvest from middle-aged mice were consistent with the results obtained as above, *save for the slight differences in A.L.-fed blood glucose comparisons recorded from young-adult females*.

*Ho*meostatic *M*odel *A*ssessment of *I*nsulin *R*esistance (HOMA-IR) Calculation:

HOMA-IR (Score) = {Glucose (mg/dL) x Insulin [arbitrary units (A.U.)]} / 22.4

HOMA-β (Score) = [20 x Insulin (mg/dL)] / [Glucose (mg/dL) - 3.5]

*A.L.-fed and Fasted Indirect Calorimetry*

Indirect calorimetry was conducted as previously described by Westbrook *et al.*, (2009) (Accuscan Instruments, Inc., Columbus, OH)*,* save that 1) acclimation day testing, A.L.-fed day testing, and fasted day testing were all conducted in one longitudinal stretch; and that 2) data were normalized per unit of lean body weight [Butler&Kozak, (2010)] as determined by fat depot sub-dissection [Muzumdar *et al.*, (2008); Berryman *et al.*, (2010)]. The 17:00 hour was excluded from the statistical analyses, as it was used for maintenance activities (*e.g.* removal of food, weighing of remaining food, and weighing of mice) during longitudinal testing paradigm. Parameters assessed are annotated in Supplemental Table IIa.

*A.L.-fed and Fasted Spontaneous Locomotor Activity*

Spontaneous locomotion was assessed in the same equipment as, and simultaneous with, indirect calorimetry analysis above (Accuscan Instruments, Inc., Columbus, OH). The values measured at the 17:00 hour were excluded from the statistical analyses, as it was used for maintenance activities (*e.g.* removal of food, weighing of remaining food, and weighing of mice) during longitudinal testing paradigm. Parameters assessed are annotated in Supplemental Table IIb.

*Core Body Temperature (T_co_) Assessment*

Core body temperature was assessed, as another proxy of life expectancy, using implantable temperature transponders and a radio-frequency-based detection wand (Bio Medic Data Systems, Inc., Seaford, DE).

*Fat Depot Sub-dissection*

Mice were weighed and sacrificed with Isoflurane (Halocarbon Laboratories, River Edge, NJ). Visceral (perigonadal, perinephric, retro-peritoneal, and mesenteric) and subcutaneous (white and brown) adipose tissue depots were manually dissected and weighed [Muzumdar *et al.*, (2008); Berryman *et al.*, (2010)]. Ratios of depot weight to body weight, as well as lean body weight (Total Body Weight – Total Sub-dissected Adipose Tissues Weight), were calculated.

*Complete Blood Cell (CBC) Count Analysis of Hematopoietic Cell Parameters*

Blood cell counting was accomplished with a VetScan HM2 Hematology System (Abaxis, Union City, CA) and ≥ 25 μL of whole blood [collected in EDTA-coated Microvette 100μL capillary tubes (Sarstedt AG & Co., Nümbrecht, Germany)] drawn from *ad libitum*-fed subjects. The following 18 parameters were assessed: concentration of leukocytes/ white blood cells (W.B.C.), concentration of lymphocytes (LYM), concentration of monocytes (MON), concentration of granulocytes (GRA), proportion of leukocytes that are lymphocytes (LYM%), proportion of leukocytes that are monocytes (MON%), proportion of leukocytes that are granulocytes (GRA%), concentration of erythrocytes/ red blood cells (R.B.C.), concentration of hemoglobin (g/dL) (HGB), hematocrit (%) (HCT), mean (erythrocytic) cell volume (M.C.V.), mean corpuscular hemoglobin (pg.) (M.C.H.), mean corpuscular hemoglobin concentration (g./dL) (M.C.H.C.), red cell (erythrocytic) distribution width (%) (R.D.W.), concentration of thrombocytes/ platelet cells (PLT), mean platelet volume (M.P.V.), plateletocrit/ platelet hematocrit (%) (PCT), platelet distribution width (%) (P.D.W.).

*Energy Homeostasis-Regulating Biochemical Assays*

Plasma insulin and glucagon were measured with the multiplexed Mouse Endocrine Lincoplex ELISA; plasma (total) adiponectin with the Mouse Single-Plex Adiponectin Lincoplex ELISA; and plasma leptin, resistin, monocyte chemoattractant protein 1 (MCP-1), tissue plasminogen activator inhibitor 1 (PAI-1), interleukin 6 (IL-6), and tumor necrosis factor alpha (TNF-α) with the multiplexed Mouse Serum Adipokine Lincoplex ELISA (LINCO Research, St. Charles, MO).

Plasma IGF-1 was measured with the Mouse IGF-1 Immunoassay (R&D Systems, Minneapolis, MN). For IGF-1 ELISA kit:

1: Intra-assay Accuracy/ Consistency: Samples of known concentration = 3, n (replicates/sample/1 plate) = 20, mean (pg/mL) = 82-921, S.D. = 4.6-37.7, & C.V. (%) = 3.3-5.6;

2: Inter-assay Accuracy/ Consistency: Samples of known concentration = 3, n (plates/sample) = 35, mean (pg/mL) = 86-863, S.D. = 7.8-37.4, & C.V. (%) = 4.3-9;

3: Sensitivity: Minimum Detectable Dose (M.D.D.) of mouse/rat IGF-1 for 26 assays = 1.6-8.4 (mean = 3.5) pg/mL;

4: Specificity: No significant cross-reactivity or interference observed w/ the following recombinant mouse proteins: EGF, EGF-R, FGF-8b, FGF-8c, IGF-2, IGFBP-1, IGFBP-2, IGFBP-3, IGFBP-5, IGFBP-6, NGF-R, or PDGF-C (or with certain other rat or human recombinant growth factors);

5: Mouse IGF-1 Positive Control that came with kit was supposed to have a concentration in the range of 214-357 pg/mL, and was ultimately measured at 234.273 pg/mL +/- 8.126 pg/mL.

*Lipid-based Energy Homeostasis-Regulation Assays*

Plasma total cholesterol and plasma triglycerides were assessed by colorimetric assays (Pointe Scientific, Canton, MI). Plasma high density-lipoprotein (HDL)-cholesterol, plasma low density lipoprotein (LDL)-cholesterol, and plasma non-esterified fatty acids (NEFA’s) were also assessed by colorimetric assays (Wako Diagnostics, Richmond, VA). Blood β-hydroxybutyrate was assessed by Precision Xtra Blood β-Ketone Monitor and Test Strips (Abbott Diabetes Care, Alameda, CA) [Wallace *et al.*, (2001); Wallace *et al.*, (2008); Iwersen *et al.*, (2009)].

*One-, Two-, and Five-Min. Open-field Chamber Anxiety/ Exploratory Inclination Assay*

All animals underwent home-cage assessments of gross health and locomotor ability and activity (Supplemental Table I). Any animal exhibiting questionable health by these criteria was excluded from the testing.

During the light-phase of their day, mice were individually placed in the center of a lid-less, opaque, white, 44x44x40 cm. (length x width x height) polymer box with floor divided into 16 11x11 cm. squares (Supp. Figure 23). Number of squares entered within the allotted time was noted per mouse per trial; as the experiment is contingent upon the novelty of the aberrant context, each subject was only tested once. Methods are derived from standard methodologies previously used by other investigators “to analyze general activity and exploratory drive” [Selman *et al.*, (2009); Crawley, (2007)].

*Open-field Chamber Proximal Long-term Memory Assay*

All animals underwent home-cage assessments of gross health, and locomotor ability & activity (Supplemental Table I). Any animal exhibiting questionable health by these criteria was excluded from the testing.

During the light-phase of their day, mice were individually placed in the center of a lid-less, opaque, white, 44x44x40 cm. (length x width x height) polymer box with the floor divided into 16 11x11 cm. squares (Supp. Figure 23). The number of squares entered within one minute was noted per mouse per trial; 24 hours after initial evaluation (acquisition), subjects were re-tested (retention). Memory index values were calculated per mouse as follows: (Retention activity /Acquisition activity); and is reflective of the degree to which the subject remembered the context presented 24 hours prior (with enhanced memory putatively resulting in more movement due to less anxiety). Final location scores evaluate the ultimate (after the 60-second testing interval) position of a mouse on the retention day, with a more-ensconced placement being indicative of greater anxiety (and, thus, worse memory of prior context) than a more-exposed positioning. Methods are derived from standard methodologies used by other investigators [Crawley, (2007)].

*Passive Avoidance Distal Long-term Memory Assay*

All animals underwent home-cage assessments of gross health and locomotor ability and activity (Supplemental Table I). Any animal exhibiting questionable health by these criteria was excluded from the testing.

The apparatus used was an automated inhibitory avoidance chamber and its accompanying software [(Hamilton-)Kinder Scientific, Julian, CA)], consisting of a trough-shaped alley measuring 24x21x18 cm. The chamber was divided into two compartments, one lit by lights on the roof and another that is not lit, separated by an automated guillotine door. The floor of the chamber comprised an array of metal rods (0.25 cm in diameter, 0.5 cm apart). The chamber delivers an electric shock (0.5 mAmps for 0.5 second) to the floor of the darkened compartment.

During the light phase of their day, each mouse received one trial during each acquisition and retention test, and was returned to its home-cage promptly after the conclusion of the trial. The acquisition trial began when the mouse was placed in the lit compartment facing the guillotine door, and the lid was closed. After 60 seconds of acclimation time, the guillotine door was raised. The mouse was given 300 seconds to enter the darkened chamber, by natural nocturnal inclination. If it did so, the guillotine door lowered, and the shock was applied. The mouse was then manually returned to its home-cage. The time spent in the lit chamber is used for statistical analysis. The retention trial was conducted 50 days after the acquisition trial; and was identical to the acquisition trial, save the application of the shock. The persistence of memory retention trial was conducted 24 hours after the retention trial, which was identical.

*Data Presentation and Statistical Analysis*

Graphs were generated with Excel (Microsoft, Redmond, WA). All measures of central tendency are arithmetic means, and all depictions of variation (error bars) represent standard deviations (S.D.) [Glantz, (2002)].

Pre-*Hoc* Statistical Measures:

In brief, experimental design approaches were taken to maximize robustness while lessening the potential need to increase sample size; utilizing 1) an *a priori* specification of a limited number of well-defined hypotheses, 2) refinement of experimental techniques, and 3) grouping of animals so that the effect of unit variability on the treatment was minimized.

Post-*Hoc* Statistical Analysis:

Levene’s tests (to investigate scedasticity) and Kolmogorov-Smirnov tests (to determine deviations from Gaussian distribution) were conducted to guide the choice of statistical algorithms for analysis of differences amongst groups. The combined parameters of effect size and Type 1 error probability were considered when determining phenomena meriting presentation and discussion.

Most data were contrasted with unpaired, homoscedastic Student’s *t*-test, Analysis of Variance, or Analysis of Variance for Repeated Measures (ANOVA or ANOVA-RM, resp.), as appropriate; followed by the Tukey’s Honestly Significant Difference (H.S.D.) or the Dunnett’s *t*-test post-*hoc* tests for multiple pairwise comparisons, as appropriate.

Middle-aged open-field memory assessment data contrasting “Contribution of Final Location” were analyzed with Chi-Squared (χ^2^) Test for Independence.

For repeatedly measured blood glucose regulatory assessments, the *p*-value for a given pairwise comparison at a given time-point represents the result of testing all of the timepoints, up-to-and-including that timepoint, within the repeated measures analysis; this permits testing of whether both groups have experienced similar excursions in blood glucose (the null hypothesis) *relative to their initial values* ***and*** *with consideration of all intermediate values*. This mode of analysis permits the posing of more discrete, and more descriptive, inquiries than analyses of the entire area under respective curves; or that utilizing isolated, independent blood glucose values/ percentages at lone timepoints. The data that is normalized to initial blood glucose values was used for the precise, timepoint-specific *p*-values reported, yet the inferences of differences amongst groups do not depend on the use of this normalized data. For a particular pairwise comparison within a particular assay, the *p*-value reported in the text is the most-conservative (*i.e.* highest) sub-0.05 *p*-value from the series of repeated measures analyses conducted. Simple area-under-the-curve (A.U.C.) analyses were also conducted for all tests of regulation of blood glucose homeostatic dynamics.

Statistical comparisons were conducted with PSPP for Windows.

**SUPPLEMENTAL REFERENCES**

**1. Acheson K, Jequier E, Wahren J.** (1983). Influence of beta-adrenergic blockade on glucose-induced thermogenesis in man. J Clin; 72: 981-986.

**2. Acheson KJ, Ravussin E, Wahren J, Jequier E.** (1984). Thermic effect of glucose in man. J Clin Invest 1984; 74: 1572-1580.

**3. Anselmi CV, Malovini A, Roncarati R, Novelli V, Villa F, Condorelli G, Bellazzi R, Puca AA.** (2009). Association of the FOXO3A locus with extreme longevity in a southern Italian centenarian study. Rejuvenation Res. 2009 Apr; 12(2):95-104.

**4. Arum 0, Bonkowski MS, Rocha JS, Bartke A.** (2009). The growth hormone receptor gene-disrupted mouse fails to respond to an intermittent fasting diet. Aging Cell. 8(6):756-60.

**5. Arum 0, Rickman DJ, Kopchick JJ, Bartke A.** (2013). The slow-aging growth hormone receptor/binding protein gene-disrupted (GHR-KO) mouse is protected from aging-resultant neuromusculoskeletal frailty. AGE (Dordr). 2014 Feb;36(1):117-27. doi: 10.1007/s11357-013-9551-x. Epub 2013 Jul 4.

**6. Arum 0, Saleh JK, Boparai RK, Kopchick JJ, Khardori RK, Bartke A.** (2014). Preservation of blood glucose homeostasis in slow-senescing somatotrophism-deficient mice subjected to intermittent fasting begun at middle or old age. AGE (Dordr). 2014 May 1. [Epub ahead of print]

**7. Avogaro A, de Kreutzenberg SV, Fadini GP.** (2010). Insulin signaling and life span. Pflugers Arch. 2010 Jan;459(2):301-14. Epub 2009 Sep 13.

**8. Barbieri M, Boccardi V, Papa M, Paolisso G.** (2009). Metabolic journey to healthy longevity. Horm Res. 71 Suppl 1:24-7.

**9. Barbieri M, Bonafè M, Franceschi C, Paolisso G.** (2003). Insulin/IGF-I-signaling pathway: an evolutionarily conserved mechanism of longevity from yeast to humans. Am J Physiol Endocrinol Metab. 285(5):E1064-71.

**10. Barbieri M, Rizzo MR, Manzella D, Paolisso G.** (2001). Age-related insulin resistance: is it an obligatory finding? The lesson from healthy centenarians. Diabetes Metab Res Rev. 17(1):19-26.

**11. Bartke A, Brown-Borg H.** (2004). Life extension in the dwarf mouse. Curr Top Dev Biol. 63:189-225.

**12. Bartke A, Peluso MR, Moretz N, Wright C, Bonkowski M, Winters TA, Shanahan MF, Kopchick JJ, Banz WJ.** (2004). Effects of Soy-derived diets on plasma and liver lipids, glucose tolerance, and longevity in normal, long-lived and short-lived mice. Horm Metab Res. 36(8):550-8.

**13. Bartke A.** (2005a). Insulin resistance and cognitive aging in long-lived and short-lived mice. J Gerontol A Biol Sci Med Sci. 2005 Jan;60(1):133-4.

**14. Bartke A.** (2005b). Minireview: role of the growth hormone/insulin-like growth factor system in mammalian aging. Endocrinology. 146(9):3718-23.

**15. Bartke A.** (2006). Long-lived Klotho mice: new insights into the roles of IGF-1 and insulin in aging. Trends Endocrinol Metab. 2006 Mar;17(2):33-5. Epub 2006 Feb 3.

**16. Bartke A.** (2008). Insulin and aging. Cell Cycle. 7(21):3338-43.

**17. Bartke A.** (2011). Single-gene mutations and healthy ageing in mammals. Philos Trans R Soc Lond B Biol Sci.; 366(1561):28-34.

**18. Barzilai N, Bartke A.** (2009). Biological approaches to mechanistically understand the healthy life span extension achieved by calorie restriction and modulation of hormones. J Gerontol A Biol Sci Med Sci. 64(2):187-91.

**19. Barzilai N, Huffman DM, Muzumdar RH, Bartke A.** (2012). The critical role of metabolic pathways in aging. Diabetes. 2012 Jun;61(6):1315-22.

**20. Berryman DE, Christiansen JS, Johannsson G, Thorner MO, Kopchick JJ.** (2008). Role of the GH/IGF-1 axis in lifespan and healthspan: lessons from animal models. Growth Horm IGF Res.;18(6):455-71.

**21. Berryman DE, List EO, Coschigano KT, Behar K, Kim JK, Kopchick JJ.** (2004). Comparing adiposity profiles in three mouse models with altered GH signaling. Growth Horm IGF Res. 2004 Aug;14(4):309-18.

**22. Berryman DE, List EO, Palmer AJ, Chung MY, Wright-Piekarski J, Lubbers E, O'Connor P, Okada S, Kopchick JJ.** (2010). Two-year body composition analyses of long-lived GHR null mice. J Gerontol A Biol Sci Med Sci.; 65(1):31-40.

**23. Blüher M, Kahn BB, Kahn CR. (**2003). Extended longevity in mice lacking the insulin receptor in adipose tissue. Science.; 299(5606):572-4.

**24. Bonkowski MS, Rocha JS, Masternak MM, Al Regaiey KA, Bartke A.** (2006). Targeted disruption of growth hormone receptor interferes with the beneficial actions of calorie restriction. Proc Natl Acad Sci U S A. 103(20):7901-5.

**25. Boparai RK, Arum 0, Khardori R, Bartke A.** (2010)**.** Glucose homeostasis and insulin sensitivity in growth hormone-transgenic mice: a cross-sectional analysis. Biol Chem.; 391(10):1149-55.

**26. Brown-Borg HM, Borg KE, Meliska CJ, Bartke A.** (1996). Dwarf mice and the ageing process. Nature. 384(6604):33.

**27. Brown-Borg HM.** (2009). Hormonal control of aging in rodents: the somatotropic axis. Mol Cell Endocrinol. 299(1):64-71.

**28. Butler AA, Kozak LP.** (2010). A recurring problem with the analysis of energy expenditure in genetic models expressing lean and obese phenotypes. Diabetes. 59(2):323-9.

**29. Calnan DR, Brunet A.** (2008). The FoxO code. Oncogene. 2008 Apr 7;27(16):2276-88.

**30. Cangelosi PR.** (2011). Baby Boomers: are we ready for their impact on health care? J Psychosoc Nurs Ment Health Serv. 2011 Sep;49(9):15-7. Epub 2011 Aug 17.

**31. Carrillo AE, Flouris AD.** (2011). Caloric restriction and longevity: effects of reduced body temperature. Ageing Res Rev.;10(1):153-62.

**32. Centers for Disease Control and Prevention. Atlanta, GA: Centers for Disease Control and Prevention, US Department of Health and Human Services.** (2012). Diabetes Report Card 2012. (<http://www.cdc.gov/diabetes/pubs/pdf/DiabetesReportCard.pdf>).

**33. Centers for Disease Control and Prevention. Atlanta, GA: Centers for Disease Control and Prevention, US Department of Health and Human Services.** (2011). National Diabetes Fact Sheet, 2011. [(http://www.cdc.gov/diabetes/pubs/pdf/ndfs_2011.pdf)](file://C:\\AppData\\Roaming\\AppData\\Roaming\\AppData\\Roaming\\AppData\\Roaming\\AppData\\Roaming\\AppData\\Roaming\\AppData\\Roaming\\AppData\\Roaming\\AppData\\Roaming\\AppData\\Roaming\\AppData\\Roaming\\Microsoft\\AppData\\Roaming\\Microsoft\\Word\\(http:\\www.cdc.gov\\diabetes\\pubs\\pdf\\ndfs_2011.pdf)).

**34. Chatterjee R, Yeh HC, Edelman D, Brancati F.** (2011). Potassium and risk of Type 2 diabetes. Expert Rev Endocrinol Metab. 2011 Sep;6(5):665-672.

**35. Chen YF, Wu CY, Kao CH, Tsai TF.** (2010). Longevity and lifespan control in mammals: lessons from the mouse. Ageing Res Rev. 2010 Nov;9 Suppl 1:S28-35. Epub 2010 Aug 7.

**36. Choi D, Nguyen KT, Wang L, Schroer SA, Suzuki A, Mak TW, Woo M.** (2008). Partial deletion of Pten in the hypothalamus leads to growth defects that cannot be rescued by exogenous growth hormone. Endocrinology.; 149(9):4382-6.

**37. Choudhury, A. I., H. Heffron, M. A. Smith, H. Al-Qassab, A. W. Xu, C. Selman, M. Simmgen, M. Clements, M. Claret, G. Maccoll, D. C. Bedford, K. Hisadome, I. Diakonov, V. Moosajee, J. D. Bell, J. R. Speakman, R. L. Batterham, G. S. Barsh, M. L. Ashford, and D. J. Withers.** (2005). The role of insulin receptor substrate 2 in hypothalamic and beta cell function. J. Clin. Investig.; 115:940-950.

**38. Conover CA, Bale LK.** (2007). Loss of pregnancy-associated plasma protein A extends lifespan in mice. Aging Cell. 6(5):727-9.

**39. Conover CA, Mason MA, Levine JA, Novak CM.** (2008). Metabolic consequences of pregnancy-associated plasma protein-A deficiency in mice: exploring possible relationship to the longevity phenotype. J Endocrinol. 198(3):599-605.

**40.** **Conti B, Sanchez-Alavez M, Winsky-Sommerer R, Morale MC, Lucero J, Brownell S, Fabre V, Huitron-Resendiz S, Henriksen S, Zorrilla EP, de Lecea L, Bartfai T.** (2006). Transgenic mice with a reduced core body temperature have an increased life span. Science.;314(5800):825-8.

**41. Coschigano KT *et al.*** (1999). 81st Annual Meeting of The Endocrine Society, San Diego, CA.

**42. Coschigano KT, Clemmons D, Bellush LL, Kopchick JJ.** (2000). Assessment of growth parameters and life span of GHR/BP gene-disrupted mice. Endocrinology. 141(7):2608-13.

**43. Coschigano KT, Holland AN, Riders ME, List EO, Flyvbjerg A, Kopchick JJ.** (2003). Deletion, but not antagonism, of the mouse growth hormone receptor results in severely decreased body weights, insulin, and insulin-like growth factor I levels and increased life span. Endocrinology. 144(9):3799-810.

**44. Crawley JN.** (2007). What’s Wrong With My Mouse? Behavioral Phenotyping of Transgenic and Knockout Mice, Second Edition. John Wiley & Sons, Inc. Hoboken, NJ.

**45. Däumer C, Flachsbart F, Caliebe A, Schreiber S, Nebel A, Krawczak M.** (2014). Adjustment for smoking does not alter the FOXO3A association with longevity. AGE. April 2014. 36(2):911-921.

**46.** **Devedjian JC, George M, Casellas A, Pujol A, Visa J, Pelegrin M, Gros L, Bosch F.** (2000). Transgenic mice overexpressing insulin-like growth factor-II in β cells develop type 2 diabetes. J Clin Invest. 105:731–740.

**47. DiStefano PS, Curtis R, Geddes BJ.** (2007). Insulin resistance, glycemic control and adiposity: key determinants of healthy lifespan. Curr Alzheimer Res. 4(2):153-7.

**48. Dominguez LJ, Paolisso G, Barbagallo M.** (2010). Glucose control in the older patient: from intensive, to effective and safe. Aging Clin Exp Res. 2010 Aug;22(4):274-80. Epub 2009 Nov 17.

**49. Dominici FP, Hauck S, Argentino DP, Bartke A, Turyn D.** (2002). Increased insulin sensitivity and upregulation of insulin receptor, insulin receptor substrate (IRS)-1 and IRS-2 in liver of Ames dwarf mice. J Endocrinol. 173(1):81-94.

**50. Donlon TA, Curb JD, He Q, Grove JS, Masaki KH, Rodriguez B, Elliott A, Willcox DC, Willcox BJ**. (2012). FOXO3 gene variants and human aging: coding variants may not be key players. J Gerontol A Biol Sci Med Sci. doi:10.1093/gerona/gls067.

**51. Fang Y, Westbrook R, Hill C, Boparai RK, Arum 0, Spong A, Wang F, Javors MA, Chen J, Sun LY, Bartke A.** (2013). Duration of rapamycin treatment has differential effects on metabolism in mice. Cell Metab. 2013 Mar 5;17(3):456-62. doi: 10.1016/j.cmet.2013.02.008.

**52. Flachsbart F, Caliebe A, Kleindorp R, Blanché H, von Eller-Eberstein H, Nikolaus S, Schreiber S, Nebel A.** (2009). Association of FOXO3A variation with human longevity confirmed in German centenarians. Proc Natl Acad Sci U S A. 2009 Feb 24; 106(8):2700-5. Epub 2009 Feb 5.

**53. Flachsbart F, Moller M, Daeumer C, Gentschew L, Kleindorp R, Krawczak M, Caliebe A, Schreiber S, Nebel A**. (2012). Genetic investigation of FOXO3A requires special attention due to sequence homology with FOXO3B. Eur J Hum Genet. doi:10.1038/ejhg.2012.83

**54. Flurkey K, Papaconstantinou J, Miller RA, Harrison DE.** (2001). Lifespan extension and delayed immune and collagen aging in mutant mice with defects in growth hormone production. Proc Natl Acad Sci U S A. 98(12):6736-41.

**55. Fontana L, Klein S, Holloszy JO.** (2010). Effects of long-term calorie restriction and endurance exercise on glucose tolerance, insulin action, and adipokine production. Age (Dordr). 32(1):97-108.

**56. Gannon M, Shiota C, Postic C, Wright CV, Magnuson M.** (2000). Analysis of the Cre-mediated recombination driven by rat insulin promoter in embryonic and adult mouse pancreas. Genesis.; 26:139-142. 1

**57. Gerozissis K.** (2003). Brain insulin: regulation, mechanisms of action and functions. Cell. Mol. Neurobiol.; 23:1-25.

**58. Glantz SA.** (2002). Primer of Biostatistics, Fifth Edition. Chapter Two: How to Summarize Data. The McGraw-Hill Companies, Inc. New York, NY.

**59. Gong Z, Muzumdar RH.** (2012). Pancreatic function, type 2 diabetes, and metabolism in aging. Int J Endocrinol. 2012;2012:320482. Epub 2012 May 17.

**60. Greene DA.** (1986). Acute and chronic complications of diabetes mellitus in older patients. Am J Med. 1986 May 16;80(5A):39-53.

**61. Guo Y, Lu Y, Houle D, Robertson K, Tang Z, Kopchick JJ, Liu YL, Liu JL.** (2005). Pancreatic islet-specific expression of an insulin-like growth factor-I transgene compensates islet cell growth in growth hormone receptor gene-deficient mice. Endocrinology. 146(6):2602-9.

**62. Habener JF, Stanojevic V.** (2012). Alpha cells come of age. Trends in Endocrinol & Metab; 24(3):153-163.

**63. Hamlin CR, Kohn RR, Luschin JH.** (1975). Apparent accelerated aging of human collagen in diabetes mellitus. Diabetes. 1975 Oct;24(10):902-4.

**64. Harper JM, Durkee SJ, Smith-Wheelock M, Miller RA.** (2005). Hyperglycemia, impaired glucose tolerance and elevated glycated hemoglobin levels in a long-lived mouse stock. Exp Gerontol. 40(4):303-14.

**65. Holzenberger M, Dupont J, Ducos B, Leneuve P, Géloën A, Even PC, Cervera P, Le Bouc Y.** (2003). IGF-1 receptor regulates lifespan and resistance to oxidative stress in mice. Nature. 421(6919):182-7.

**66. Holzenberger M.** (2004). The GH/IGF-I axis and longevity. Eur J Endocrinol. 151 Suppl 1:S23-7.

**67. Iwersen M, Falkenberg U, Voigtsberger R, Forderung D, Heuwieser W.** (2009). Evaluation of an electronic cowside test to detect subclinical ketosis in dairy cows. J Dairy Sci.; 92(6):2618-24.

**68. Kaushik S, Cuervo AM.** (2012a). Chaperone-mediated autophagy: a unique way to enter the lysosome world. Trends Cell Biol. 2012 Aug;22(8):407-17. Epub 2012 Jun 27.

**69. Kaushik S, Cuervo AM.** (2012b). Chaperones in autophagy. Pharmacol Res. 2012 Dec;66(6):484-93. Epub 2012 Oct 8.

**70. Kent S.** (1976). Is diabetes a form of accelerated aging? Geriatrics. 1976 Nov;31(11):140, 145, 149-51.

**71. Kenyon CJ.** (2010). The genetics of ageing. Nature. 2010 Mar 25;464(7288):504-12. Erratum in: Nature. 2010 Sep 30;467(7315):622.

**72. Kinney BA, Coschigano KT, Kopchick JJ, Steger RW, Bartke A.** (2001a). Evidence that age-induced decline in memory retention is delayed in growth hormone resistant GH-R-KO (Laron) mice. Physiol Behav.; 72(5):653-60.

**73. Kinney BA, Meliska CJ, Steger RW, Bartke A.** (2001b). Evidence that Ames dwarf mice age differently from their normal siblings in behavioral and learning and memory parameters. Horm Behav.; 39(4):277-84.

**74. Kinney-Forshee BA, Kinney NE, Steger RW, Bartke A.** (2004). Could a deficiency in growth hormone signaling be beneficial to the aging brain? Physiol Behav.; 80(5):589-94.

**75. Kirkland JL, Peterson C.** (2009). Healthspan, translation, and new outcomes for animal studies of aging. J Gerontol A Biol Sci Med Sci.; 64(2):209-12.

**76. Knickman JR, Snell EK.** (2002). The 2030 problem: caring for aging baby boomers. Health Serv Res. 2002 Aug;37(4):849-84.

**77. Knight EM, Verkhratsky A, Luckman SM, Allan SM, Lawrence CB.** (2012). Hypermetabolism in a triple-transgenic mouse model of Alzheimer's disease. Neurobiol Aging. 2012 Jan;33(1):187-93. Epub 2010 Mar 31.

**78. Kurosu H, Yamamoto M, Clark JD, Pastor JV, Nandi A, Gurnani P, McGuinness OP, Chikuda H, Yamaguchi M, Kawaguchi H, Shimomura I, Takayama Y, Herz J, Kahn CR, Rosenblatt KP, Kuro-o M.** (2005). Suppression of aging in mice by the hormone Klotho. Science. 2005 Sep 16;309(5742):1829-33. Epub 2005 Aug 25.

**79. Landsberg L. (**1986). Diet, Obesity and Hypertension: An hypothesis involving insulin, the sympathetic nervous system, and adaptive thermogenesis. Quarterly Journal of Medicine, New Series 61. No. 236, pp. 1081-1090.

**80. Lane MA, Baer DJ, Rumpler WV, Weindruch R, Ingram DK, Tilmont EM, Cutler RG, Roth GS.** (1996). Calorie restriction lowers body temperature in rhesus monkeys, consistent with a postulated anti-aging mechanism in rodents. Proc Natl Acad Sci U S A.;93(9):4159-64.

**81. Li Y, Wang WJ, Cao H, Lu J, Wu C, Hu FY, Guo J, Zhao L, Yang F, Zhang YX, et al.** (2009). Genetic association of FOXO1A and FOXO3A with longevity trait in Han Chinese populations. Hum Mol Genet. 2009 Dec 15; 18(24):4897-904. Epub 2009 Sep 29.

**82. Liu JL, Coschigano KT, Robertson K, Lipsett M, Guo Y, Kopchick JJ, Kumar U, Liu YL.** (2004). Disruption of growth hormone receptor gene causes diminished pancreatic islet size and increased insulin sensitivity in mice. Am J Physiol Endocrinol Metab. 287(3):E405-13.

**83. Longo VD, Finch CE.** (2003). Evolutionary medicine: from dwarf model systems to healthy centenarians? Science. 299(5611):1342-6.

**84. Masoro EJ.** (2005). Overview of caloric restriction and ageing. Mech Ageing Dev. 126(9):913-22.

**85. Masternak MM, Bartke A, Wang F, Gesing A, Fang Y, Salmon AB, Hughes LF, Liberati T, Boparai R, Kopchick JJ, Westbrook R.** (2012). Metabolic effects of intra-abdominal fat in GHRKO mice. Aging Cell. 2012 Feb;11(1):73-81. Epub 2011 Nov 28.

**86. Masternak MM, Panici JA, Bonkowski MS, Hughes LF, Bartke A.** (2009). Insulin sensitivity as a key mediator of growth hormone actions on longevity. J Gerontol A Biol Sci Med Sci. 2009 May;64(5):516-21. doi: 10.1093/gerona/glp024. Epub 2009 Mar 20.

**87. Miller RA, Burke D, Nadon N.** (1999). Announcement: four-way cross mouse stocks: a new, genetically heterogeneous resource for aging research. J Gerontol A Biol Sci Med Sci. 1999 Aug; 54(8):B358-60.

**88. Miller RA.** (2009). "Dividends" from research on aging--can biogerontologists, at long last, find something useful to do? J Gerontol A Biol Sci Med Sci. 64(2):157-60. Epub 2009 Feb 18.

**89. Monickaraj F, Aravind S, Gokulakrishnan K, Sathishkumar C, Prabu P, Prabu D, Mohan V, Balasubramanyam M.** (2012). Accelerated aging as evidenced by increased telomere shortening and mitochondrial DNA depletion in patients with type 2 diabetes. Mol Cell Biochem. 2012 Jun; 365(1-2):343-50. Epub 2012 Mar 13.

**90. Mookerjee SA, Divakaruni AS, Jastroch M, Brand MD.** (2010). Mitochondrial uncoupling and lifespan. Mech Ageing Dev.; 131(7-8):463-72.

**91. Muzumdar R, Allison DB, Huffman DM, Ma X, Atzmon G, Einstein FH, Fishman S, Poduval AD, McVei T, Keith SW, Barzilai N.** (2008). Visceral adipose tissue modulates mammalian longevity. Aging Cell. 7(3):438-40.

**92. Nass R, Johannsson G, Christiansen JS, Kopchick JJ, Thorner MO.** (2009). The aging population--is there a role for endocrine interventions? Growth Horm IGF Res.; 19(2):89-100.

**93. Nguyen KT, Tajmir P, Lin CH, Liadis N, Zhu XD, Eweida M, Tolasa-Karaman G, Cai F, Wang R, Kitamura T, Belsham DD, Wheeler MB, Suzuki A, Mak TW, Woo M.** (2006). Essential role of Pten in body size determination and pancreatic beta-cell homeostasis in vivo. Mol Cell Biol.; 26(12):4511-8.

**94. Olshansky SJ, Perry D, Miller RA, Butler RN.** (2007). Pursuing the longevity dividend: scientific goals for an aging world. Ann N Y Acad Sci. 1114:11-3.

**95. Pawlikowska L, Hu D, Huntsman S, Sung A, Chu C, Chen J, Joyner AH, Schork NJ, Hsueh WC, Reiner AP, Psaty BM, Atzmon G, Barzilai N, Cummings SR, Browner WS, Kwok PY, Ziv E.** (2009). Association of common genetic variation in the insulin/IGF1 signaling pathway with human longevity. Aging Cell. 8(4):460–472.

**96. Poehlman ET, Dvorak RV.** (1998). Energy expenditure in Alzheimer's disease. J Nutr Health Aging. 1998;2(2):115-8.

**97. Redman LM, Ravussin E.** (2009). Endocrine alterations in response to calorie restriction in humans. Mol Cell Endocrinol. 299(1):129-36.

**98. Ricketts TC.** (2011). The health care workforce: will it be ready as the boomers age? A review of how we can know (or not know) the answer. Annu Rev Public Health. 2011;32:417-30.

**99. Roth, GS, Lane MA, Ingram DK, Mattison JA, Elahi D, Tobin JD, Muller D, Metter EJ.** (2002). Biomarkers of Caloric Restriction May Predict Longevity in Humans. Science; 297(5582):811.

**100. Rozing MP, Westendorp RG, de Craen AJ, Frölich M, de Goeij MC, Heijmans BT, Beekman M, Wijsman CA, Mooijaart SP, Blauw GJ, Slagboom PE, van Heemst D; Leiden Longevity Study Group.** (2010). Favorable glucose tolerance and lower prevalence of metabolic syndrome in offspring without diabetes mellitus of nonagenarian siblings: the Leiden longevity study. J Am Geriatr Soc. 58(3):564-9.

**101. Russell SJ, Kahn CR.** (2007). Endocrine regulation of ageing. Nat Rev Mol Cell Biol. 8(9):681-91.

**102. Selman C, Lingard S, Choudhury AI, Batterham RL, Claret M, Clements M, Ramadani F, Okkenhaug K, Schuster E, Blanc E, Piper MD, Al-Qassab H, Speakman JR, Carmignac D, Robinson IC, Thornton JM, Gems D, Partridge L, Withers DJ.** (2008). Evidence for lifespan extension and delayed age-related biomarkers in insulin receptor substrate 1 null mice. FASEB J. 22(3):807-18.

**103. Selman C, Tullet JM, Wieser D, Irvine E, Lingard SJ, Choudhury AI, Claret M, Al-Qassab H, Carmignac D, Ramadani F, Woods A, Robinson IC, Schuster E, Batterham RL, Kozma SC, Thomas G, Carling D, Okkenhaug K, Thornton JM, Partridge L, Gems D, Withers DJ.** (2009). Ribosomal protein S6 kinase 1 signaling regulates mammalian life span. Science. 326(5949):140-4.

**104. Selman C, Withers DJ.** (2011). Mammalian models of extended healthy lifespan. Philos Trans R Soc Lond B Biol Sci.; 366(1561):99-107.

**105. Singh R, Cuervo AM.** (2011)**.** Autophagy in the cellular energetic balance. Cell Metab. 2011 May 4;13(5):495-504.

**106. Soerensen M, Dato S, Christensen K, McGue M, Stevnsner T, Bohr VA, Christiansen L.** (2010). Replication of an association of variation in the FOXO3A gene with human longevity using both case-control and longitudinal data. Aging Cell. 2010 Dec; 9(6):1010-7. Epub 2010 Oct 21.

**107. Taguchi A, Wartschow LM, White MF.** (2007). Brain IRS2 signaling coordinates life span and nutrient homeostasis. Science. 317(5836):369-72.

**108. Tatar M.** (2009). Can we develop genetically tractable models to assess healthspan (rather than life span) in animal models? J Gerontol A Biol Sci Med Sci; 64(2):161-3.

**109. Unger RH, Cherrington AD.** (2012). Glucagonocentric restructuring of diabetes: a pathophysiologic and therapeutic makeover. J Clin Invest; 122:4-12.

**110. Unger RH, Orci L.** (2010). Paracrinology of islets and the paracrinopathy of diabetes. Proc Natl Acad Sci;107:16009-16012.

**111. Valera A, Solanes G, Fernandez-Alvarez J, Pujol A, Ferrer J, Asins G, Gomis R, Bosch F.** (1994). Expression of GLUT-2 antisense RNA in β cells of transgenic mice leads to diabetes. J Biol Chem 269:28543–28546.

**112. Viljoen A, Sinclair AJ.** (2011). Diabetes and insulin resistance in older people. Med Clin North Am. 2011 May;95(3):615-29, xi-ii.

**113. Wallace TM, Meston NM, Gardner SG, Matthews DR.** (2001). The hospital and home use of a 30-second hand-held blood ketone meter: guidelines for clinical practice. Diabet Med.; 18(8):640-5.

**114. Wallace TM, Meston NM, Gardner SG, Matthews DR.** (2008). Use of a hand-held meter for the measurement of blood beta-hydroxybutyrate in dogs and cats. Journal of Veterinary Emergency and Critical Care. 18(1):86-87.

**115. Warner HR, Sierra F.** (2009). The longevity dividend: why invest in basic aging research? Can J Aging. 28(4):391-4; French 395-8.

**116. Westbrook R, Bonkowski MS, Strader AD, Bartke A.** (2009). Alterations in oxygen consumption, respiratory quotient, and heat production in long-lived GHRKO and Ames dwarf mice, and short-lived bGH transgenic mice. J Gerontol A Biol Sci Med Sci. 64(4):443-51.

**117. Wijsman CA, Rozing MP, Streefland TC, le Cessie S, Mooijaart SP, Slagboom PE, Westendorp RG, Pijl H, van Heemst D; Leiden Longevity Study group.** (2011). Familial longevity is marked by enhanced insulin sensitivity. Aging Cell. 2011 Feb;10(1):114-21. Epub 2010 Dec 7.

**118. Willcox BJ, Donlon TA, He Q, Chen R, Grove JS, Yano K, Masaki KH, Willcox DC, Rodriguez B, Curb JD.** (2008). FOXO3A genotype is strongly associated with human longevity. Proc Natl Acad Sci U S A. 2008 Sep 16;105(37):13987-92. Epub 2008 Sep 2.

**119. Zhou Y, Xu BC, Maheshwari HG, He L, Reed M, Lozykowski M, Okada S, Cataldo L, Coschigamo K, Wagner TE, Baumann G, Kopchick JJ.** (1997). A mammalian model for Laron syndrome produced by targeted disruption of the mouse growth hormone receptor/binding protein gene (the Laron mouse). Proc Natl Acad Sci U S A. 94(24):13215-20.

**120. Ziv E, Hu D.** (2011). Genetic variation in insulin/IGF-1 signaling pathways and longevity. Ageing Res Rev. 2011 Apr;10(2):201-4. Epub 2010 Sep 22.
